# Exciplex, Not Heavy-Atom Effect, Controls the Triplet Dynamics of a Series of Sulfur-Containing Thermally Activated Delayed Fluorescence Molecules

**DOI:** 10.1021/acs.chemmater.4c00850

**Published:** 2024-08-02

**Authors:** Saliha Öner, Suman Kuila, Kleitos Stavrou, Andrew Danos, Mark A. Fox, Andrew P. Monkman, Martin R. Bryce

**Affiliations:** † Department of Chemistry, Durham University, Stockton Road, Durham DH1 3LE, U.K.; ‡ Department of Physics, Durham University, Stockton Road, Durham DH1 3LE, U.K.

## Abstract

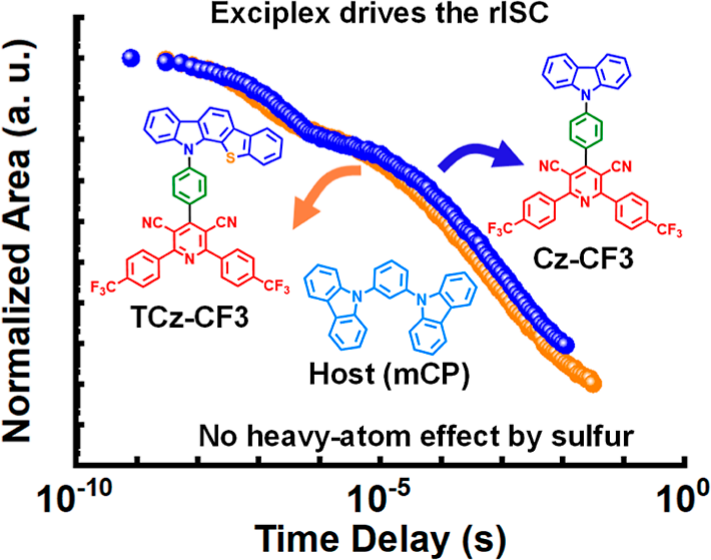

The efficiency of thermally activated delayed fluorescence (TADF) in organic materials relies on rapid intersystem crossing rates and fast conversion of triplet (T) excitons into a singlet (S) state. Heavy atoms such as sulfur or selenium are now frequently incorporated into TADF molecular structures to enhance these properties by increased spin–orbit coupling [spin orbit coupling (SOC)] between the T and S states. Here a series of donor–acceptor (D–A) molecules based on 12*H*-benzo[4,5]thieno[2,3-*a*]carbazole and dicyanopyridine is compared with their nonsulfur control molecules designed to probe such SOC effects. We reveal that unexpected intermolecular interactions of the D–A molecules with carbazole-containing host materials instead serve as the dominant pathway for triplet decay kinetics in these materials. In-depth photophysical and computational studies combined with organic light emitting diode measurements demonstrate that the anticipated heavy-atom effect from sulfur is overshadowed by exciplex formation. Indeed, even the unsubstituted acceptor fragments exhibit pronounced TADF exciplex emission in appropriate carbazole hosts. The intermolecular charge transfer and TADF in these systems are further confirmed by detailed time-dependent density functional theory studies. This work demonstrates that anticipated heavy-atom effects in TADF emitters do not always control or even impact the photophysical and electroluminescence properties.

## Introduction

The spin multiplicity of molecular excited states is a crucial factor in optoelectronic device performance and applications.^1,2^ In organic light emitting diodes (OLEDs) electrical excitation produces singlet (S) and triplet (T) states in a 1:3 ratio from the random recombination of uncorrelated electrons and holes. Radiative decay of singlet excitons to the ground state leads to fluorescence while spin conservation normally forbids emission from the triplet states, severely limiting the achievable electroluminescence efficiency. Consequently, extensive research has sought to find ways to convert normally “dark” triplet excited states into emissive states. Organometallic phosphors that incorporate heavy metals such as iridium and platinum can achieve efficient triplet emission and fast intersystem crossing (ISC) to equilibrate T and S states due to strong spin–orbit coupling [spin orbit coupling (SOC)] induced by the metal.^3^ More recently, research has focused on all-organic thermally activated delayed fluorescence (TADF, previously known as E-type DF) materials, which instead convert triplets into singlets which then emit.^4^

TADF emitters can be designed as all-organic or organometallic compounds. Organometallic compounds having coplanar conformations have been reported as efficient TADF emitters.^5−7^ Efficient TADF emission from all-organic compounds exhibiting a high reverse intersystem crossing (rISC) rate usually features near-orthogonally linked donor–acceptor (D–A) subunits that result in a small energy difference between the S and T excited states (Δ*E*_ST_) of charge transfer (CT) orbital character.^8−15^

This effect is rationalized by a small exchange energy between the electrons residing in spatially separated (and electronically decoupled) highest occupied and lowest unoccupied molecular orbitals (HOMO and LUMO) that are centered respectively on the D and A units of the molecules. This, however, also means that the singlet and triplet CT orbitals become degenerate, and SOC transitions between them require an additional mediator triplet state of different orbital character to facilitate vibronically coupled SOC for efficient rISC.^16,17^ The extent of S-T mixing (λ) that supports rISC is determined by both the spin–orbit coupling between the singlet and triplet states (H_SOC_) and their energy separation (Δ*E*_ST_) as λ ∝ H_SOC_/Δ*E*_ST_. SOC itself depends on the nature of the orbitals as governed by El-Sayed’s rule,^18^ and also the atomic number of any elements participating in the HOMO–LUMO electronic distribution. Heavy nuclei such as group 17 elements (e.g., Br and I) have therefore been attached to molecules to enhance SOC and consequently obtain faster ISC and rISC rates.^19−22^ However, due to weak C–Br and C–I bond energies, OLEDs incorporating these halogenated molecules can suffer from fast degradation and extensive efficiency roll-off,^23^ although this is not necessarily the case with chloride-substituted emitters.^20^

More recently, chalcogen atoms such as S and Se have been embedded in the D component of D–A molecules to achieve better device performance. However, the heavy-atom effects using these elements are often ambiguous due to their simultaneous impacts on electronic properties such as Δ*E*_ST_ and more extensive investigations are needed to fully understand their role in TADF.^24−29^ In this regard, it has previously been shown that the DF efficiency did not significantly improve when sulfur was replaced by selenium in materials featuring phenothiazine/phenoselenazine donor units.^30−32^ Computational modeling has also highlighted the importance of the precise location of the heavy atom(s) within the molecule and the conformational effects they induce, leading to multifaceted impacts on both the electronic properties and SOC matrix elements that are difficult to disentangle experimentally.^29,30,33^ Nonetheless, rapid advances are being made in inserting chalcogen atoms into both D–A and multiresonance TADF molecules, with the aim of understanding the underlying photophysics and facilitating new molecular designs for device engineering.^24−27,34^

**Figure 1:**
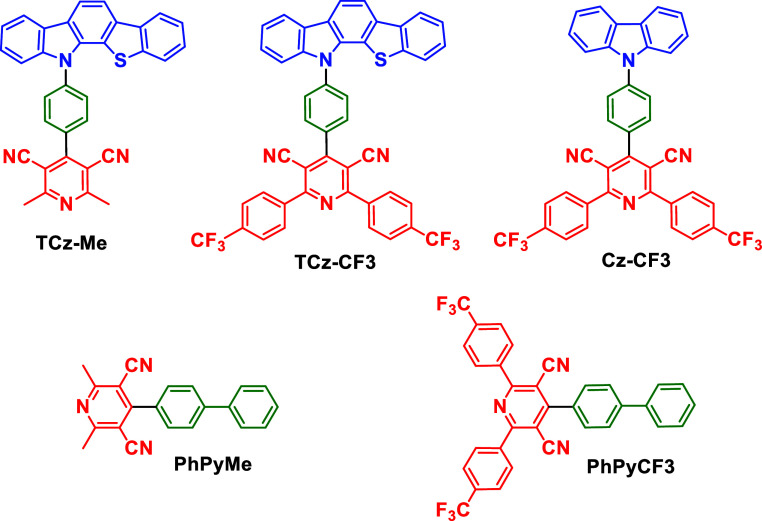
Molecular structures of the donor–acceptor TADF emitters (**TCz-Me**, **TCz-CF3**, and **Cz-CF3**) and the acceptor-only systems (**PhPyMe** and **PhPyCF3**) studied in this work.

With this initial aim, we report here a systematic experimental and computational study of new benzo[4,5]thieno[2,3-*a*]carbazole (TCz)- and carbazole (Cz)-derived TADF molecules, featuring dicyanopyridine acceptors (Figure 1). The singlet–triplet gap was rationally tuned by changing the acceptor strength to obtain blue and green TADF emission from structurally similar TCz-derived emitters. The heavy-atom-free carbazole analog **Cz-CF3** was studied as a reference compound alongside **TCz-CF3**, with the aim of understanding the impacts of heavy-atom insertion upon ISC/rISC and TADF efficiency. However, very strong host-dependent tuning of the TADF properties was observed, which we attribute to intermolecular exciplex formation between the D–A molecules and carbazole-containing hosts. To conclusively establish this exciplex channel, acceptor-only molecules **PhPyMe** and **PhPyCF3** were also synthesized and shown to exhibit TADF even in the absence of a covalently linked donor, but only in carbazole-based hosts. The optical properties in doped films were also shown to translate into green-emitting OLEDs which reach EQE_max_ of 16.2% for **Cz-CF3** and 12.7% for **TCz-CF3**. These results demonstrate an important case study of unexpected intermolecular effects completely masking any anticipated heavy-atom effects in these sulfur-containing TADF molecules.

## Results and Discussion

### Molecular Design and Synthesis

Planar fused carbazole derivatives are attracting great attention as donors for efficient TADF emitters and OLED applications.^35^ Their inherent rigidity^36^ helps to support desirable narrowband emission,^37^ small Δ*E*_ST_, and high photoluminescence quantum yield (PLQY).^38,39^ These chemically versatile structures often feature indolocarbazole,^40−42^ indenocarbazole,^43,44^ benzofurocarbazole,^45,46^ or benzothienocarbazole.^45,47,48^ It has also been shown that extending π–conjugation by fusing a benzothieno group onto carbazole increases the bond dissociation energy of the pendant N–C (donor–acceptor) bond by approximately 10% in the anionic state.^49^ In addition, due to the perpendicular arrangement of the donor in D–A systems, the fused S atom of benzo[4,5]thieno[2,3-*a*]carbazole will reside close to the D–A bridge across which the CT excited states form, and hence have the opportunity to impact the photophysical properties. In terms of device performance, Adachi et al. reported that OLEDs with the benzothienocarbazole-derived blue TADF emitter BTCZPZ1 had EQE_max_ of 21.1%, which was considerably higher than the analogous benzofurocarbazole BFCZPZ1-based device (EQE_max_ 6.5%).^48^ It is worth noting that the BTCZPZ1 showed substantially higher PLQY (91%) than the BFCZPZ1 (68%) in doped PPT [2,8-bis(diphenyl-phosphoryl)-dibenzo[*b,d*]thiophene] films. Conversely, another study reported that the EQE_max_ of a device based on the benzothienocarbazole derivative 12BTCzTPN (8.3%, PLQY 21.5%) is lower than the carbazole counterpart (EQ*E*_max_ 14.0%, PLQY = 27.5%) due to the lower PLQY of 12BTCzTPN. However, the 12BTCzTPN device showed slightly improved efficiency roll-off due to its higher rISC rate, which was attributed to the heavy-atom effect of the sulfur.^28^ Lee et al. reported that red hyperfluorescent OLEDs fabricated with three benzothienocarbazole-based emitters (PLQY = 25–36%) showed slightly higher EQ*E*_max_ values in the range of 12.3–14.7% compared to a carbazole counterpart (EQE_max_ 11.3%).^50^ These benzothienocarbazole-based derivatives also showed shorter DF lifetimes as compared to their nonsulfur TADF congeners. Therefore, new insights into benzothienocarbazole as a donor group are clearly of interest in the design of TADF emitters, as its overall effects compared to carbazole remain difficult to predict. Simultaneously, recent studies have established dicyanopyridine as a suitable acceptor for OLEDs with different colored emission.^51−58^ In that context, here we combine benzothieno[2,3-*a*]carbazole (TCz) donor and dicyanopyridine acceptors to obtain new blue and green TADF emitters **TCz-Me**, and **TCz-CF3**, along with the heavy-atom-free carbazole analog **Cz-CF3**, and the acceptor-only molecules **PhPyMe** and **PhPyCF3**. The structures of the new molecules studied in this work are shown in Figure 1.

TCz has a deep HOMO energy level and hence is potentially ideal for developing blue TADF emitters.^45,47,48^ Therefore, we first designed **TCz-Me** using TCz as the donor unit and a known weakly electron-deficient dicyanopyridine acceptor. Lee et al. have previously synthesized TCz as the donor part of TADF emitters.^28,45^ In their method, dibenzo[*b,d*]thiophen-3-ylboronic acid was reacted with 1-bromo-2-nitrobenzene by Suzuki–Miyaura coupling and then reductive cyclization was performed by using triphenylphosphine in *o*-DCB. The reaction provided the desired product TCz (12*H*-benzo[4,5]thieno[2,3-*a*]carbazole, 12BTCz) and its isomer (11*H*-benzo[4,5]thieno[3,2-*b*]carbazole, 11BTCz). To prevent formation of the undesired isomeric impurity, we used an alternative route to obtain the TCz donor (see Supporting Information, Scheme S1). Benzo[*b*]thiophene was reacted with cyclobutanone to give 1-(benzo[*b*]thiophen-2-yl)cyclobutanol which then underwent tandem oxidative ring opening and cyclization by using ammonium cerium(IV) nitrate (CAN).^59^ The product was then reacted with phenylhydrazine (Fischer indolization) followed by in situ oxidation with *p*-chloranil to obtain the desired TCz isomer exclusively in 31% overall yield for the last two steps.

The acceptor 4-(4′-bromophenyl)-2,6-dimethylpyridine-3,5-dicarbonitrile was synthesized by the literature route.^54^ The Buchwald–Hartwig N–C coupling reaction to give **TCz-Me** proceeded by using Pd_2_(dba)_3_, Xantphos and NaO*t*Bu in toluene. The acceptor strength was later enhanced by replacing the Me groups with more electron-deficient *p*-(trifluoromethyl)phenylene units to obtain **TCz-CF3**, incorporating the new acceptor, as shown in Figure 1 and Supporting Information It is worth noting that the solubility of **TCz-CF3** is considerably improved compared to **TCz-Me** in many organic solvents. Detailed information on the synthesis and characterization of **TCz-Me**, **TCz-CF3**, **Cz-CF3**, **PhPyMe** and **PhPyCF3** including the X-ray crystal structures of **Cz-CF3** and **PhPyMe** is given in the Supporting Information (Figures S1–S4 and Tables S1–S2). Hybrid density functional theory (DFT) computational data on the ground state (S_0_) geometries of **TCz-Me**, **TCz-CF3**, **Cz-CF3**, **PhPyMe** and **PhPyCF3** are also described in the Supporting Information (Figures S5–S7 and Tables S3–S4).

### Thermal and Electrochemical Properties

**TCz-Me**, **Cz-CF3** and **TCz-CF3** showed high thermal stabilities, where the 5% weight losses were around 230, 378, and 300 °C, respectively (Figure S1). These temperatures are higher than OLED working temperatures and the evaporation temperature of the materials used, therefore the compounds have sufficient thermal stability in terms of device applications. Cyclic voltammetry was used to investigate the electrochemical properties of these molecules (Figures S2 and S3 and Supporting Information for experimental details). Based on the oxidation waves, the calculated HOMO energy levels were −5.65, −5.66, and −5.78 eV for **TCz-Me**, **TCz-CF3**, and **Cz-CF3**, respectively, indicating the higher electron-donating ability of the TCz containing derivatives. The LUMO energy levels calculated by using the reduction potentials of CV were −2.77, −3.18, and −3.16 eV for **TCz-Me**, **TCz-CF3**, and **Cz-CF3**, respectively, showing the predicted better electron accepting ability of the −CF3 containing derivatives (Table S1). The LUMO values obtained instead from the optical bandgap (E_g_, opt) and the HOMO energy levels were calculated to be −2.65, −2.96, and −2.98 eV for **TCz-Me**, **TCz-CF3**, and **Cz-CF3**, respectively (Table S1). Consecutive cyclic measurements (6 cycles) indicated that all three molecules have stable oxidation/reduction waves and high electrochemical stabilities (Figure S3).

### Optical Properties

The absorption and steady-state emission spectra of **TCz-Me**, **TCz-CF3**, and **Cz-CF3** in dilute solutions were recorded (Figure S8). **TCz-Me** in toluene shows absorbance due to π–π* locally excited states in the 300–375 nm region, and a weaker CT absorbance at 375–425 nm. The absorption bands observed at 340 and 370 nm are characteristic of thienocarbazole groups.^49^ The increased electron deficiency of the –CF_3_ groups red-shifts the CT absorption to 375–450 nm in **TCz-CF3**, although a substantial change was not observed in its locally excited absorption wavelength. A strong solvatochromic effect in the photoluminescence (PL) confirmed the CT character of the excited states. The larger red-shift in the PL spectra of **TCz-CF3** is commensurate with increased acceptor strength, compared to **TCz-Me**.

**Figure 2:**
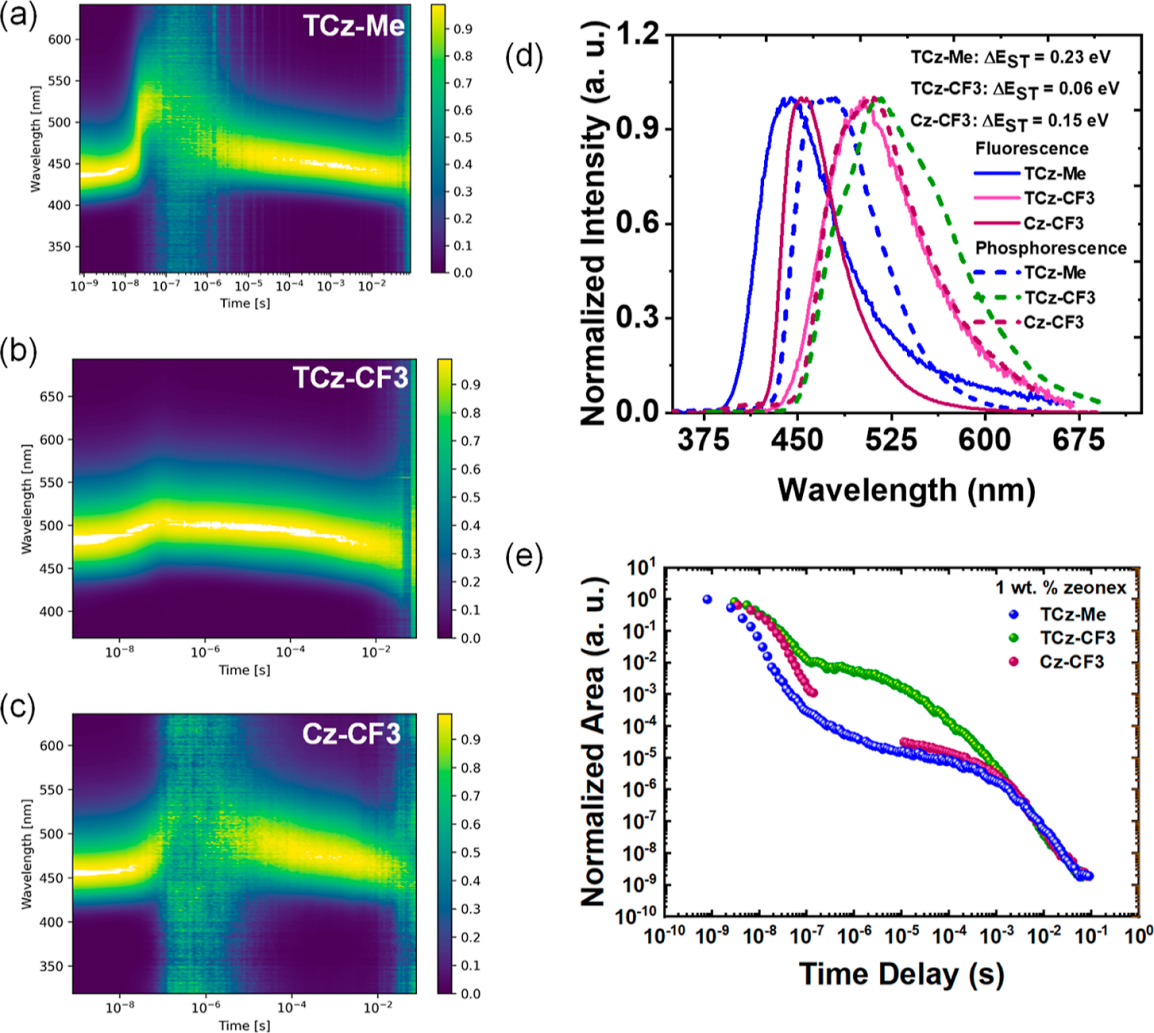
Contour plots of time-resolved emission spectra of (a) **TCz-Me** (b) **TCz-CF3** and (c) **Cz-CF3** films doped in 1 wt % zeonex at room temperature. (d) Steady-state photoluminescence (room temperature) and phosphorescence (80 K, 80 ms delay) in 1 wt % zeonex films. Phosphorescence for **TCz-CF3** was measured at 20 K (80 ms delay) to avoid any delayed emission contribution at low temperature. (e) Time-resolved emission decay of the same films at room temperature. λ_exc_ = 355 nm.

Steady-state and time-resolved spectra in 1 wt % zeonex films (Figure 2a–d) along with emission intensity decays (Figure 2e) were then investigated. **TCz-Me** at room temperature showed deep-blue emission (PL onset at 395 nm) with a DF component in addition to prompt fluorescence (PF). A significant red-shift was observed toward the end of the PF emission (∼100 ns), although this was reversed at longer time-delays with the DF having the same onset as the initial PF (Figures 2a and S9). This red-shifted emission could be due to a combination of typical D–A dihedral angle distributions in the film leading to dispersion in CT energies,^57,60−64^ and/or the formation of aggregates of the poorly soluble **TCz-Me** molecules.^65^ It is worth noting that, the dynamic spectral shift is observed in many TADF compounds when doped in rigid polymer hosts like zeonex. In the solid state, the immobile host molecules lock the emitter in different conformations, leading to a broad range of donor–acceptor (D–A) twist angles, singlet–triplet energy gaps, radiative decay rates (*k*_r_), and emission wavelengths. Unlike in solution, where solvent molecules can realign around the emitters, the PF in rigid hosts initially shows a blue-shift due to the largest S–T gaps and gradually red-shifts, while the DF regime shows an opposite shift due to the smallest Δ*E*_ST_ and fastest DF in larger twist-angle conformations.^62−64^ Nonetheless, the weak and long-lived nature of the TADF from **TCz-Me** is consistent with its large Δ*E*_ST_ (0.23 eV), calculated from the difference in the onsets of the room temperature steady-state fluorescence (*E*_S_) and phosphorescence measured at 80 K with 80 ms delay (*E*_T_, Figure 2d and Table S5). Similar zeonex films of **TCz-CF3** instead showed very strong and considerably shorter-lived TADF. This enhancement in TADF intensity and rISC kinetics is in line with the smaller Δ*E*_ST_ (0.06 eV) for **TCz-CF3**, consisting of a stronger acceptor unit and hence with a red-shifted PL onset compared to **TCz-Me**, significantly improving the close-alignment of ^1^CT–^3^E levels (Figure 2d). However, very dynamic changes were observed in the onset of the CT emission across the DF regime, as expected for flexible D–A molecules with a phenylene spacer (Figures 2a–c and S9).

Benzothienocarbazoles are relatively unexplored donor systems for designing TADF emitters,^28,45,48,50^ and so it was of particular interest to isolate any potential heavy-atom effect of sulfur by comparison with the parent carbazole analogue **Cz-CF3**. Due to the relatively weaker donor strength of Cz compared to TCz, 1 wt % zeonex films of **Cz-CF3** showed deeper-blue emission compared to **TCz-CF3** and with a relatively large Δ*E*_ST_ of 0.15 eV (Figure 2d, Table S5). Therefore, similar to **TCz-Me**, a long-lived weak TADF component was observed at RT, with a broad distribution of time-resolved PL spectra (Figures 2c, S9c and S10c).

**Figure 3:**
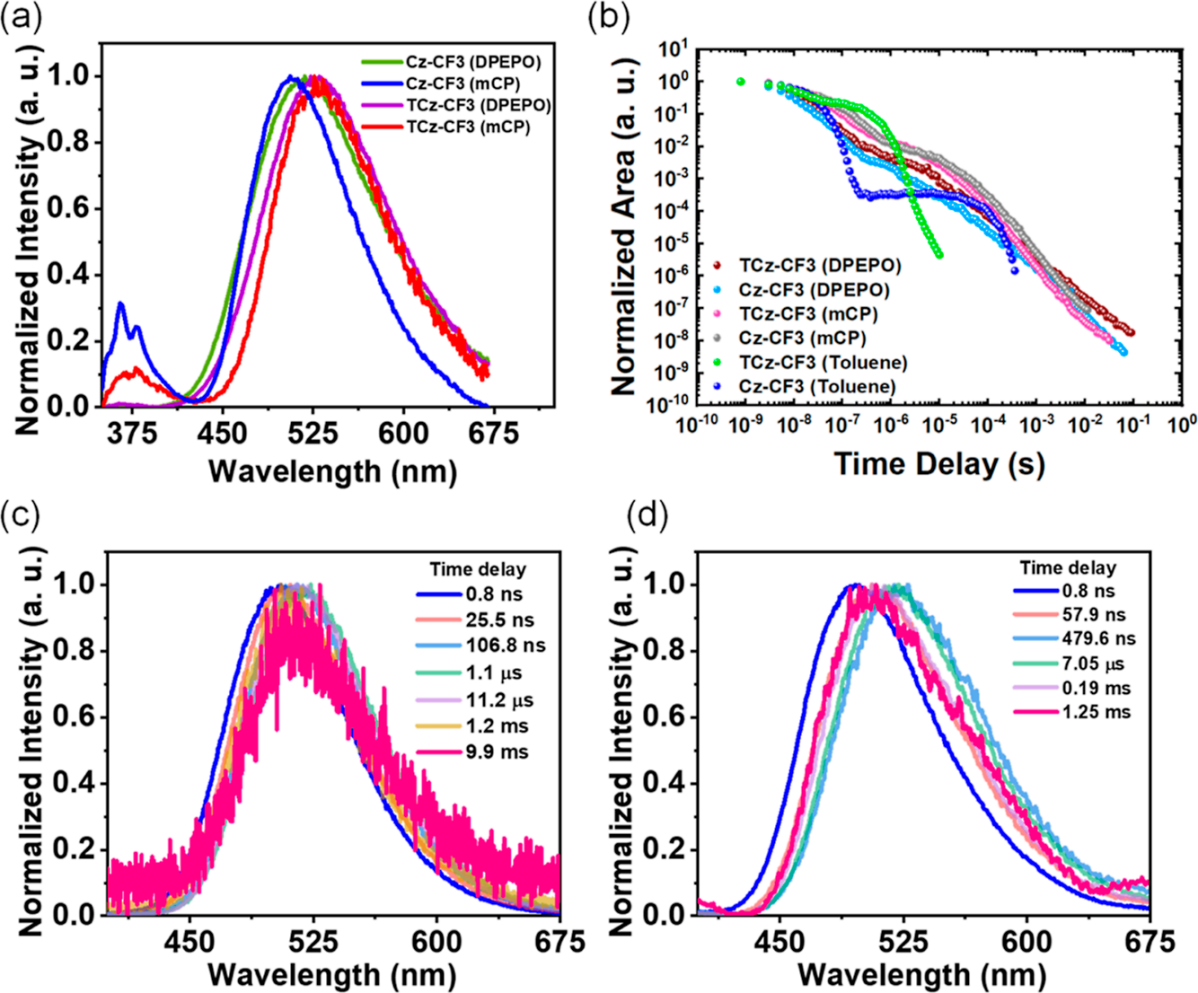
(a) Steady-state emission spectra of **TCz-CF3** and **Cz-CF3** doped in mCP and DPEPO (10 wt %). (b) Time-resolved emission decay of **TCz-CF3** and **Cz-CF3** in solid films (10 wt %) and dilute toluene (degassed, 50 μM) at room temperature. Time resolved emission spectra of (c) **TCz-CF3** and (d) **Cz-CF3** films doped in 10 wt % mCP, obtained at different delay times at RT. λ_exc_ = 355 nm.

Films of **TCz-Me** doped at 10 wt % in the high triplet energy OLED-compatible host DPEPO (bis[2-(diphenylphosphino)phenyl]ether oxide), T_1_ = 3.0 eV, dielectric constant (ε = 6.12)^66^ were also studied (Figure S11). The slightly increased TADF intensity which was observed could be due to the comparatively higher emitter concentration in DPEPO, with respect to the 1 wt % zeonex films, leading to enhanced intermolecular interactions arising from a small molecular matrix effect. This can potentially generate additional triplet recycling channels leading to better DF intensity. Similarly, both **TCz-CF3** and **Cz-CF3** showed clear DF although with moderate photoluminescence quantum yields (PLQYs, Φ_PL_ = 39% and 53% for **TCz-CF3** and **Cz-CF3**, respectively) in DPEPO (Table S5 and Figure S12). From the low temperature (80 K) phosphorescence measurements in DPEPO, both derivatives showed small singlet–triplet gaps (Δ*E*_ST_ = 0.06 and 0.11 eV, for **TCz-CF3** and **Cz-CF3**, respectively) suitable for efficient triplet recycling (Figures S12 and S13). Notably, 10 wt % DPEPO films of **Cz-CF3** showed a substantial red-shift as the singlet CT state (^1^CT) is more stabilized in DPEPO, having higher dielectric constant (ε = 6.12) and leading to a relatively smaller Δ*E*_ST_ of 0.11 eV, compared to the corresponding zeonex films with ε = 2.13 and Δ*E*_ST_ of 0.15 eV. The ^3^LE triplet state onset energy values remains unaffected by the choice of host (Figure 3a, Table S5, Figures S10 and S12).^67^

The emission characteristics of these materials were then studied in another high triplet-energy OLED-compatible host, mCP [1,3-bis(*N*-carbazolyl)benzene] (T_1_ = 2.91 eV, ε = 2.84).^66^ The different hosting properties of mCP and DPEPO can have considerable impact on the TADF properties and OLED performance of emissive guest molecules.^68^Figures 3 and S14 show the steady-state and time-resolved emission spectra of 10 wt % **TCz-CF3** and **Cz-CF3** doped mCP films at room temperature and at 80 K. Both these derivatives show stronger TADF contribution to the total emission, in comparison to zeonex or DPEPO films. For **TCz-CF3**, the emission onset was surprisingly red-shifted in mCP with substantially higher PLQY at Φ_PL_ = 54% (Φ = 39% in DPEPO), despite comparable Δ*E*_ST_ in both hosts (0.04 eV in mCP, 0.06 eV in DPEPO). DPEPO is a more polarizable host with substantially higher dielectric constant that normally causes redshifts in the emission of CT molecules.^67^ Similarly, despite a small Δ*E*_ST_ in both mCP (0.08 eV) and DPEPO (0.11 eV), **Cz-CF3** also showed substantially stronger delayed emission and higher Φ_PL_ of 64% in mCP (Figures S12, S14, and S15, Φ_PL_ = 53% in DPEPO). Indeed, further comparison between **TCz-CF3** and **Cz-CF3** in mCP revealed a striking similarity in the decays, *k*_rISC_, and PLQY. It was not initially clear why two emitters which have such different properties in some media (especially in dilute toluene, see below) could have either very similar or very different photophysical properties depending on the host material used. It is also noted that **TCz-Me**, with a relatively large Δ*E*_ST_ of 0.25 eV, still exhibited moderate TADF intensity in 10 wt % mCP films (Figure S16) and stronger TADF contribution compared to the similar DPEPO and zeonex films. Nonetheless, all further optical studies were focused on the more promising **TCz-CF3** and **Cz-CF3**, which were also structurally appropriate for highlighting any sulfur-induced heavy-atom effects in the optical properties of the materials.

To gain a deeper insight into these emitters we additionally studied the optical properties of **Cz-CF3** and **TCz-CF3** doped in host molecules of varying structural and electronic properties.^61^ First, the small-molecule rigid host UGH-3 [1,3-bis(triphenylsilyl)benzene] (T_1_ = 3.50 eV)^69^ was chosen due to its low dielectric constant, as compared to DPEPO (which consists of highly polar O–C and O=P bonds).^70,71^ A substantially blue-shifted emission onset was observed in the time-resolved spectra of 10 wt % **TCz-CF3** in UGH-3 as compared to the corresponding mCP or DPEPO films (Figure S17), which is surprising considering that UGH-3′s ground state permanent dipole moment is likely to be similar to mCP (similar monomer PL onsets in these two hosts would have been expected).^69^ A highly red-shifted emission band was also seen at 600 nm in the **TCz-CF3**—UGH-3 films, present both in the PF and DF regimes. The high-energy band is attributed to a mixture of locally excited and weakly charge-transfer fluorescence confirmed by its short-lived nature (Figures S17a and S17d). The low-energy longer-lived band on the other hand is assigned to aggregates of **TCz-CF3**, which is consistent with its resemblance to the neat-film time-resolved emission spectra (Figure S17b). This band appears to quench the higher energy monomer TADF. In the case of 10 wt % **Cz-CF3** UGH-3 films, there is a similar and higher-energy PF, followed by a longer wavelength aggregate emission at 540 nm (Figure S17c). This is accompanied by a strong CT-dispersion effect at longer time delays. It is also notable that both **Cz-CF3** and **TCz-CF3** show considerably weaker DF in UGH-3 host compared to in mCP host.

Noting the surprising contrast between the optical properties of films using mCP and UGH-3 hosts, other carbazole-based host molecules were also investigated: namely 10 wt % doped films of both **TCz-CF3** and **Cz-CF3** in 3,3′-di(9*H*-carbazol-9-yl)-1,1′-biphenyl (mCBP), 3,3′-di(carbazol-9-yl)-5-cyano-1,1′-biphenyl (mCBPCN) and 4,4′-bis(9-carbazolyl)-1,1′-biphenyl (CBP). The molecular structures of these hosts are given in Figure S18e. Time-resolved emission profiles and decays for 10 wt % doped films of **TCz-CF3** and **Cz-CF3** in all the carbazole-based hosts were recorded (Figures S19 and S20). A relatively small CT-dispersion and a substantially stronger DF contribution is observed for all these carbazole-containing hosts when compared to DPEPO or UGH-3, and the decays of both emitters are very similar to each other when dispersed in the same host. It is also worth noting that both **Cz-CF3** and **TCz-CF3** show similar red-shifted fluorescence onsets in their steady-state emission when doped in any of the carbazole-based hosts, as compared to DPEPO host (Figure S18).

**Figure 4:**
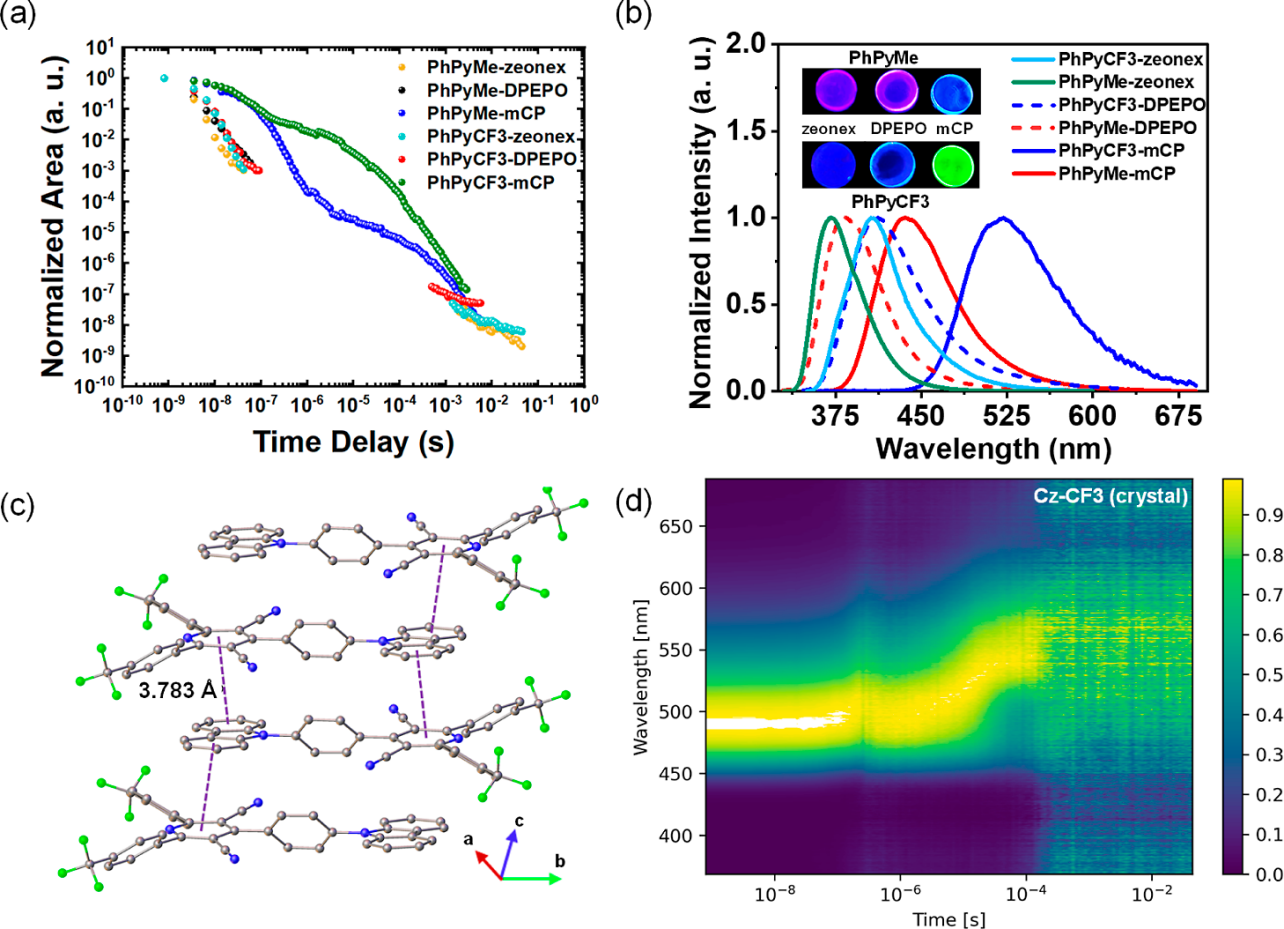
(a) Time-resolved emission decay and (b) steady-state emission of the model compounds **PhPyCF3** and **PhPyMe** in doped solid films (10 wt % for DPEPO and mCP and 1 wt % for zeonex) at room temperature. λ_exc_ = 355 nm. Inset (b) shows doped films with clear, red-shifted emission in mCP as compared to other hosts when excited under 365 nm UV-lamp. (c) X-ray crystal structure of **Cz-CF3** showing the π–π stacking distance between the donor (carbazole) and acceptor (cyanopyridine) units. (d) Contour plots of normalized time-resolved emission spectra of **Cz-CF3** in crystalline state at room temperature.

Considering the above observations collectively, we speculated that exciplex formation between the emitters and the host carbazole units was contributing to the emissive properties of these films. This became dominant over any anticipated heavy-atom effects. To confirm this, the optical properties of the “acceptor-only” versions of **TCz-CF3** and **TCz-Me**, namely **PhPyCF3** and **PhPyMe**, were measured in solid films (Figures 4, S21, S23). Figures 4 and S21 show the steady-state and time-resolved emission profile of these acceptors in 1 wt % zeonex, 10 wt % mCP, and 10 wt % DPEPO films. In the steady-state spectra a large red-shift is observed for both **PhPyCF3** and **PhPyMe** in mCP when compared with DPEPO, and similar to the corresponding D–A materials (Figure 4b). Moreover, the time-resolved emission clearly shows a very strong DF contribution exclusively when doped in mCP—which cannot arise from intramolecular TADF in these donor-free materials. Indeed, no DF was observed (although weak room temperature phosphorescence was seen) for the acceptor fragments **PhPyCF3** and **PhPyMe** doped in zeonex or DPEPO film (Figure S21). This observation establishes guest–host exciplex formation as the dominant process that leads to TADF in films featuring carbazole-based hosts and explains the seemingly contradictory properties previously observed for **TCz-CF3** and **Cz-CF3**. It is worth noting that **PhPyCF3** exhibits significantly increased DF compared to **PhPyMe** in mCP-doped films; this is likely due to the larger π-surface and enhanced electron affinity of the (trifluoromethyl)phenyl side groups, facilitating Coulombic interactions with the electron-rich mCP hosts. The potential for exciplex formation is also seen in the single-crystal X-ray structure of **Cz-CF3** which reveals a face-to-face alignment of Cz and PyCF3 units on different molecules (with a π–π distance of 3.783 Å) which results in a strong TADF in the crystalline state (Figures 4c,d, S4 and S23).

**Figure 5:**
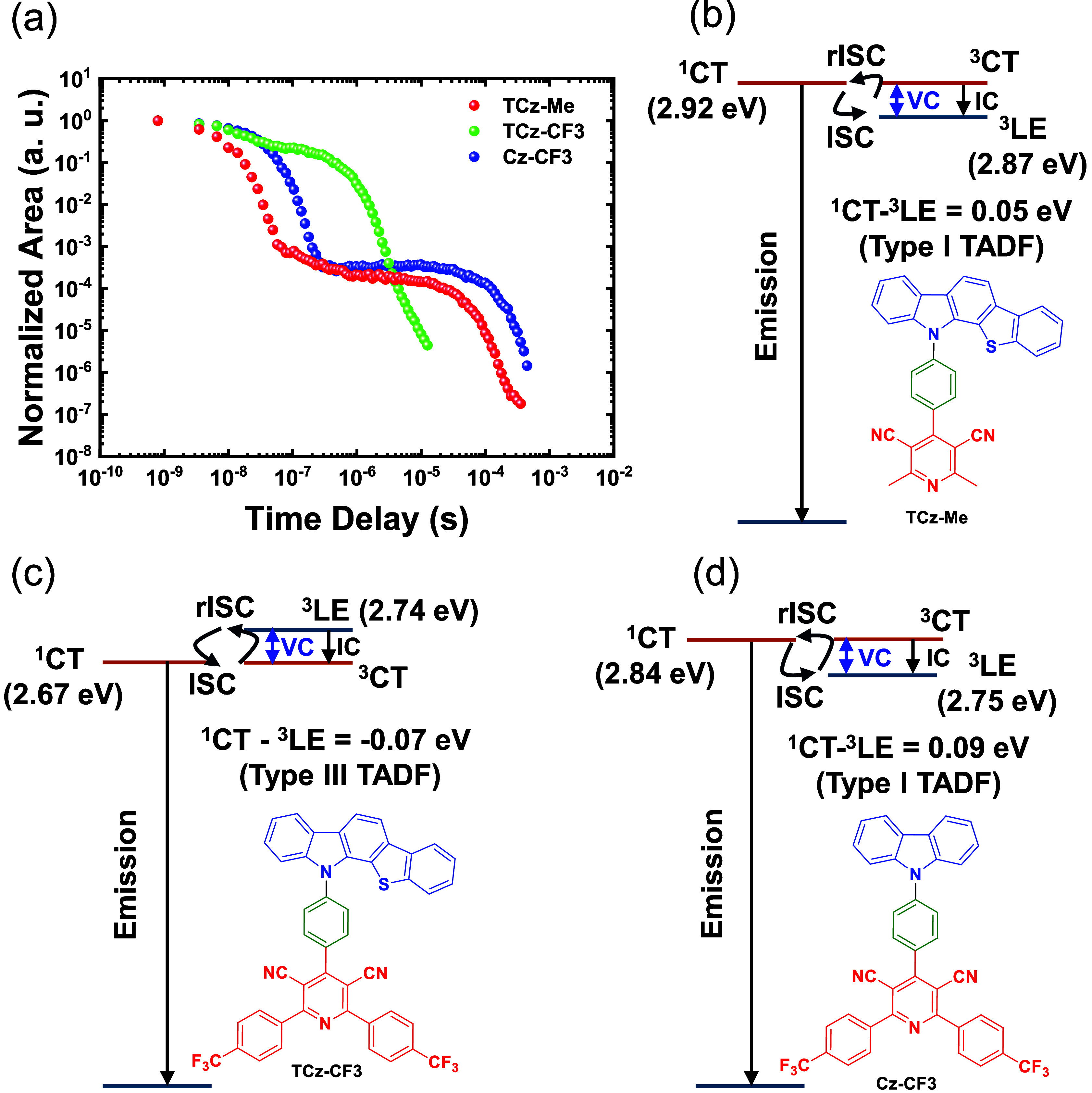
(a) Time-resolved emission decays of the three derivatives studied in this work in degassed toluene solution. λ_exc_ = 355 nm. The proposed model for (b) **TCz-Me**, (c) **TCz-CF3** and (d) **Cz-CF3** in toluene, where ^3^LE state is assumed to be the same as the phosphorescence (T_1_) onset energy measured in their corresponding 1 wt % zeonex films. VC = vibronic coupling, IC = internal conversion.

To explore how host–guest interactions influence the triplet recycling in the discussed derivatives, optical studies were conducted in dilute, degassed toluene solution, expecting monomolecular triplet recycling. To our surprise, a strong TADF was observed for **TCz-CF3** with very fast *k*_rISC_(72) reaching 4.7 ± 0.167 × 10^6^ s^–1^ in comparison to **Cz-CF3** (*k*_rISC_ = 2.4 ± 0.109 × 10^4^ s^–1^) (Figures 3b, 5, S24 and Table S5), contrary to the solid-state emission properties. It is worth noting that despite a similar Δ*E*_ST_ between **Cz-CF3** and **TCz-Me**, the observed *k*_rISC_ and DF/PF ratio is less efficient for **TCz-Me**, hinting toward a subtle energy alignment effect that drives the triplet dynamics in these molecules in solution state, rather than a simple “heavy-atom effect” expected to drive the rISC in **TCz-Me**.^17^ A detailed analysis is given in the discussion section where this aspect is reiterated, and is consistent with the theoretical calculations showing the SOCME values do not significantly improve with the presence of sulfur (vide infra).

### Excited State Calculations

The optimized S_1_ excited state geometries of **TCz-Me**, **TCz-CF3**, and **Cz-CF3** at B3LYP/6-31G(d) show the D and A units to be near orthogonal with C–N–C–C and C–C–C–C dihedral angles between 71 and 90° (compared with 50–64° in the ground state) (Table S3). Time-dependent density functional theory (TD-DFT) data on these excited state geometries indicate that the lowest energy S_0_ ← S_1_ transition is intramolecular CT (^1^CT) in all systems, as observed experimentally in solution (with large solvatochromic shifts) and in zeonex, as discussed above.

The state-specific corrected linear response polarizable continuum solvation model (cLR-PCM)^73,74^ was then applied to optimized S_1_ geometries with argon, toluene and dichloromethane (DCM) as solvents (Table 1). The cLR-PCM S_1_ (^1^CT) energies align with known polarities and dielectric constants of the solvents. The solvatochromic strength increases from **TCz-CF3** (with energy shift between argon and DCM at 0.42 eV) then **TCz-Me** (0.44 eV) to **Cz-CF3** (0.66 eV) in accordance with the shift differences of 0.43, 0.61, and 0.79 eV between the experimental emission maxima in nonpolar methylcyclohexane and polar dichloromethane (Figure S8).

**Table 1:** Experimental Values of Relative Polarities and Dielectric Constants of Solvents, and Computed Excited State Energies (S_1_ and T_1_) from TD-DFT on Optimized S_1_ Geometries of **TCz-Me**, **TCz-CF3**, and **Cz-CF3** Using the cLR-PCM Method with the Corresponding Solvent

solvent	relative polarity index	dielectric constant ε	**TCz-Me**			**TCz-CF3**			**Cz-CF3**		
			S_1_ eV	T_1_ eV	S_1_–T_1_ eV	S_1_ eV	T_1_ eV	S_1_–T_1_ eV	S_1_ eV	T_1_ eV	S_1_–T_1_ eV
argon	0.0	1.43	3.21	2.73	0.48	2.55	2.34	0.21	2.91	2.27	0.64
toluene	2.4	2.57	3.03	2.74	0.29	2.42	2.35	0.07	2.62	2.27	0.35
DCM	3.1	8.93	2.77	2.74	0.03	2.13	2.29	–0.16	2.25	2.25	0.00

**Figure 6:**
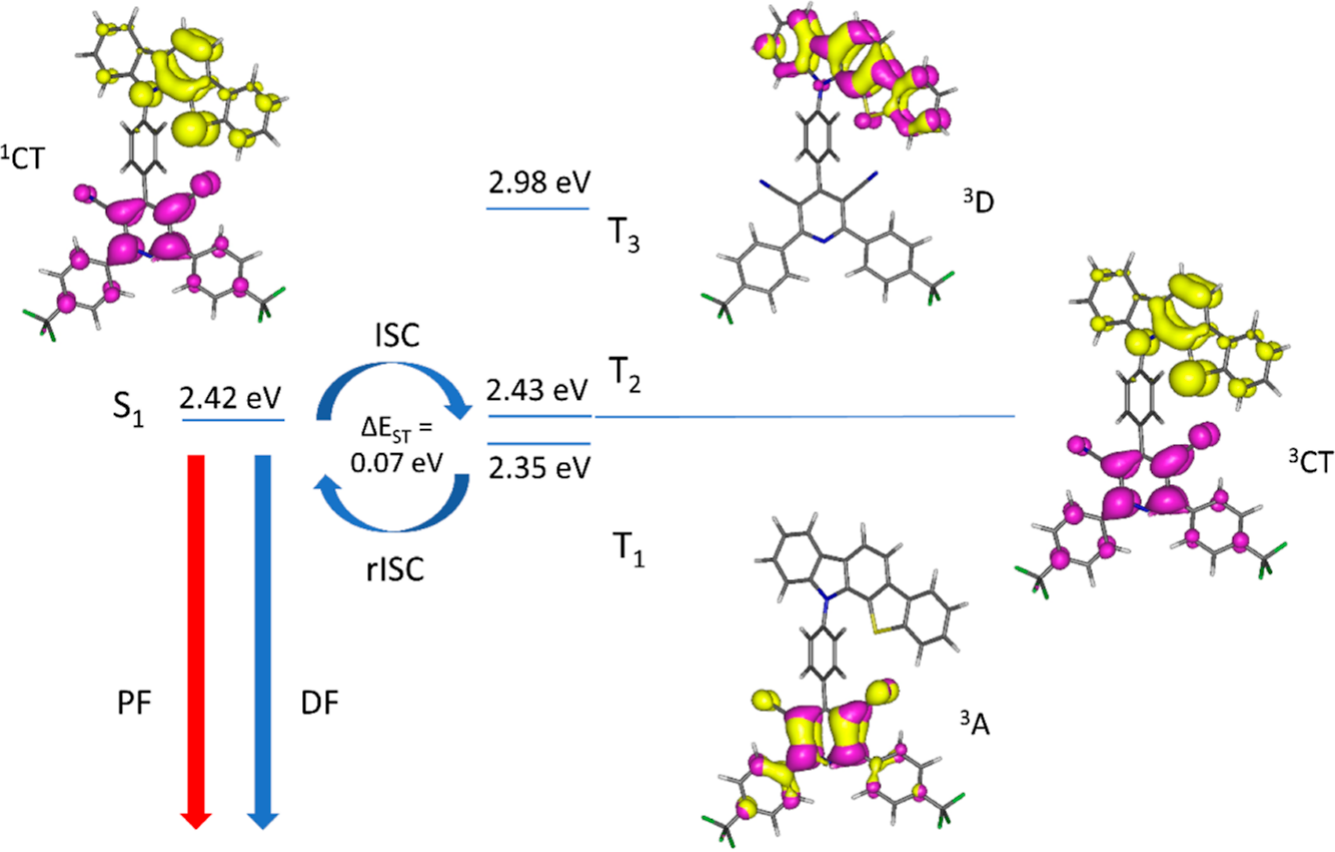
Energy diagram illustrating TADF-active excited states in **TCz-CF3** with NTOs (yellow = hole, purple = particle) for each state, calculated on the optimized S_1_ excited state geometry with toluene solvation model cLR-PCM. Contours in NTOs are drawn at ±0.04 (e/bohr^3^)^1/2^.

Turning to the energy difference between the T_1_ state (local excitation, ^3^LE) and the S_1_ state (charge transfer, ^1^CT), the T_1_ state in argon and toluene is LE in nature, centered at the pyridine acceptor moiety (^3^A) for all compounds. In dichloromethane, the T_1_ state is also ^3^A for **TCz-Me** but is CT (^3^CT) for **TCz-CF3** and **Cz-CF3**. The ISC processes between ^1^CT and ^3^CT states are forbidden. The T_2_ energies of the local excitations in **TCz-CF3** and **Cz-CF3** are 0.22 and 0.03 eV higher in DCM, respectively, compared to their corresponding ^1^CT state energies so the TADF process is likely to be efficient for **Cz-CF3** in DCM, but less so for **TCz-CF3** in DCM (Table S8). The smallest S_1_ – T_1_ energy gaps are found for **TCz-CF3** in toluene at 0.07 eV, **TCz-Me** in DCM at 0.03 eV and **Cz-CF3** in DCM at 0.00 eV (Table 1). The importance of the polarity of the environment with respect to small S_1_–T_1_ energy gaps for efficient TADF processes within each D–A system is demonstrated here, as in previous molecules.^60,75−79^Figures 6, S25 and S26 show the natural transition orbitals (NTOs) of excited states expected to be involved in the TADF process. Table 2 lists the nature and energies of the relevant excited states for the three compounds.

**Table 2:** Energies in eV and Nature of the Excited States from TD-DFT Computations on Optimized S_1_ Geometries of **TCz-Me**, **TCz-CF3** and **Cz-CF3** in Toluene^a^

	T_1_	T_2_	S_1_	T_3_	Δ*E*_ST_	Δ*E*_TT_
**TCz-Me**	2.74 (17) ^3^A	2.91 (0) ^3^D	3.03 (99) ^1^CT	3.02 (99) ^3^CT	0.29	0.17
**TCz-CF3**	2.35 (0) ^3^A	2.43 (99) ^3^CT	2.42 (99) ^1^CT	2.98 (9) ^3^D	0.07	0.08
**Cz-CF3**	2.27 (2) ^3^A	2.61 (97) ^3^CT	2.62 (99) ^1^CT	3.06 (13) ^3^A	0.35	0.34

a % CT values are listed in parentheses. Δ*E*_ST_ = S_1_ energy – T_1_ energy, and Δ*E*_TT_ = T_2_ energy – T_1_ energy.

The singlet states (S_1_) in **Cz-CF3**, **TCz-Me**, and **TCz-CF3** from the calculated NTOs have CT characters (99%), with virtually zero orbital overlap between the hole and particle orbitals which are spatially well separated by the (near)-orthogonally oriented *para*-phenylene bridges. The lowest energy triplet states, T_1_ and T_2_, in all compounds are a local acceptor state (^3^A) and a CT state (^3^CT) respectively, or vice versa. The local triplet states (^3^A) with energies close to the S_1_ CT states facilitate TADF as allowed SOCs occur between singlet and triplet states of different orbital characters with SOC matrix elements (SOCME) calculated at 0.05–0.11 cm^–1^ (Table S9). The forbidden SOCs between a singlet CT state and a triplet CT state have SOCMEs calculated at 0.02–0.04 cm^–1^. The vibronic couplings between close low-energy triplet states (Δ*E*_TT_ 0.08–0.34 eV in toluene, Table 2) are also assumed to greatly enhance the rISC process for TADF with only 0.08 eV gap for **TCz-CF3** in toluene. It is notable that the heavy-atom (sulfur) does not increase the SOCME values here.

Zeonex (solid-state film) is relatively nonpolar and thus predicted to have emission properties closer to toluene and argon gas. **Cz-CF3** and **TCz-Me** in zeonex may not have efficient TADF due to the larger predicted S_1_(^1^CT) and T_1_(^3^LE) energy gaps of 0.64 to 0.29 eV for **Cz-CF3** and **TCz-Me** in argon and toluene (Tables S6 and S7). **TCz-CF3** in zeonex is expected to have efficient TADF with smaller gaps of 0.07 eV (toluene) and 0.21 eV (argon). These predictions are in agreement with experimental observations of weak TADF for **Cz-CF3** and **TCz-Me** and strong TADF for **TCz-CF3** in zeonex.

A T_1_ geometry similar to a S_1_ geometry would strongly facilitate ISC/rISC. The ISC/rISC reorganization energies are predicted from optimized S_1_ and T_1_ geometries in the gas phase at B3LYP/6-31G(d) to be 0.06, 0.004, and 0.17 eV for **TCz-Me**, **TCz-CF3**, and **Cz-CF3**, respectively. The low reorganization energy of 0.004 eV for **TCz-CF3** suggests very similar S_1_ and T_1_ geometries (Figure S27) and thus fast ISC/rISC processes occur, as found experimentally for **TCz-CF3** in toluene and zeonex.

The optimized S_1_ excited state geometries for the acceptor compounds **PhPyMe** and **PhPyCF3** are notably different (Table S3). **PhPyCF3** adopts the near-orthogonal orientation between the pyridyl group and the *para*-phenylphenylene group with a torsion angle of 85°, whereas **PhPyMe** is little changed from the nonorthogonal S_0_ geometry with the corresponding torsion angle of 51°. The nature of the S_0_ ← S_1_ emission is LE (^1^LE, 9% CT) on the pyridine for **PhPyMe** and CT (^1^CT, 98% CT) for **PhPyCF3** (Tables S7 and S8). The observed emission spectra in solvents of different polarities for **PhPyMe** and **PhPyCF3** confirm weak (0.34 eV between methylcyclohexane and dichloromethane emission maxima) and medium (0.56 eV) solvatochromism, respectively (Figure S22). The much lower calculated T_1_ energies compared to calculated S_1_ energies rule out TADF in these acceptor molecules (Tables S6 and S7).

**Figure 7:**
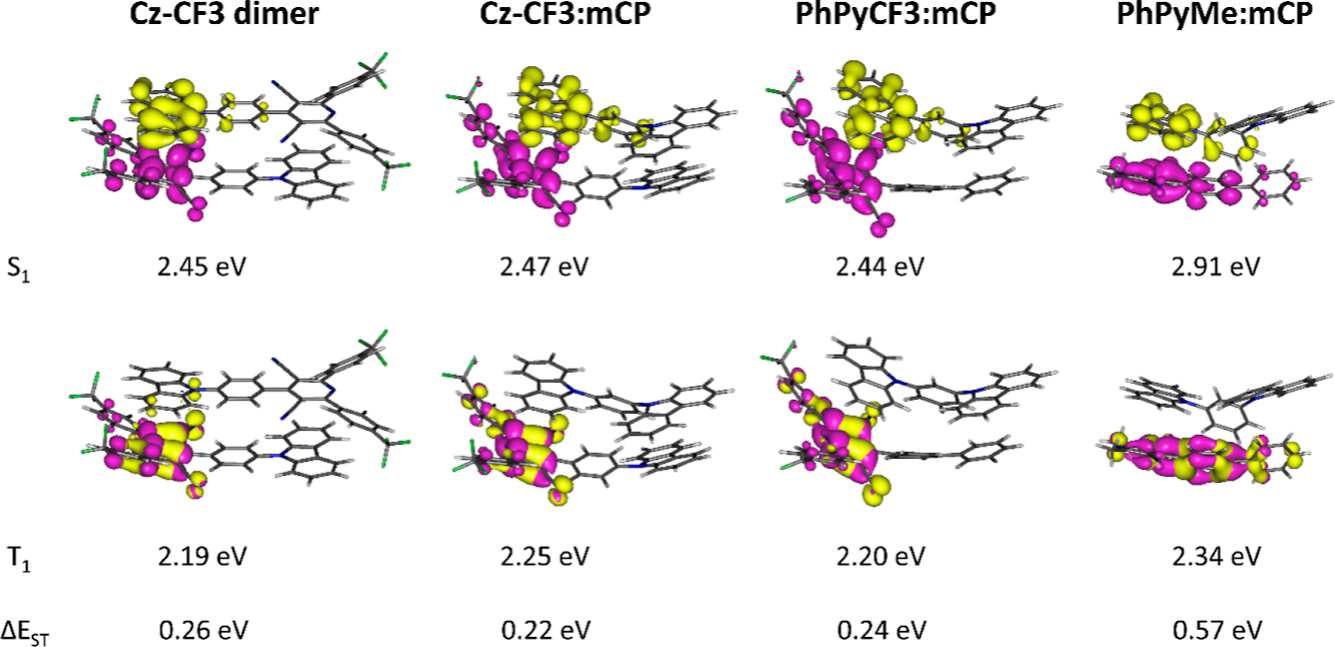
NTOs and energies for the lowest excited singlet and triplet states of optimized S_1_ excited state geometries for **Cz-CF3 dimer**, **Cz-CF3:mCP**, **PhPyCF3:mCP** and **PhPyMe:mCP** with toluene solvation model cLR-PCM. Smaller Δ*E*_ST_ gaps are predicted with DCM as solvent (Table S8).

While computations support the observed emission data for the five compounds in solutions and in zeonex by modeling the systems as discrete molecules, it is shown experimentally here that intermolecular CT is responsible for the observed emissions from all five compounds with mCP as host and the crystal form of **Cz-CF3** (Figure S23b). Previous TD-DFT studies on intermolecular CT from TADF exciplexes relied on optimized S_0_ ground state geometries of interacting molecules.^80−84^ Here, optimized S_1_ excited state geometries on two interacting molecules were carried out instead to predict intermolecular or intramolecular emissions and likely TADF from small S_1_–T_1_ energy gaps. TD-DFT data on the optimized S_1_ geometries of the four molecule pairs, **Cz-CF3 dimer**, **Cz-CF3:mCP**, **PhPyCF3:mCP**, and **PhPyMe/mCP**, reveal singlet intermolecular CT emissions ^1^CT* (the asterisk * is used here to define intermolecular) between the pyridyl group acceptor and the carbazolyl group donor in all cases (Figure 7) at S_1_ states. The environmental polarizabilities in mCP hosts and crystal states are not known, so solvation models using toluene and dichloromethane are applied here as examples. The intermolecular CT S_1_ and local acceptor T_1_ states are similar in energies across the three pairs with CF3, acceptors in agreement with the observed TADF emission data (Figure 3). The **PhPyMe:mCP** pair also has an intermolecular CT S_1_ state but has a larger S_1_–T_1_ gap than the CF3 pairs, thus TADF in the former pair is weaker than the latter pairs, as observed experimentally. The **TCz-CF3:mCP** pair gives nearly identical S_1_(^1^CT*), T_1_(^3^A), and T_2_(^3^CT*) energies and NTOs as those for the **Cz-CF3:mCP** pair which is in accord with nearly identical emissions observed for **TCz-CF3** and **Cz-CF3** in mCP hosts (Figures S28, S29 and Tables S7 and S8). Based on these pair minima locations, the thienocarbazolyl and carbazolyl groups of the D–A systems (**TCz-CF3** and **Cz-CF3**) are not involved in the key emission states responsible for the TADF emissions when in mCP hosts.

### OLED Performance

**Figure 8:**
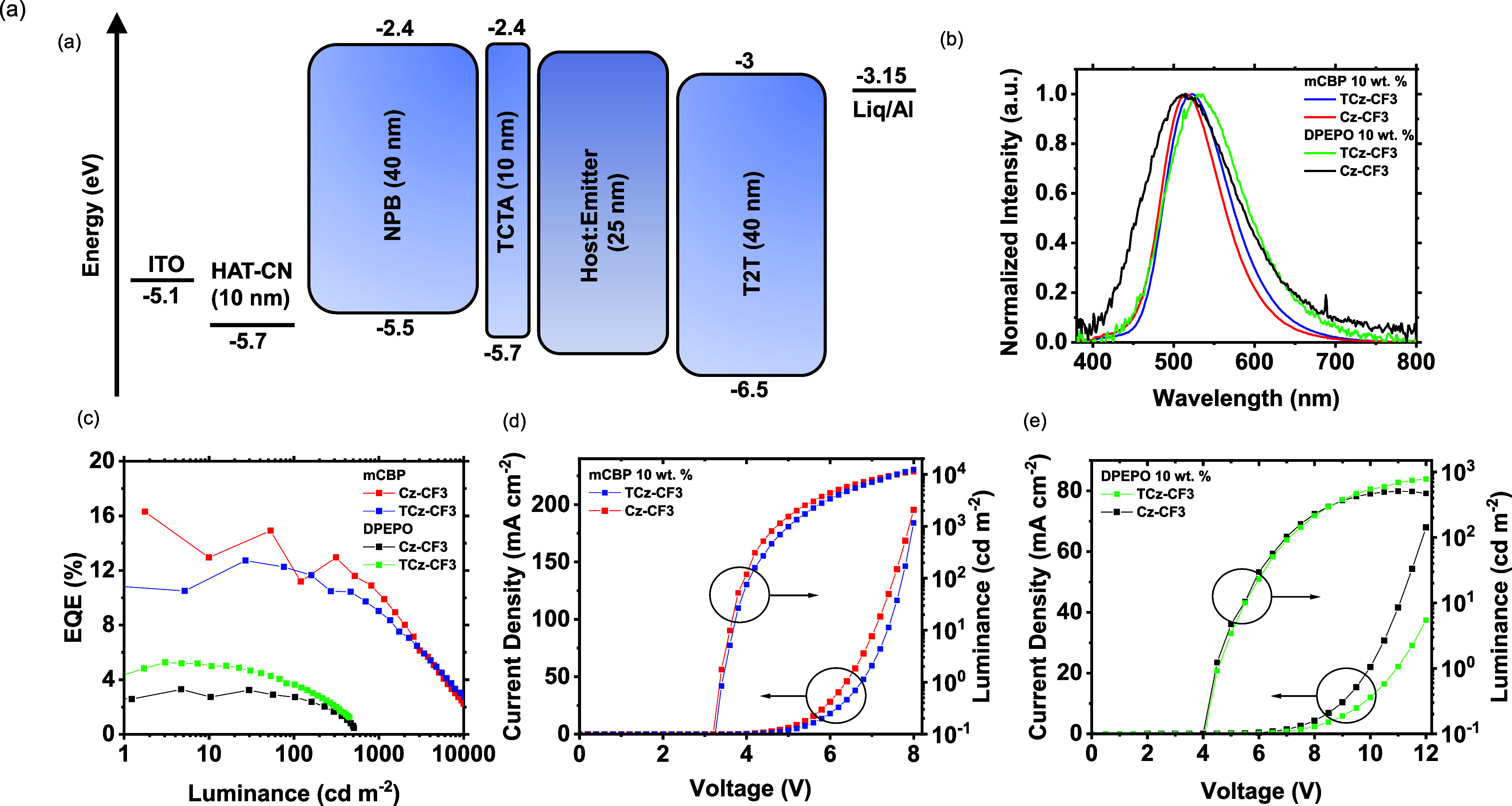
OLED performances of **TCz-CF3** and **Cz-CF3** in different hosts: (a) device structure and (b) EL spectra (collected at 8 V); (c) EQE at different luminances; device J–V–L responses using (d) 10 wt % doping in mCBP host and (e) 10 wt % doping in DPEPO host.

The electroluminescence (EL) properties of both **TCz-CF3** and **Cz-CF3** were investigated in both exciplex-forming and inert hosts. The OLEDs were fabricated using the structure of indium tin oxide (ITO) (anode)|HAT-CN (HIL, 10 nm)|NPB (HTL, 40 nm)|TCTA (electron-blocking layer (EBL), 10 nm)|emitter/host × wt % emitter [emissive layer (EML), 25 nm]|T2T [hole blocking layer (HBL)/ETL, 40 nm]|Liq (3 nm)|Al (cathode, 100 nm) (Figure 8a). (The abbreviations are defined in the Experimental Methods, OLED Fabrication and Testing section). Variation in **TCz-CF3** emitter doping concentration in the range 7.5–15 wt % in mCBP host did not affect the electroluminescence spectrum. The external quantum efficiency (EQE) appears to be optimal at 10 wt % concentration (Figure S30) thus all further device analysis was carried out at 10 wt % doping ratio. As evident from Figure 8b, the electroluminescence spectrum is significantly red-shifted for **Cz-CF3** in mCBP as compared to in DPEPO, following the photoluminescence spectra (Figure S19e). A similar trend is observed in the electroluminescence spectra for **TCz-CF3** OLEDs, with a smaller bathochromic shift in mCBP compared to that of DPEPO. The EQE_max_ is significantly higher for both **TCz-CF3** (12.7% at 1.75 cd/m^2^) and **Cz-CF3** (16.2% eV at 27 cd/m^2^) in mCBP compared to in DPEPO (5.3% at 3 cd/m^2^ and 4.7% at 3 cd/m^2^ for **TCz-CF3** and **Cz-CF3**, respectively) (Figure 8c). The mCBP OLED efficiencies correlate well with the measured PLQY values (Φ_PL_ = 64 and 54% for **Cz-CF3** and **TCz-CF3**, respectively, in mCP, Table S5). The better performance of the **Cz-CF3** devices in mCBP, compared to the **TCz-CF3** devices indicates that the stronger TCz donor unit attached to the **-PyCF3** acceptor might have a detrimental effect on the formed exciplex efficiency (with the mCBP host), in comparison to the weaker carbazole donor in **Cz-CF3**.

The OLEDs in DPEPO have much lower efficiencies, verifying the absence of the previously beneficial exciplex formation. These devices indicate the monomer device efficiency but the data is contradictory to the measured PLQYs (Φ_PL_ = 53 and 39% for **Cz-CF3** and **TCz-CF3**, respectively, Table S5). Presumably, this arises from the weak triplet harvesting efficiency of **Cz-CF3** and **TCz-CF3** in DPEPO (Figure S20), as well as strong homomolecular interactions that quench the monomer TADF efficiency. Nevertheless, the higher TADF contribution of **TCz-CF3** in DPEPO (Figure S20) results in more efficient OLEDs.

Further investigation of the efficient exciplex-driven **Cz-CF3** OLEDs in mCBPCN carbazole-based host is shown in Figure S31. The presence of the cyano group in mCBPCN, compared to mCBP (Figure S18e), is expected to affect the exciplex formation (already observed optically in Figure S20) and to modify the electrical properties of the device. From the J–V–L plot a lower turn-on voltage is observed in the devices in mCBPCN accompanied by a lower luminance. Lower turn-on voltage is explained by the higher electron mobility of the mCBPCN, as compared to the hole transport properties of the mCBP host, leading to a different charge balance in the OLEDs. The lower luminance, together with the blue-shifted spectrum in mCBPCN (Figure S31c) is explained by the weaker exciplex formation, due to the presence of the electron-withdrawing cyano group. This also results in a lower triplet harvesting efficiency and explains the increased efficiency roll-off in the mCBPCN devices (Figure S31a). These observations in devices are therefore largely consistent with the optical properties previously described for both the emitters (vide infra), and further demonstrate that intermolecular exciplex effects (in carbazole-based hosts) are dominant over any “heavy-atom effect” in these materials. OLED data are collated in Table S11.

## Discussion

In this work, we uncovered seemingly contradictory photophysical properties between **TCz-CF3** and **Cz-CF3** when dispersed in various host matrices. In the solution state, **TCz-CF3** outperforms the other derivatives in this study. Theoretical calculations on **TCz-CF3** reveal no significant enhancement in spin–orbit coupling matrix elements, which typically govern *k*_ISC_/*k*_rISC_ rates in such molecules. Our current hypothesis critically shows that the ^3^LE state in **TCz-CF3** is close in energy to the ^1^CT–^3^CT pair, (i.e., both rISC and rIC gaps are very small),^16,74^ but slightly higher (0.07 eV), resulting in a charge-transfer nature for the lowest triplet excited state (a type III TADF system as classified previously) (Figure 5c).^17^ Since phosphorescence from a ^3^CT state is strictly forbidden, such energy alignment enhances the rISC efficiency by blocking a major nonradiative triplet deactivation pathway. It is well-established that ^1^CT and ^3^CT are nearly iso-energetic, and their transition is mediated by a third state, often a locally excited triplet state centered around either the donor or acceptor units. As observed in Figure 5c, **TCz-CF3** forms a special case where the energies of ^1^CT ∼ ^3^CT < ^3^LE.^85^ This is very similar to the case we have previously elucidated for the through-space CT TADF molecule **TpAT-tFFO**, with 9,9-dimethyl-9,10-dihydroacridine as a donor (A), 2,4-diphenyl-1,3,5-triazine as an acceptor (T) and triptycene (Tp) as a bridge connecting the A and T subunits in a tilted face-to-face (tFF) configuration at an optimal (O) spatial separation.^86^ This molecule also has an energy level ordering, ^1^CT ∼ ^3^CT < ^3^LE, and in that case the SOC between ^1^CT and ^3^LE was found to be very large, giving a *k*_rISC_ rate ca. 5 × 10^–6^ s^–1^, just as observed here for **TCz-CF3** in toluene, but was found to be highly sensitive to the magnitude of the energy gaps between the states. In this type III TADF, the SOC between the mediator ^3^LE state and ^1^CT state is very high leading to fast rISC, given small energy gaps between S_1_–T_1_ and T_1_–T_2_ (in the order of 50 meV). Like **TpAT-tFFO**, **TCz-CF3** in toluene hits this perfect energy alignment for highly efficient rISC with an estimated Δ*E*_ST_ of 70 meV. The fact that ^3^CT is below ^3^LE is critical as it prevents a nonradiative decay channel for triplets, greatly enhancing the efficiency of triplet recycling. This is reflected in the solution state photophysics of **TCz-CF3**, where a fast *k*_rISC_ and a large DF/PF ratio are observed (Figure S24 and Table S5). In contrast, **Cz-CF3** and **TCz-Me** both have a lowest excited triplet state that is locally excited in nature, and ISC/rISC transitions are controlled by the singlet–triplet gap between ^1^CT and ^3^LE states (a type I TADF system) (Figure 5b,d).^17^ Despite a relatively smaller gap in **TCz-Me** compared to **Cz-CF3**, heavy-atom effects, exemplified by sulfur, do not significantly contribute to the overall triplet dynamics, as evidenced by the lack of a higher DF intensity with an efficient DF/PF ratio (Figures 5, S24, and Table S5). An in-depth analysis in solid-state doped films emphasizes the critical role played by host–guest interactions, which are often neglected in the literature.^87^ In zeonex-doped films, regardless of the presence of sulfur, it is the lowest excited singlet–triplet gap that determines the overall triplet recycling efficiency (Figure 2). Further investigations with a range of small molecule hosts at higher doping concentrations (10 wt %) directly correlate with OLED efficiencies. Notably, excited state modulations occur when derivatives are doped in electron-rich host molecules based on carbazole with large-π surfaces. In these hosts, exciplex formation dominates the triplet dynamics, bypassing any potential heavy-atom effect. **Cz-CF3** exhibits near-identical *k*_rISC_ and higher photoluminescence quantum efficiency in mCP (a representative carbazole-based host) compared to **TCz-CF3** (Figure 3). This trend extends to other carbazole-based hosts. OLEDs fabricated in the carbazole-based host (10 wt % mCBP) show significantly better device efficiency for **Cz-CF3** compared to **TCz-CF3**, reinforcing the negligible heavy-atom effect offered by sulfur (Figure 8c). However, it is worth mentioning that the better performance of the **Cz-CF3** devices in mCBP, compared to the **TCz-CF3** devices, indicates that the stronger TCz donor unit attached to the -PyCF3 acceptor seems to hinder or prevent exciplex formation (with the mCBP host), in comparison to the weaker carbazole unit in **Cz-CF3**. This observation further suggests the host–guest “exciplex” interaction depends on the strength of the attached donor in the D–A TADF molecules. On the other hand, in DPEPO (a polar host) and UGH-3 (a nonpolar host), both **TCz-CF3** and **Cz-CF3** appear to perform poorly in terms of overall emission efficiency and triplet contribution in their respective emission profiles compared to the carbazole-based hosts. However, significant aggregation in these hosts is envisioned to act as additional ISC/rISC channels, resulting in poor emission intensity and significantly reduced device efficiency (in 10 wt % DPEPO) (Figures 3 and 8c).

## Conclusions

This investigation into a series of donor–acceptor charge-transfer emitters, prepared in the expectation of observing heavy-atom effects from the sulfur atom of the 12*H*-benzo[4,5]thieno[2,3-*a*]carbazole donor, has instead demonstrated that intermolecular host–guest interactions surprisingly dominate the triplet dynamics. Control experiments using the relevant acceptor fragments reinforce our interpretation of exciplex-driven TADF, while intramolecular heavy-atom effects have minimal impact in both experiments and TD-DFT calculations. This study shows unequivocally that heavy-atom effects are not always manifest in sulfur-containing TADF emitters, or can be completely overridden by environmental effects. This work highlights the importance of host–guest interactions, exciplex formation, the role played by the intramolecular donor strength on the ability for the intermolecular interactions to dominate and variable impacts of heavy-atom effects in TADF systems, with profound implications for OLEDs and other applications of TADF molecules.^88−91^

## Experimental Methods

### General Information

Commercial reagents were purchased and used without further purification. Reactions were conducted under an argon atmosphere, unless otherwise stated. Glassware was dried overnight in an oven at 80 °C. Solvents and liquid reagents were added by syringe or cannula, and solid reagents were added under a positive pressure of argon. Degassing was performed by bubbling argon through the reaction mixture using an argon-filled balloon fitted with a syringe needle. Thin layer chromatography (TLC) analysis was performed by using Merck Silica gel 60 F_254_ TLC plates and spots were visualized by UV irradiation at 365 and 254 nm. Column chromatography was performed using silica gel 60 purchased from Fluorochem. ^1^H, ^19^F and ^13^C{^1^H} NMR spectroscopy was carried out on Bruker AV400, Varian VNMRS 600 and 700 spectrometers. Spectra were recorded at 295 K in commercially available deuterated solvents and referenced internally to the residual solvent proton resonances.^92^ Electrospray ionization mass spectra (ESI) were recorded using a Waters Acquity TQD Tandem Quadrupole mass spectrometer. Atmospheric pressure solids analysis probe (ASAP) ionization mass spectra were obtained using an LCT Premier XE mass spectrometer and an Acquity UPLC from Waters Ltd. at 350 °C. High-resolution mass spectrometry was carried out on a Quadrupole time-of-flight (QToF) mass spectrometer. Thermogravimetric analysis (TGA) was carried out on a PerkinElmer Pyris 1 instrument with a nitrogen gas flow of 20 mL min^–1^. Measurements were carried out from 30 to 400 °C with a heating rate of 10 °C min^–1^. Differential scanning calorimetry (DSC) measurements were performed using a PerkinElmer DSC 8500 instrument. Helium gas was used with a flow rate of 20 mL min^–1^ and measurements were conducted from −60 to 300 °C at 10 °C min^–1^. A potentiostat (AutoLab30) interfaced with a computer was used for the electrochemical measurements. A three-electrode cell containing Pt wire pseudoreference electrode, a Pt counter electrode and a Pt working electrode was used under nitrogen. Cyclic voltammograms were obtained at a scan rate of 100 mV s^–1^. For further details see Table S1.

### X-ray Crystallography

The X-ray single crystal data were collected at 120.0(2) K using MoKα radiation (λ = 0.71073 Å) on a Bruker D8Venture (Photon III MM C14 CPAD detector, IμS–III-microsource, focusing mirrors) 3-circle diffractometer equipped with a Cryostream-700 (Oxford Cryosystems) open-flow nitrogen cryostat. Both structures were solved by direct methods and refined by full-matrix least-squares on F^2^ for all data using OLEX2^93^ and SHELXTL^94^ software. All non-hydrogen atoms were refined in anisotropic approximation, hydrogen atoms were placed in the calculated positions and refined in riding mode. Molecule **Cz-CF3** in crystal is located on a 2-fold axis. Crystal data and parameters of refinement are listed in Table S2.

### Computational Details

Geometry optimizations were performed with the Gaussian 16 package.^95^ Gas-phase ground state (S_0_) geometries were fully optimized without symmetry constraints using the hybrid-DFT functional B3LYP^96,97^ with the 6-31(d) basis set.^98,99^ All fully optimized S_0_ geometries were true minima based on no imaginary frequencies found from frequency calculations. Best geometry fittings based on root-mean-square (rms) errors (misfits in angstroms–the lower the value the better the fit) were determined with the OLEX2 package.^93^ The dihedral angles between rings from ring planes and the shortest intermolecular distances between centroids of carbazole and pyridine rings were measured with Mercury software.^100^ Gas phase singlet excited state (S_1_) geometries were optimized using the td opt command whereas gas phase triplet excited (T_1_) geometries were located with the spin as triplet at B3LYP/6-31G(d). The optimized S_0_ geometries were used as starting models for S_1_ and T_1_ geometry optimizations. For geometry optimizations on pairs of molecules where intermolecular interactions are correctly modeled, the Grimme dispersion factor GD3BJ is applied^101^ with the intermolecular distances listed in Table S4. There are many possible conformations involving two molecules to locate as minima but this search is beyond the scope of this study. The antiparallel planar–planar pattern observed in the X-ray structure of **Cz-CF3** is the basis used for locating the minima of pairs here and thus these antiparallel planar-to-planar motifs are considered as appropriate conformations in this study. The popular B3LYP functional [and indeed many other pure/hybrid DFT methods with zero/low Hartree–Fock (HF) wave contributions] is known to significantly underestimate CT energies with respect to LE energies.^102^ The Coulomb-attenuating method, CAM-B3LYP,^103^ addresses this discrepancy and has been employed in many computational studies investigating the CT energies of donor–acceptor molecules.^104−106^ The larger HF contribution in CAM-B3LYP means that all computed transition energies are generally overestimated in TD-DFT calculations at CAM-B3LYP.^107^ The parameter μ in CAM-B3LYP determines the balance of DFT to HF exchange at the intermediate point in the long-range exchange interaction.^103^ If μ = 0, the long-range-corrected (LC) DFT calculation corresponds to the pure (non-LC) DFT calculation, and conversely μ = ∞ corresponds to the standard HF calculation.^108^ The parameter μ in CAM-B3LYP is 0.33 and, to lower the HF contribution, this parameter is adjusted to 0.27 for TD-DFT computations here and elsewhere for direct comparison with experimental emission data.^79,109−115^

The CAM-B3LYP functional has been successfully applied to TADF molecules elsewhere.^116,117^ The six lowest singlet and six lowest triplet transitions were predicted from TD-DFT on the optimized S_1_ geometries with the state-specific corrected linear response polarization continuum model (cLR-PCM).^73,74^ NTO (NTO) calculations were performed on the optimized S_1_ geometries to visualize the hole and particle orbitals.

The compound mCP [1,3-bis(*N*-carbazolyl)benzene] is a common OLED-compatible host with an experimental high triplet energy (T_1_) of 2.91 eV. This value is in excellent agreement with the T_1_ value of 2.89 eV and the nature of the T_1_ NTO is LE at the *meta*-phenylene moiety from calculations here (Figure S28). The NTO figures were generated using the Gabedit package.^118^ The % CT values were derived by (i) defining the atoms for donor and acceptor units, (ii) calculating the % donor and % acceptor values in each molecular orbital using electronic structure calculations and (iii) calculating the % CT from NTO orbitals using the TD-DFT generated data and the % donor and % acceptor values with GaussSum software.^119^ Spin–orbit coupling matrix elements (SOCME) were obtained from TD-DFT computations at CAM-B3LYP with def2/J TZVP as the basis set and for the six lowest singlet and six lowest triplet transitions using the Orca package.^120^

### Photophysical Characterization

Absorption spectra for all solutions were collected using a double beam Shimadzu UV-3600 UV/vis/NIR spectrophotometer and by using a 1 cm quartz cuvette. Steady-state photoluminescence spectra were measured using Jobin-Yvon Fluorolog spectrophotometers. Time-resolved measurements were detected by a spectrograph and a gated iCCD camera (Stanford Computer Optics 4Picos ICCD camera), where samples were excited with a Nd:YAG laser (EKSPLA) emitting at 355 nm, with a repetition rate of 10 Hz. These measurements were performed either under vacuum at room temperature or at 20 K (custom coldfinger cryostat with He compressor) or at 80 K under a stream of dry temperature-controlled nitrogen gas (JanisVNF-100cryostat). Photoluminescence quantum yields (PLQYs) were measured using a calibrated Quantaφ integrating sphere with coupled Jobin Yvon FluoroLog-spectrometer with PMT detector (0.5 s integration time) and analyzed using FluorEsscence software. The sphere was flushed with N_2_ for 30 min prior to measurement to prevent triplet quenching by atmospheric oxygen, and the excitation wavelength for PLQYs was 330 nm with 5 nm bandpass. Solutions (in methylcyclohexane (MCH), toluene (PhMe), and dichloromethane (DCM)) of all the studied samples for photophysical characterization were prepared at low concentration of 50 μM for measurements to strictly prevent intermolecular interactions. Degassed solutions were obtained by 5 freeze–pump–thaw cycles to remove all dissolved oxygen. Solid state samples were fabricated by drop casting onto quartz. To prepare the 10 wt % doped films of emitters in a host matrix, 90% w/w (0.9 mg) of the host was dissolved in 0.1 mL of solvent and to this was added 10% w/w (0.1 mg) of the emitter.

### OLED Fabrication and Testing

OLEDs were fabricated on patterned ITO coated glass (VisionTek Systems) with a sheet resistance of 15 Ω/sq. Oxygen-plasma-cleaned substrates were loaded into a Kurt J. Lesker Super Spectros deposition chamber, and both the small molecule and cathode layers were thermally evaporated at a pressure of below 10^–7^ mbar. The materials used for the transport and blocking layers were, 1,4,5,8,9,11-hexaazatriphenylenehexacarbonitrile (HAT-CN) as the hole injection layer, *N*,*N*-bis(naphthalen-1-yl)-*N*,*N-*bis(phenyl)benzidine (NPB) as the hole injection/transport layer (HIL/HTL), 3,3′-di(9*H*-carbazol-9-yl)-1,1′-biphenyl (mCBP) as the EBL, the EML had mCBP or bis[2-(diphenylphosphino)phenyl] ether oxide (DPEPO) as a host doped with the TADF emitters, 2,4,6-tris(biphenyl-3-yl)-1,3,5-triazine (T2T) as the HBL, T2T and 8-hydroxyquinolinolato-lithium (Liq) as the electron transport/injection layer (ETL/EIL), and an aluminum (Al) cathode. NPB, mCP, and T2T were purchased from Sigma-Aldrich and sublimed before use. Freshly evaporated devices were transferred into either a calibrated 6 in. integrating sphere in a glovebox or a calibrated 10 in. sphere under ambient conditions. Electrical properties were measured using a sourcemeter (Keithley 2400) simultaneously with emission spectrum and intensity with a calibrated fiber-coupled spectrometer (Oceanoptics USB 4000). In the 6 in. sphere, an additional silicon photodiode was used to monitor very low luminance. All devices were evaluated at 293 K.

## Supporting Information Available

Synthetic details; NMR and mass spectra; CV and TGA traces; additional absorption and emission spectra, computational data and OLED data (PDF)
The CIF file for **Cz-CF3** have been deposited with the Cambridge Structural Database, CCDC 2320840 (CIF)
The CIF file for and **PhPyMe** have been deposited with the Cambridge Structural Database, CCDC 2320841 (CIF)

## Supplementary Material

cm4c00850_si_001.pdf

cm4c00850_si_002.cif

cm4c00850_si_003.cif
