# Supplementary material for: Exciplex, Not Heavy-Atom Effect, Controls the Triplet Dynamics of a Series of Sulfur-Containing Thermally Activated Delayed Fluorescence Molecules
Source: Chem Mater. 2024 Aug 2;36(15):7135–50. doi: 10.1021/acs.chemmater.4c00850 (PMC11325549; doi:10.1021/acs.chemmater.4c00850)
Supplement: Supplementary file 1 — cm4c00850_si_001.pdf [file cm4c00850_si_001.pdf]

Supporting Information

Exciplex, not Heavy-atom Effect, Controls the Triplet Dynamics of a Series of Sulfur-containing Thermally Activated Delayed Fluorescence Molecules

Saliha Öner, Suman Kuila,* Kleitos Stavrou, Andrew Danos, Mark A. Fox, Andrew P. Monkman,* Martin R. Bryce*

Table of Contents **Page**

1. **Synthesis and Characterization of the Compounds** **S2**
2. **Thermogravimetric Analysis (TGA) Measurements** **S9**
3. **Cyclic Voltammetry (CV) Measurements** **S10**
4. **X-Ray Crystallography** **S12**
5. **Ground-State Calculations** **S14**
6. **Optical Properties** **S17**
7. **Excited-State Calculations** **S26**
8. **Electroluminescence Properties** **S30**
9. **Characterizations** **S31**
   9.1 NMR Spectra **S32**
   9.2 Mass Spectra **S46**
10. **Cartesian Coordinates** **S47**
11. **References** **S51**

1. Synthesis and Characterization of the Compounds

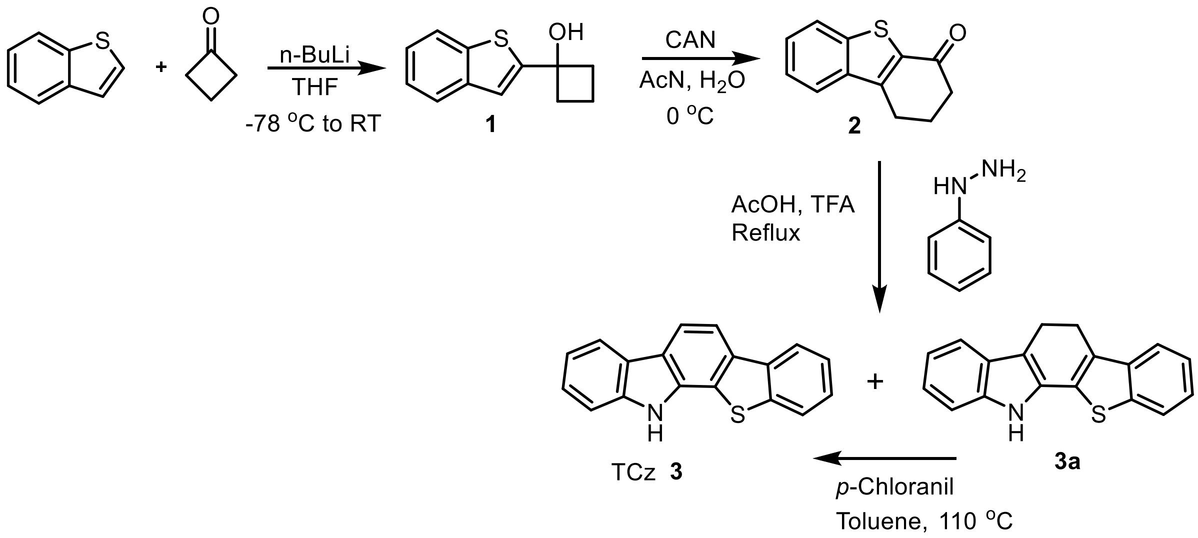

Scheme S1. Synthesis of TCz 3

Synthesis of 1-(benzo[*b*]thiophen-2′-yl)cyclobutanol (1)

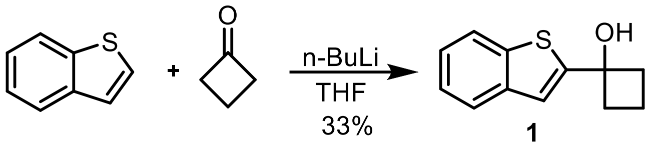

An oven-dried Schleck flask was degassed under vacuum and backfilled with argon, 3-times. Benzo[*b*]thiophene (500 mg, 3.73 mmol) and dry THF (10 mL) were transferred to the flask and the solution was cooled to -78 °C by using a dry ice/acetone bath. *n*-Butyllithium (*n*-BuLi, 1.6 M in hexane, 2.6 mL, 4.1 mmol) was added dropwise and the resultant solution was stirred at -78 °C for 1 h. Afterwards, cyclobutanone (0.33 mL, 4.48 mmol) was added dropwise and the resultant solution was stirred at RT for overnight. Saturated NH_4Cl (10 mL) was added and the THF phase was separated. The water phase was washed with THF (15 mL) and EtOAc (15 mL). The organic phases were combined, dried over $MgSO_4$ and concentrated. The crude product was purified by column chromatography (silica gel, hexane/EtOAc, 2/1, v/v) and product was obtained as a white solid. Yield = 0.25 g (33%). R_f = 0.37 (hexane/EtOAc, 2/1, v/v). ^{1}H NMR (400 MHz, $CDCl_3$) δ 7.81 (ddt, J = 7.5, 1.5, 0.8 Hz, 1H), 7.75 – 7.71 (m, 1H), 7.38 – 7.28 (m, 2H), 7.27 (s, 1H), 2.67 – 2.57 (m, 2H), 2.53 – 2.43 (m, 2H), 2.41 (s, 1H), 2.07 – 1.93 (m, 1H), 1.82 (dp, J = 11.4, 8.6 Hz, 1H). ^{13}C{^{1}H} NMR (101 MHz, $CDCl_3$) δ 152.07, 139.68, 139.65, 124.33, 124.22, 123.52, 122.48, 119.16, 75.19, 38.18, 12.84. Characterization is in agreement with that reported in the literature.[1]

Synthesis of 2,3-dihydrodibenzo[*b*,*d*]thiophen-4(1*H*)-one (2)

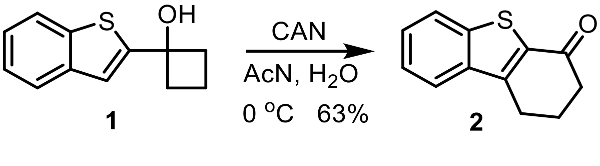

Compound **1** (0.627 g, 3.07 mmol) was dissolved in acetonitrile (AcN) (15 mL) and H_2O (15 mL) was added. The solution was cooled to 0 °C by using an ice bath. Ammonium cerium(IV) nitrate (CAN,

4.21 g, 7.68 mmol) was added in a few portions. The solution color turned to yellowish. The solution was stirred at 0 °C for about 45 min. Afterwards, saturated sodium thiosulfate ($Na_2S_2O_3$) (38 mL) was added to quench the reaction. The organic phase was extracted with EtOAc (30 mL x 3), washed with brine (30 mL), dried over $MgSO_4$ and concentrated. A brown solid was obtained and was purified by column chromatography [silica gel, petroleum ether (PE) /EtOAc, 5/1, v/v)]. The 2nd yellow fraction was the product, **2** which gave a yellowish solid after concentration. Yield = 0.39 g (63%). R_f = 0.23 (PE/EtOAc, 5/1, v/v). ^{1}H NMR (400 MHz, $CDCl_3$) δ 7.88 (dt, *J* = 8.1, 1.0 Hz, 1H), 7.82 (ddd, *J* = 7.9, 1.4, 0.7 Hz, 1H), 7.49 (ddd, *J* = 8.1, 7.1, 1.4 Hz, 1H), 7.43 (ddd, *J* = 8.2, 7.1, 1.2 Hz, 1H), 3.07 (t, *J* = 6.1 Hz, 2H), 2.77 – 2.70 (m, 2H), 2.37 – 2.27 (m, 2H). ^{13}C{^{1}H} NMR (101 MHz, $CDCl_3$) δ 193.77, 147.96, 142.53, 138.26, 136.23, 128.09, 124.76, 123.88, 123.54, 38.61, 24.10, 23.97. Characterization is in agreement with that reported in the literature.[1]

Synthesis of 12*H*-benzo[4,5]thieno[2,3-*a*]carbazole (TCz, 3)

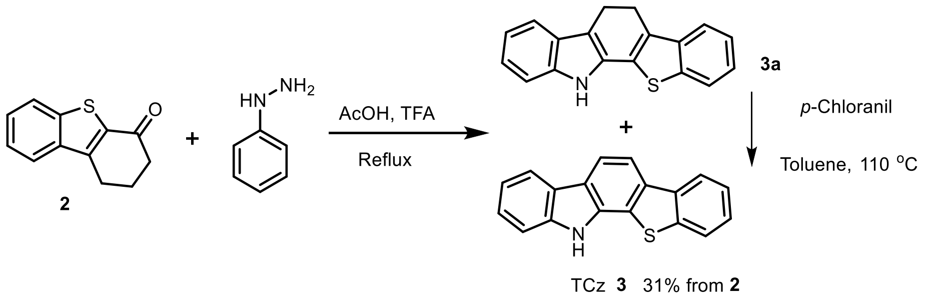

To a round-bottom 2-necked flask equipped with a reflux condenser was added **2** (0.78 g, 3.8 mmol) and phenylhydrazine (0.42 g, 3.8 mmol). AcOH (12 mL) and TFA (4 mL) were added and the resulting solution was refluxed for overnight. After cooling to RT, water (40 mL) was added and the yellow precipitate was filtered, washed with water (200 mL) and dried overnight in air. Column chromatography was performed to purify the compound [silica gel, petroleum ether (PE) /THF, 6/1, v/v)]. The first eluted product was identified as non-aromatized **3a**; the majority of the crude product was subsequently isolated as a mixture of TCz **3** and **3a** (total 672 mg). This mixture was placed in a 1-necked round-bottomed flask equipped with a reflux condenser and dissolved in toluene (20 mL). To this solution, *p*-chloranil (726 mg, 2.9 mmol, considering 100 % yield in the previous step) was added and the reaction mixture was refluxed for 4 h at 110 °C. Upon completion, the reaction mixture was evaporated to dryness and then dissolved in EtOAc (50 mL), adsorbed onto silica and purified by column chromatography (silica gel, EtOAc/Hex, 3/97 to 1/4, v/v), followed by washing with Et_2O (30 mL) to obtain a greenish-yellow powder of TCz (**3**). Yield = 321 mg (31% overal yield from **2**). R_f = 0.2 (EtOAc/Hex, 3/97 to 1/4, v/v). ^{1}H NMR (400 MHz, $CDCl_3$) δ 8.33 (s, 1H), 8.28 – 8.22 (m, 1H), 8.19 – 8.12 (m, 2H), 8.05 (d, *J* = 8.3 Hz, 1H), 7.97 – 7.90 (m, 1H), 7.59 – 7.43 (m, 4H), 7.31 (ddd, *J* = 8.0, 7.1, 1.0 Hz, 1H). ^{13}C{^{1}H} NMR (101 MHz, DMSO-d_6) δ 140.19, 138.68, 136.74, 134.63, 133.87, 126.81, 126.01, 125.36, 123.81, 123.48, 122.41, 121.42, 121.21, 120.80, 119.80, 118.02, 113.45, 111.87. MS (ESI-TOF): m/z [M]$^+$ calcd for $C_{18}H_{11}NS$, 273.061; found, 273.219 (100.00%), 274.207 (70.41%). Characterization of TCz (**3**) is in agreement with that reported in the literature.[1]

3a: ^{1}H NMR (400 MHz, $CDCl_3$) δ 8.12 (s, 1H), 7.84 (dt, J = 8.0, 0.9 Hz, 1H), 7.72 (ddd, J = 7.9, 1.1, 0.7 Hz, 1H), 7.60 – 7.54 (m, 1H), 7.44 – 7.35 (m, 2H), 7.30 (ddd, J = 8.2, 7.1, 1.3 Hz, 1H), 7.22 – 7.12 (m, 2H), 3.16 (s, 4H). MS (ESI-TOF): m/z [M]$^+$ calcd for $C_{18}H_{13}NS$, 275.077; found, 275.233 (100.00%).

Synthesis of 4-(4'-bromophenyl)-2,6-dimethylpyridine-3,5-dicarbonitrile (4)

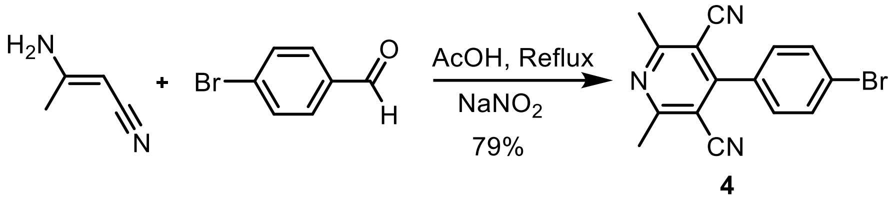

In a 100 mL, 2-necked round bottom flask equipped with a reflux condenser was placed 3-aminocrotononitrile (6.0 g, 73 mmol) and 4-bromobenzaldehyde (5.4 g, 29.2 mmol). Glacial acetic acid (AcOH) (50 mL) was added and the resultant mixture was refluxed overnight. After cooling to 75 °C, sodium nitrite (20 g, 292 mmol) was added portionwise and the mixture was stirred for 1 h at 75 °C. Water (150 mL) was added to quench the reaction and a pale white precipitate was obtained. The precipitate was filtered, washed with water and dried at 60 °C overnight to give **4**. Yield = 7.2 g (79%). R_f = 0.23 (DCM). ^{1}H NMR (400 MHz, $CDCl_3$) δ 7.75 – 7.71 (m, 2H), 7.42 – 7.38 (m, 2H), 2.87 (s, 6H). $^{13}C\{^{1}H\}$ NMR (101 MHz, $CDCl_3$) δ 165.46, 155.67, 132.70, 131.82, 130.37, 126.17, 115.13, 107.26, 24.86. MS (ESI-TOF): m/z $[M]^+$ calcd for $C_{15}H_{10}BrN_3$, 311.006; found, 312.135 (81.59%), 314.111 (100.00%), 315.175 (13.18%). Characterization is in agreement with that reported in the literature.[2]

Synthesis of 4-(4'-(12*H*-benzo[4,5]thieno[2,3-*a*]carbazol-12-yl)phenyl)-2,6-dimethylpyridine-3,5-dicarbonitrile (TCz-Me)

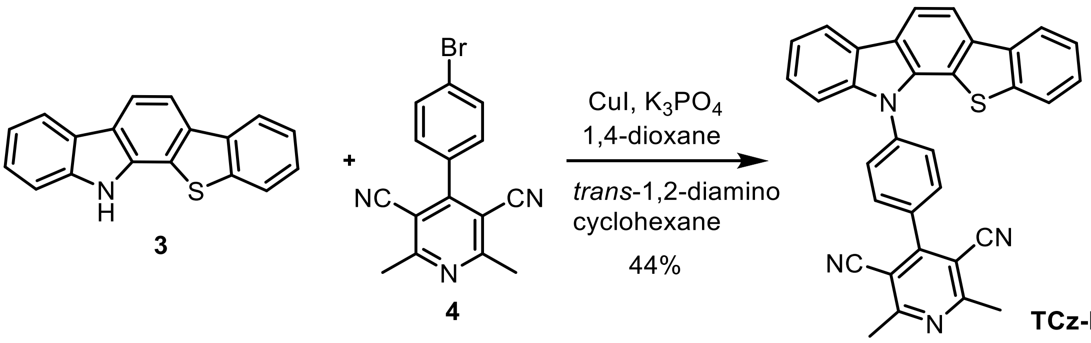

An oven-dried 2-necked round bottom flask equipped with a reflux condenser was degassed under vacuum and backfilled with argon, which was repeated 3-times. **3** (256 mg, 0.94 mmol), **4** (293 mg, 0.94 mmol), K_3PO_4 (399 mg, 1.88 mmol) and CuI (179 mg, 0.94 mmol) were added to the flask. 1,4-Dioxane (8 mL) and *trans*-1,2-diaminocyclohexane (0.23 mL, 1.88 mmol) were added and the resultant mixture was heated to 110 °C for overnight. The mixture was cooled to RT, diluted with THF (25 mL) and filtered. Solvents were evaporated on a rotary evaporator. The crude product was purified by column chromatography by using silica gel and PE/THF (2/1, v/v) solvent mixture (R_f = 0.33). Yield of **TCz-Me** = 205 mg (44%). ^{1}H NMR (700 MHz, $CDCl_3$) δ 8.25 (d, J = 8.3 Hz, 1H), 8.23 (dt, J = 7.8, 1.0 Hz, 2H), 8.14 (d, J = 8.2 Hz, 1H), 7.85 – 7.81 (m, 4H), 7.77 (dt, J = 7.9, 0.9 Hz, 1H), 7.50 – 7.43 (m, 3H), 7.39 (dddd, J = 9.9, 7.9, 6.9, 1.2 Hz, 2H), 2.95 (s, 6H). $^{13}C\{^{1}H\}$ NMR (176 MHz, $CDCl_3$) δ 165.46, 155.99, 141.48, 139.58, 139.37, 135.79, 135.60, 135.51, 133.71, 130.37, 130.25, 126.39, 126.23, 124.64, 124.05, 122.90, 121.98, 121.68, 121.60, 121.03, 120.47, 117.43, 115.19, 114.43, 110.21, 107.70, 24.93. MS (ESI-TOF): m/z $[M+H]^+$ calcd for $C_{33}H_{21}N_4S$, 505.149; found, 505.231 (100.00%), 506.371 (25.50%). HRMS (ASAP): m/z $[M+H]^+$ calculated for $C_{33}H_{21}N_4S$, 505.1487; found, 505.1483.

Synthesis of 3-oxo-3-[4′-(trifluoromethyl)phenyl]propanenitrile (5)

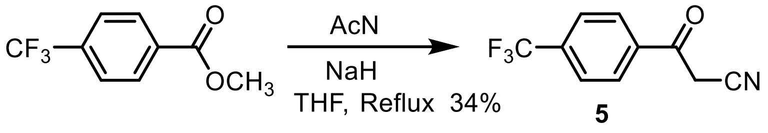

To a mixture of methyl 4-(trifluoromethyl)benzoate (0.79 mL, 4.90 mmol) and acetonitrile (0.31 mL, 5.88 mmol) was added anhydrous THF (15 mL) and NaH (0.24 g, 5.88 mmol). The resulting mixture was stirred at reflux under an inert atmosphere for 2 h. The mixture was cooled to 0 °C, and quenched with 2 M aq HCl until the pH was neutral. The crude product was extracted with EtOAc (25 mL x 2), washed with brine (25 mL) and dried over $MgSO_4$. The crude product was purified by column chromatography by using silica gel and hexane/EtOAc (10/1 to 10/4, v/v) solvent mixture. (R_f = 0.15, hex/EtOAc, 10/1,v/v). Yield = 0.35 g (34%). ^{1}H NMR (400 MHz, $CDCl_3$) δ 8.05 (ddt, *J* = 7.7, 1.6, 0.8 Hz, 2H), 7.83 – 7.78 (m, 2H), 4.14 (s, 2H). ^{13}C{^{1}H} NMR (101 MHz, $CDCl_3$) δ 186.43, 136.79, 135.94 (q, *J* = 33.0 Hz), 128.90, 126.31 (q, *J* = 3.7 Hz), 123.22 (q, *J* = 274 Hz), 113.28, 29.78. Characterization is in agreement with that reported in the literature.[3]

Synthesis of 4-(4'-bromophenyl)-2,6-bis[4″-(trifluoromethyl)phenyl]pyridine-3,5-dicarbonitrile (6)

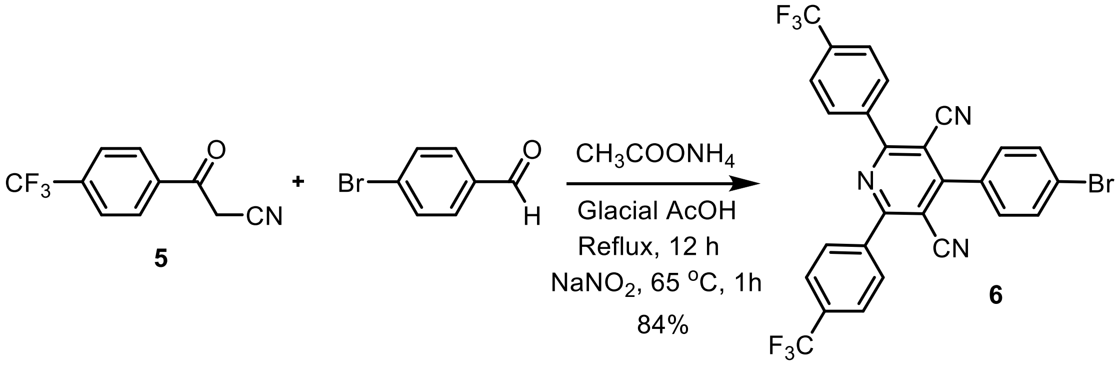

Compound **5** (310 mg, 1.45 mmol), 4-bromobenzaldehyde (107 mg, 0.58 mmol) and ammonium acetate (447 mg, 5.8 mmol) were refluxed in glacial acetic acid (20 mL) for 12 h under an Ar atmosphere. The temperature was then lowered to 65 °C and $NaNO_2$ (400 mg, 5.8 mmol) was added. The mixture was stirred for 2 h at this temperature. After cooling to RT, the mixture was diluted with ice-water (100 mL) and neutralized with ammonia. The precipitate was filtered off and washed with water to afford an off-white powder. The crude product was purified by column chromatography [silica gel, hexane/EtOAc (10/0.5 to 10/2, v/v)]. R_f = 0.19 (hexane/EtOAc, 10/1, v/v). Yield = 0.28 g (84%). ^{1}H NMR (400 MHz, $CDCl_3$) δ 8.20 – 8.14 (m, 4H), 7.88 – 7.83 (m, 4H), 7.83 – 7.78 (m, 2H), 7.55 – 7.50 (m, 2H). ^{13}C{^{1}H} NMR (101 MHz, $CDCl_3$) δ 162.35, 159.63, 139.23, 133.67, 133.34, 132.93, 131.81, 130.52, 130.12, 126.69, 126.08 (q, *J* = 3.8 Hz), 115.19, 107.06. ^{19}F{^{1}H} NMR (376 MHz, $CDCl_3$) δ -63.02 (s). MS (ESI-TOF): m/z $[M+H]^+$ calcd for $C_{27}H_{13}BrF_6N_3$, 572.020; found, 572.269 (71.18%), 574.245 (84.46%).

Synthesis of 4-(4'-(12*H*-benzo[4,5]thieno[2,3-*a*]carbazol-12-yl)phenyl-2,6-bis[4″-(trifluoromethyl)phenyl]pyridine-3,5-dicarbonitrile (TCz-CF3)

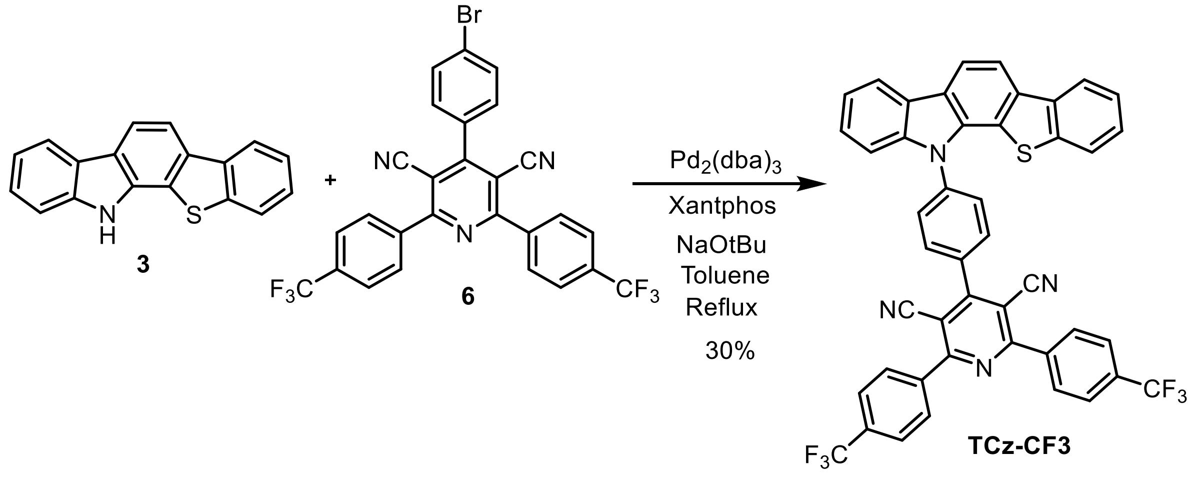

An oven-dried 2-necked 50 mL round bottom flask was degassed under vacuum and backfilled with argon, which was repeated 3-times. Compounds **3** (134 mg, 0.49 mmol) and **6** (0.28 g, 0.49 mmol) were transferred to the flask and kept under high vacuum for 10 min, then refilled with Ar gas. Dry toluene (15 mL) was added to the flask and Ar gas was bubbled through the solution for 20 min. $Pd_2(dba)_3$ (45 mg, 0.049 mmol) and Xantphos (85 mg, 0.147 mmol) were added to the flask and Ar gas was bubbled for 10 min. NaO*t*Bu (141 mg, 1.47 mmol) was added and the resulting solution was heated to reflux for overnight. The solvent was evaporated on a rotary evaporator and the resulting solid was adsorbed onto silica gel and purified by column chromatography by using petroleum ether (bp. 60-80 °C)/tol, 4/1 to 1/1, v/v solvent mixture. Yield of **TCz-CF3** = 112 mg (30%). R_f = 0.38 (PE/tol, 2/1, v/v). ^{1}H NMR (599 MHz, THF-d_8) δ 8.36 (d, *J* = 8.1 Hz, 4H), 8.31 – 8.27 (m, 2H), 8.25 (dd, *J* = 7.8, 1.1 Hz, 1H), 8.18 (dd, *J* = 8.3, 1.0 Hz, 1H), 8.09 – 8.07 (m, 2H), 7.99 – 7.93 (m, 6H), 7.69 (d, *J* = 7.8 Hz, 1H), 7.47 – 7.41 (m, 3H), 7.38 – 7.31 (m, 2H). ^{13}C NMR (151 MHz, THF-d_8) δ 162.67, 160.61, 142.68, 141.43, 140.84, 140.40, 136.91, 136.75, 136.42, 135.92, 133.59 (q, *J* = 32.7 Hz), 131.78, 131.49, 131.42, 127.14, 127.04, 126.73 (q, *J* = 3.8 Hz), 125.43, 125.28 (q, *J* = 183 Hz), 125.09, 123.55, 122.96, 122.77, 122.51, 121.75, 121.25, 118.27, 116.22, 115.24, 110.96. ^{19}F{^{1}H} NMR (376 MHz, THF-d_8) δ -63.63 (s). MS (ASAP-TOF): m/z $[M+H]^+$ calcd for $C_{45}H_{23}F_6N_4S$, 765.155; found, 765.127 (100.00%), 766.149 (88.95%). HRMS (ASAP): m/z $[M+H]^+$ calculated for $C_{45}H_{23}F_6N_4S$, 765.1548; found, 765.1550.

Synthesis of 4-{4'-(9*H*-carbazol-9-yl)phenyl}-2,6-bis[4″-(trifluoromethyl)phenyl]pyridine-3,5-dicarbonitrile (Cz-CF3)

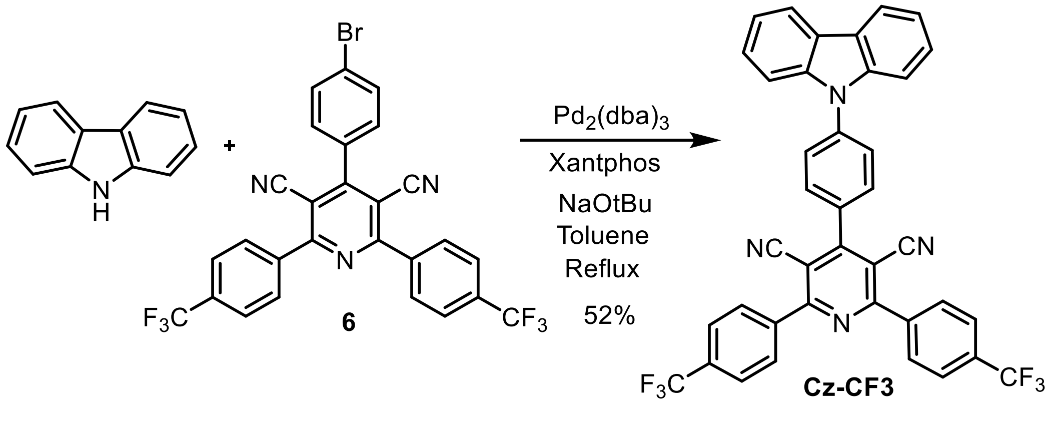

An oven-dried 2-necked 50 mL round bottom flask was degassed under vacuum and backfilled with argon, which was repeated 3-times. 9*H*-carbazole (59 mg, 0.35 mmol) and **6** (200 mg, 0.35 mmol) were transferred to the flask and kept under high vacuum for 10 min, then refilled with Ar gas. Dry toluene (10 mL) was added to the flask and Ar gas was bubbled through the solution for 20 min. $Pd_2(dba)_3$ (32 mg, 0.035 mmol) and Xantphos (61 mg, 0.105 mmol) were added to the flask and Ar gas was bubbled for 10 minutes. NaO*t*Bu (101 mg, 1.05 mmol) was added and the resulting solution was heated to reflux for overnight. The solvent was evaporated on a rotary evaporator and the resulting solid was adsorbed onto silica gel and purified by column chromatography by using PE (bp. 60-80 °C)/tol, 3/1 to 1/1, v/v, solvent mixture. Yield of **Cz-CF3** = 120 mg (52%). R_f = 0.16 (PE/tol, 1/1, v/v). ^{1}H NMR (700 MHz, CD_2Cl_2) δ 8.26 – 8.23 (m, 4H), 8.18 (dt, *J* = 7.8, 1.1 Hz, 2H), 7.97 – 7.92 (m, 4H), 7.92 – 7.89 (m, 4H), 7.60 (dt, *J* = 8.2, 0.8 Hz, 2H), 7.48 (ddd, *J* = 8.3, 7.1, 1.2 Hz, 2H), 7.35 (ddd, *J* = 7.9, 7.1, 0.9 Hz, 2H). ^{13}C NMR (176 MHz, CD_2Cl_2) δ 162.18, 159.50, 140.67, 140.29, 139.61 (q, *J* = 1.3 Hz), 132.88 (q, *J* = 32.8 Hz), 131.60, 130.87, 130.11, 127.19, 126.27, 125.80 (q, *J* = 3.8 Hz), 123.84 (q, *J* = 157 Hz), 123.79, 120.63, 120.37, 115.27, 109.82, 107.36. ^{19}F{^{1}H} NMR (376 MHz, CD_2Cl_2) δ -63.25 (s). MS (ASAP-TOF): m/z $[M+H]^+$ calcd for $C_{39}H_{21}F_6N_4$, 659.167; found, 659.172 (100.00%). HRMS (ASAP): m/z $[M+H]^+$ calculated for $C_{39}H_{21}F_6N_4$, 659.1670; found, 659.1665. Crystals of **Cz-CF3** for X-ray analysis were grown by slow evaporation from dichloromethane and methanol (2/1 v/v).

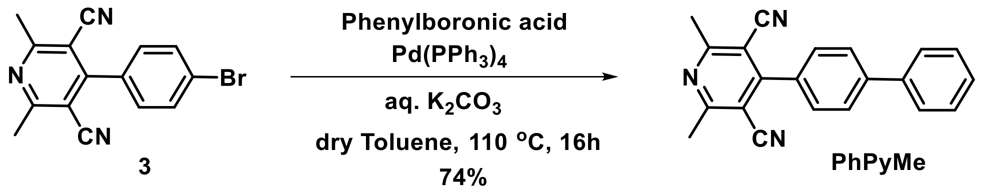

Synthesis of 4-[(1′,1′′-biphenyl)-4′-yl]-2,6-dimethylpyridine-3,5-dicarbonitrile (PhPyMe)

An oven-dried 2-necked 50 mL round bottom flask was degassed under vacuum and backfilled with argon three times. Compound **3** (150 mg, 0.48 mmol) and phenylboronic acid (71 mg, 0.57 mmol) were added and further kept under high vacuum for 10 min, then refilled with Ar gas. Dry toluene (10 mL) was then added to the flask and Ar gas was bubbled through the solution for 20 min. $Pd(PPh_3)_4$ (56 mg, 0.048 mmol) was then added into the flask and Ar was bubbled for another 10 minutes, followed by the additon of 0.5 ml aqeous solution containing K_2CO_3 (100 mg, 0.72 mmol). The reaction mixture was then refluxed at 110 °C for 16 h. The solvent was evaporated on a rotary evaporator and the resulting solid was adsorbed onto silica gel and purified by column chromatography by using hexane/EtOAc, 8/1 to 3/1, v/v, solvent mixture. Yield of **PhPyMe** =109 mg (74%). R_f = 0.17 (hexane/EtOAc, 8/1, v/v). ^{1}H NMR (400 MHz, $CDCl_3$) δ 7.82-7.78 (m, 2H), 7.68-7.60 (m, 4H), 7.51-7.46 (m, 2H), 7.44-7.38 (m, 1H). ^{13}C{^{1}H} NMR (101 MHz, $CDCl_3$) δ 165.24, 156.43, 144.01, 139.72, 131.61, 129.29, 128.99, 128.18, 127.82, 127.31, 115.34, 107.29, 24.74. HRMS (ESI): m/z $[M+H]^+$ calculated for $C_{33}H_{18}N_3F_6$, 570.1405; found, 570.1409. Crystals of **PhPyMe** for X-ray analysis were grown by slow evaporation from dichloromethane and hexane (1/1 v/v).

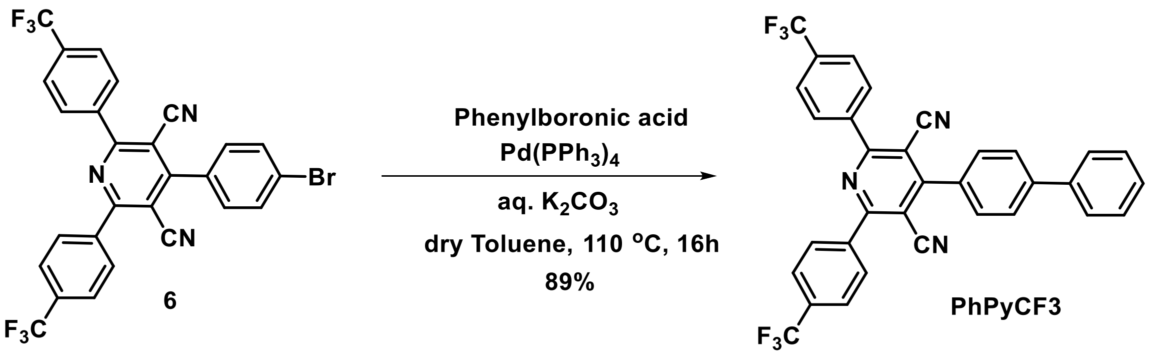

Synthesis of 4-[(1′,1″-biphenyl)-4-yl]-2,6-bis[4‴-(trifluoromethyl)phenyl]pyridine-3,5-dicarbonitrile (PhPyCF3)

An oven-dried 2-necked 50 mL round bottom flask was degassed under vacuum and backfilled with argon three times. Compound **6** (200 mg, 0.35 mmol) and phenylboronic acid (52 mg, 0.42 mmol) were added and further kept under high vacuum for 10 min, then refilled with Ar gas. Dry toluene (10 mL) was then added to the flask and Ar gas was bubbled through the solution for 20 min. $Pd(PPh_3)_4$ (40 mg, 0.035 mmol) was then added into the flask and Ar was bubbled for another 10 minutes, followed by the additon of 0.5 mL aqueous solution containing K_2CO_3 (73 mg, 0.52 mmol). The reaction mixture was then refluxed at 110 °C for 16 h. The solvent was evaporated on a rotary evaporator and the resulting solid was adsorbed onto silica gel and purified by column chromatography by using hexane/EtOAc, 10/1 to 5/1, v/v, solvent mixture. Yield of **PhPyCF3** = 178 mg (89%). R_f = 0.21 (hexane/EtOAc, 10/1, v/v). ^{1}H NMR (400 MHz, $CDCl_3$) δ 8.22-8.16 (m, 4H), 7.89-7.84 (m, 6H), 7.77-7.67 (4H, M, 7.54-7.48 (m, 2H), 7.46-7.40 (m, 1H). ^{13}C{^{1}H} NMR (101 MHz, $CDCl_3$) δ 162.24, 160.37, 144.46, 139.56, 139.33, 133.41, 133.09, 131.56, 130.03, 129.49, 129.04, 128.34, 127.99, 127.35, 125.91 (q, *J* = 3.7 Hz), 125.01, 122.30 [q (but only 2 peaks observed), *J* = 274 Hz)], 115.36, 107.11. ^{19}F{^{1}H} NMR (376 MHz, $CDCl_3$) δ -63.01 (s). HRMS (ESI): m/z $[M+H]^+$ calculated for $C_{21}H_{16}N_3$, 310.1344; found, 310.1361.

2. Thermogravimetric Analysis (TGA) Measurements

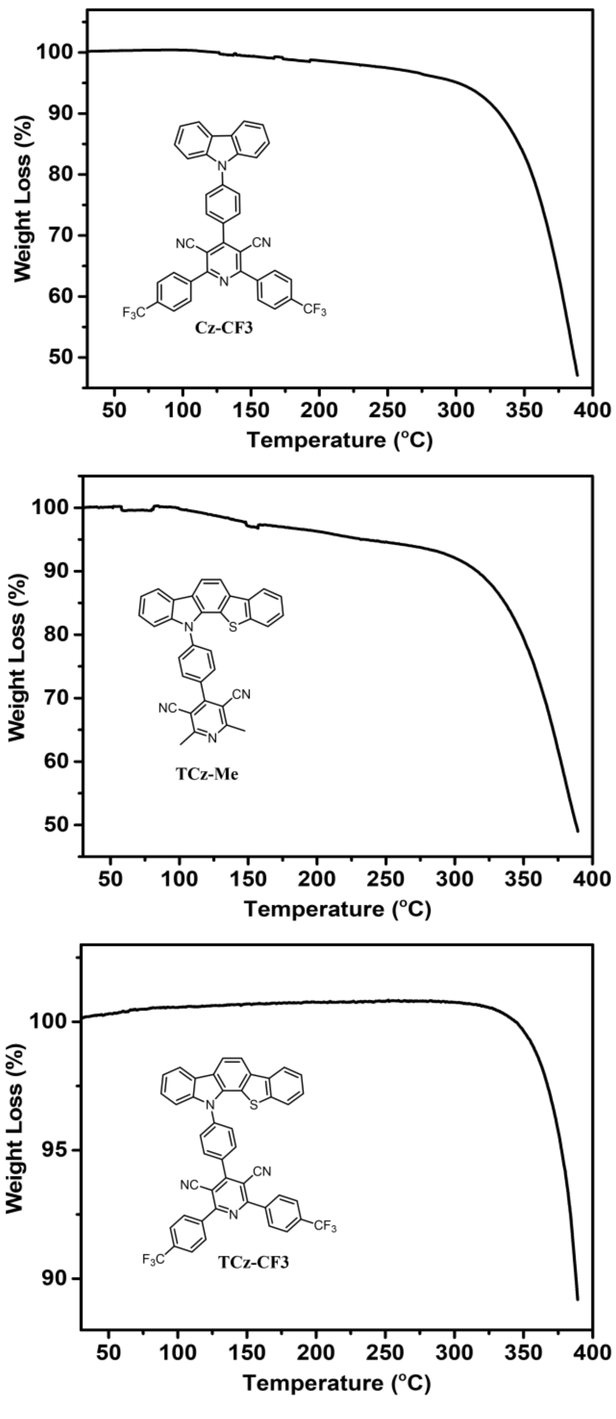

Figure S1. TGA curves for **Cz-CF3**, **TCz-Me** and **TCz-CF3**.

3. Cyclic Voltammetry (CV) Measurements

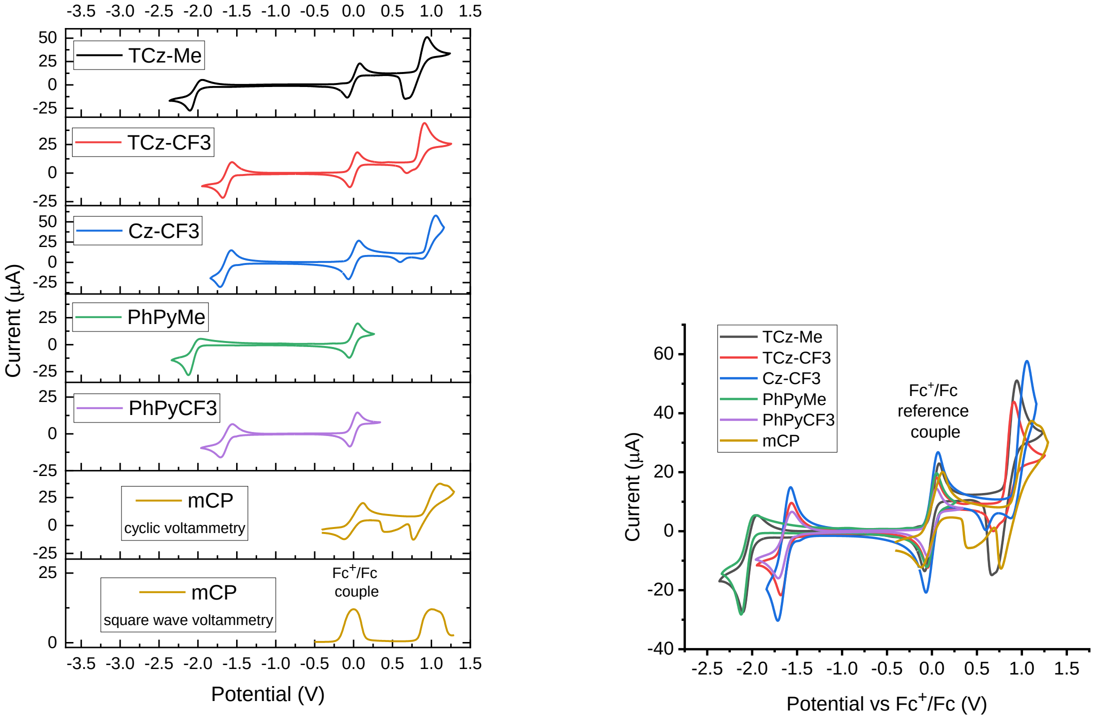

Figure S2. Cyclic voltammetry (CV) traces in DCM solutions with the internal ferrocenium/ferrocene (Fc^+/Fc) reference couple at 0.0 V. The square wave voltammetry (SWV) trace is included for mCP as mCP polymerizes rapidly after first oxidation and the oxidation potential for mCP is accurately measured against the internal Fc^+/Fc couple in the SWV trace.

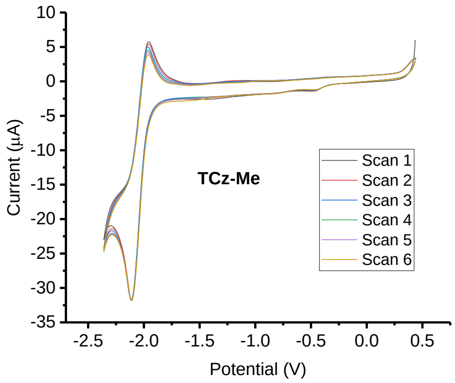

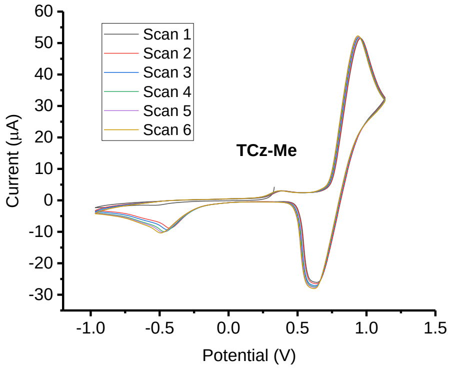

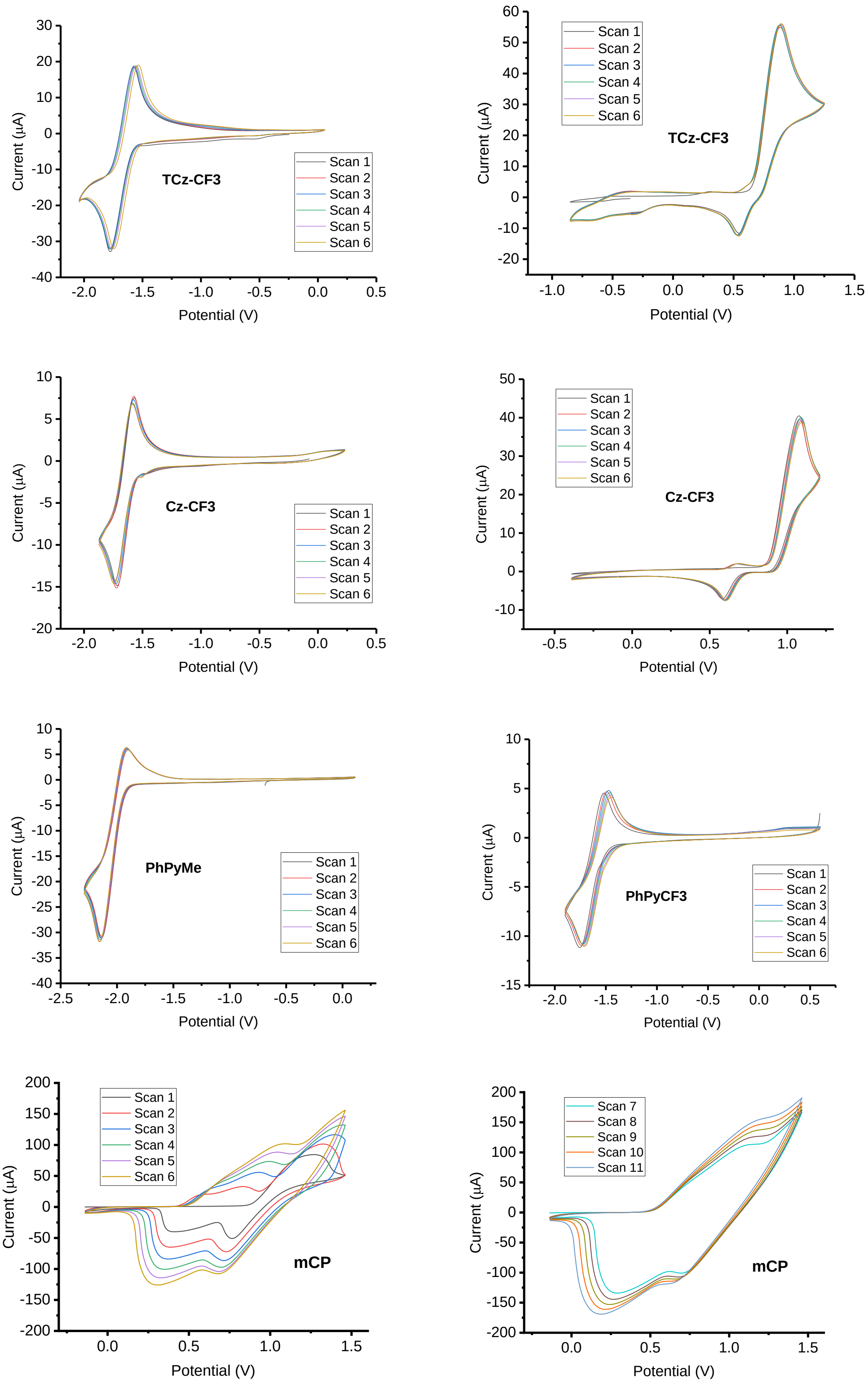

Figure S3. Repeated CV scan cycles. The CV scans for mCP change rapidly whereas repeated CV scans for other systems remain stable.

Table S1. Electrochemical redox potentials with Fc/Fc^+ internal reference couple at 0.00 V in 0.1 M *n*-Bu_4NPF_6 / DCM and HOMO-LUMO energies.

Compound	E_{ox} (V)[a]	E_{red} (V)[b]	E_{HOMO} (eV)[c/d]	E_{LUMO} (eV)[e/f]	E_g, CV (eV)[g]	E_g (opt) (eV)[h]
**TCz-Me**	0.85	-2.03	-5.65	-2.77/-2.65	2.88	3.0
**TCz-CF3**	0.86	-1.62	-5.66	-3.18/-2.96	2.48	2.7
**Cz-CF3**	0.98	-1.64	-5.78	-3.16/-2.98	2.62	2.8
**PhPyMe**	na	-2.06	na/-6.24	-2.74	na	3.5
**PhPyCF3**	na	-1.66	na/-6.44	-3.14	na	3.3
Cz-Me	0.95	-2.02	-5.75[i]	-2.78[i]/-2.78	2.97	3.12[j]
TCz	0.78	na	-5.58	na	na	na
Cz[k]	0.82	na	-5.62	na/-2.12	na	3.5
mCP	0.98	na	-5.78	na/-2.18	na	3.6

[a] oxidation potential.
[b] reduction potential.
[c] $E_{HOMO} = -(4.8 + E_{ox})$ eV. [4,5]
[d] $E_{HOMO} = E_{LUMO} - E_g$(opt) as estimated value in absence of the observed oxidation potential.
[e] $E_{LUMO} = -(4.8 + E_{red})$ eV.
[f] $E_{LUMO} = E_{HOMO} + E_g$(opt).
[g] E_g is the bandgap calculated from CV data [$E_g = E_{LUMO} - E_{HOMO}$].
[h] E_g (opt) is the bandgap obtained from the onset value of the absorption spectrum in toluene.
[i] Reference 6.
[j] Reference 7.
[k] Cz = 9*H*-carbazole

10. X-Ray Crystallography

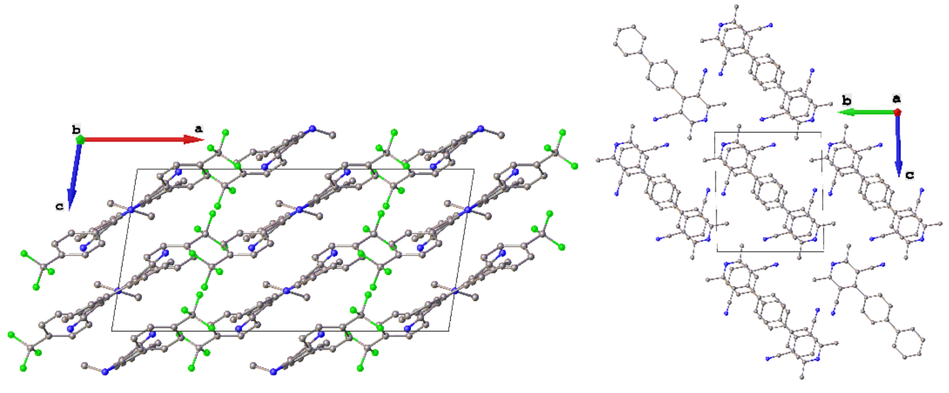

Figure S4. X-ray molecular structures of (left) **Cz-CF3** and (right**) PhPyMe** at 120 K.

Table S2. Crystal data and structure refinement.

	**Cz-CF3**	**PhPyMe**
CCDC	2320840	2320841
Empirical formula	$C_{39}H_{20}F_6N_4$	$C_{21}H_{15}N_3$
Formula weight	658.59	309.36
Temperature/K	120.00	120.00
Crystal system	monoclinic	triclinic
Space group	C2/c	P-1
a/Å	19.0062(9)	7.4643(3)
b/Å	17.5566(7)	10.4038(4)
c/Å	9.2495(4)	11.4723(5)
α/°	90	85.265(2)
β/°	99.104(2)	72.470(2)
γ/°	90	69.9260(10)
Volume/Å^3	3047.5(2)	797.71(6)
Z	4	2
ρ_{calc}g/cm^3	1.435	1.288
μ/mm^{-1}	0.111	0.078
F(000)	1344.0	324.0
Crystal size/mm^3	0.21 × 0.03 × 0.01	0.208 × 0.166 × 0.142
Radiation	Mo Kα (λ = 0.71073)	Mo Kα (λ = 0.71073)
2Θ range for data collection/°	4.34 to 51.998	4.17 to 74.938
Index ranges	$-23 \le h \le 23$, $-21 \le k \le 21$, $-11 \le l \le 11$	$-12 \le h \le 12$, $-17 \le k \le 17$, $-18 \le l \le 19$
Reflections collected	25343	37095
Independent reflections	2916 [R_{int} = 0.0663, R_{sigma} = 0.0389]	7639 [R_{int} = 0.0265, R_{sigma} = 0.0229]
Data/restraints/parameters	2916/0/224	7639/0/219
Goodness-of-fit on F^2	1.175	1.129
Final R indexes [I>=2σ (I)]	R_1 = 0.0577, wR_2 = 0.1055	R_1 = 0.0576, wR_2 = 0.1628
Final R indexes [all data]	R_1 = 0.0746, wR_2 = 0.1109	R_1 = 0.0645, wR_2 = 0.1675
Largest diff. peak/hole / e Å^{-3}	0.23/-0.29	0.59/-0.28

5. Ground-State Calculations

Ground-state Structures

Detailed hybrid density functional theory (DFT) and time dependent DFT (TD-DFT) computations were performed on the three D-A systems (**TCz-Me**, **TCz-CF3**, and **Cz-CF3**). The B3LYP/6-31G(d) model chemistry applied here generated excellent agreement between experimental (X-ray) and optimized geometries for **Cz-CF3** and **PhPyMe** (Figure S5 and Table S3). The optimized ground state (S_0) geometries for **TCz-Me**, **TCz-CF3** and **Cz-CF3** showed C-N-C-C dihedral angles of 50-61° between the (thieno)carbazolyl group and the *para*-phenylene moiety (Table S3). The C-C-C-C torsion angles are also around 52-64° between the *para*-phenylene linker and the pyridyl group. These torsion angles indicate that the *para*-phenylene bridges between the donor and acceptor groups are not orthogonal in the most stable conformers in the ground state. The orthogonal conformers are instead estimated to be higher in energy than the lowest energy conformers (by 1.2 kcal mol^{-1} for **TCz-Me**, 0.8 kcal mol^{-1} for **TCz-CF3** and 1.8 kcal mol^{-1} for **Cz-CF3**) and thus would be present in solutions at ambient temperature.

Electronic structure calculations on the ground state geometries of the D-A molecules show that the HOMOs are on the (thieno)carbazolyl donor group and the LUMOs on the pyridyl acceptor group, according to prior expectations (Figure S6). The HOMO energies are all similar, with the thienocarbazole higher than the carbazole moiety by 0.16-0.20 eV due to extended conjugation involving the benzothieno moiety. While the LUMO energies for **Cz-CF3** and **TCz-CF3** are nearly identical, the LUMO energy in **TCz-Me** is considerably (0.7 eV) higher. The LUMO in **TCz-Me** also contains some *para*-phenylene character, and thus there is a degree of HOMO-LUMO overlap at the *para*-phenylene. The HOMO-LUMO overlap at the *para*-phenylene for the orthogonal conformers in these DA systems is essentially zero. The trends in the HOMO and LUMO energies from the observed CV data (Table S1) and the corresponding calculated frontier MOs (Figure S6) are in broad agreement. The S_0 optimized geometries of the model acceptor fragments **PhPyMe** and **PhPyCF3** also feature non-orthogonal conformers (Table S3), but differ in their orbital compositions (Figure S7). **PhPyMe** has considerable HOMO-LUMO overlap (22%) at the *para*-phenylene ring while **PhPyCF3** has near zero HOMO-LUMO overlap. The molecule **PhPyCF3** may formally be regarded as a D-A system like **TCz-CF3** and **Cz-CF3**.

Table S3. Dihedral angles in degrees between ring planes of (thieno)carbazolyl/phenyl groups and *para*-phenylene bridge and between *para*-phenylene bridge and pyridyl groups.

	S_0 C-N(C)-C-C	S_0 C-C-C(Py)-C(Py)	S_1 C-N(C)-C-C	S_1 C-C-C(Py)-C(Py)	T_1 C-N(C)-C-C	T_1 C-C-C(Py)-C(Py)
**TCz-Me**	50.15	52.39	77.92	71.16	49.66	39.71
**TCz-CF3**	60.82	54.77	90.00	89.98	54.12	48.91
**Cz-CF3**	61.44	64.33	88.45	76.99	71.11	54.77
**Cz-CF3** (expt)[a]	45.09	51.60				
**PhPyMe**	37.45	51.71	37.52	51.01	37.50	46.41
**PhPyMe** (expt)[a]	42.36	46.98				
**PhPyCF3**	37.54	52.43	21.30	85.05	37.36	39.77
**Cz-CF3 dimer**	46.19	49.68	48.77	52.15	45.21	47.93
**Cz-CF3: mCP**	42.90	55.33	43.84	61.99	48.23	49.68
**PhPyCF3: mCP**	22.10	48.66	19.54	42.46	22.34	38.63
**PhPyMe: mCP**	25.20	44.75	21.50	29.94	8.19	30.93
**TCz-CF3: mCP**	49.66	60.28	52.88	69.45	55.72	59.14

[a] X-ray crystallographic data

Table S4. Nearest centroid ring-centroid ring intermolecular distances in Å.

	S_0	S_1	T_1
**Cz-CF3 dimer**	3.602	3.706	3.662
**Cz-CF3** (expt)[a]	3.783		
**Cz-CF3: mCP**	3.642	3.616	3.770
**PhPyCF3: mCP**	3.646	3.518	3.293
**PhPyMe: mCP**	3.802	3.518	3.573
**TCz-CF3: mCP**	3.601	3.611	3.609

[a] X-ray crystallographic data

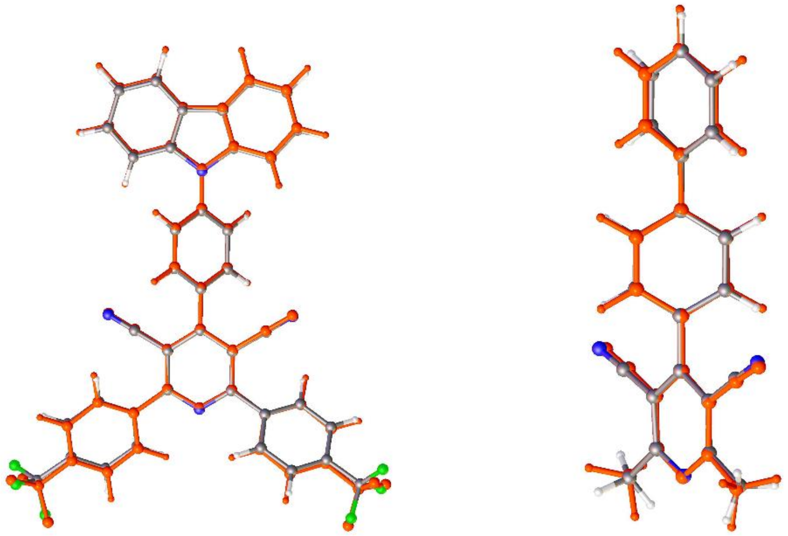

Figure S5. Geometry fittings between experimental (X-ray crystallography) and computed optimized geometries for **Cz-CF3** and **PhPyMe**. The optimized ground-state geometries are shown in orange. Misfit values in Å are 0.250 and 0.276, respectively, for all atoms.

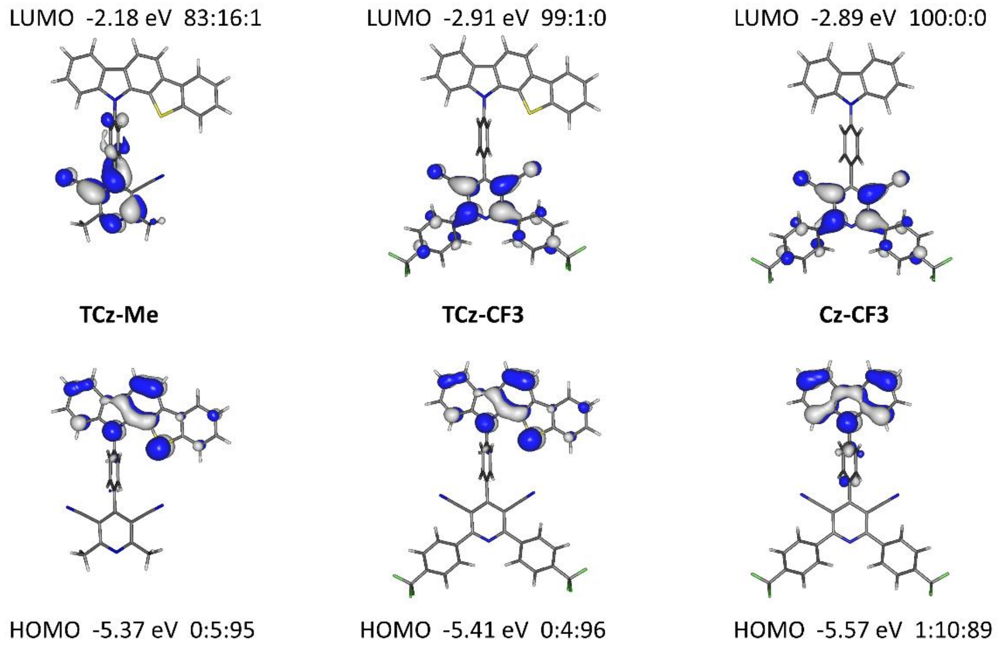

Figure S6. Frontier orbitals for **TCz-Me**, **TCz-CF3** and **Cz-CF3** with orbital energies and % orbital character ratios of pyridyl (acceptor) : bridging phenylene : (thieno)carbazole.

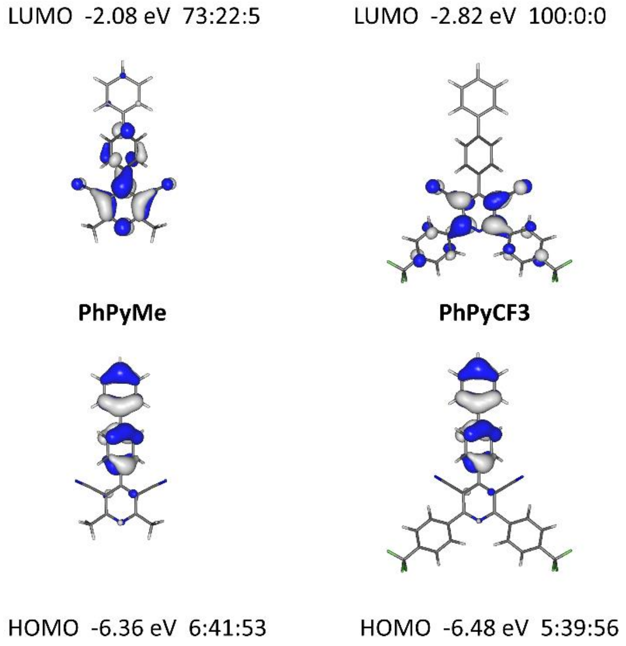

Figure S7. Frontier orbitals for **PyPhMe** and **PyPhCF3** with orbital energies and % orbital character ratios of pyridyl (acceptor) : bridging phenylene : phenyl.

6. Optical Properties

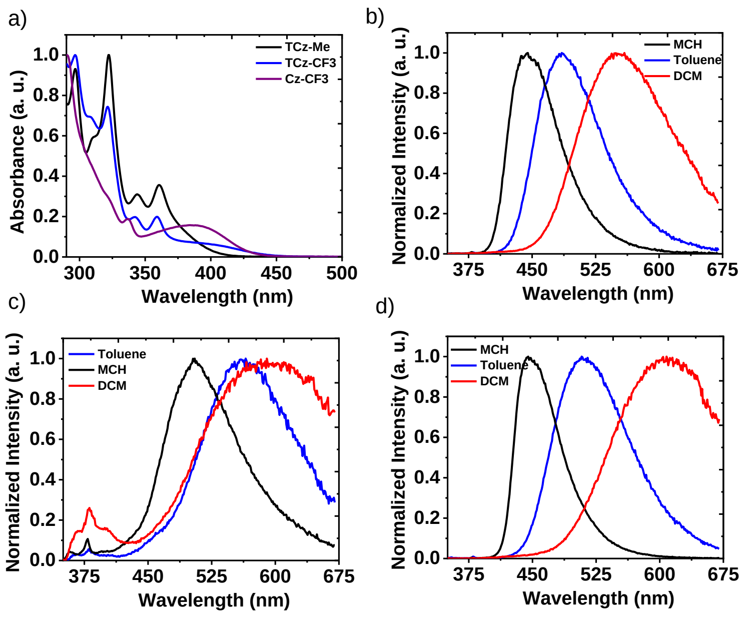

Figure S8. (a) Normalized absorbance of **TCz-Me**, **TCz-CF3** and **Cz-CF3** in 50 μM toluene solution. Solvatochromism studies of (b) **TCz-Me**, (c) **TCz-CF3** and (d) **Cz-CF3** excited at 350 nm. (MCH = methylcyclohexane; DCM = dichloromethane)

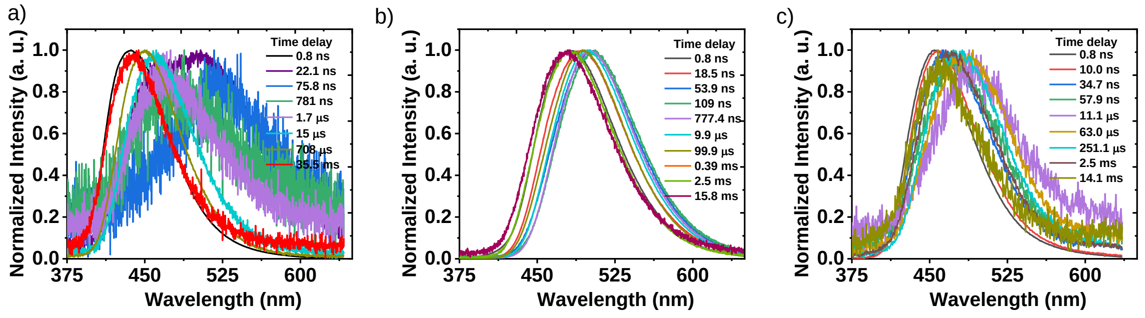

Figure S9. Time resolved emission spectra of (a) **TCz-Me** (b) **TCz-CF3** and (c) **Cz-CF3** films doped in 1 wt. % zeonex at room temperature. λ_{exc} = 355 nm.

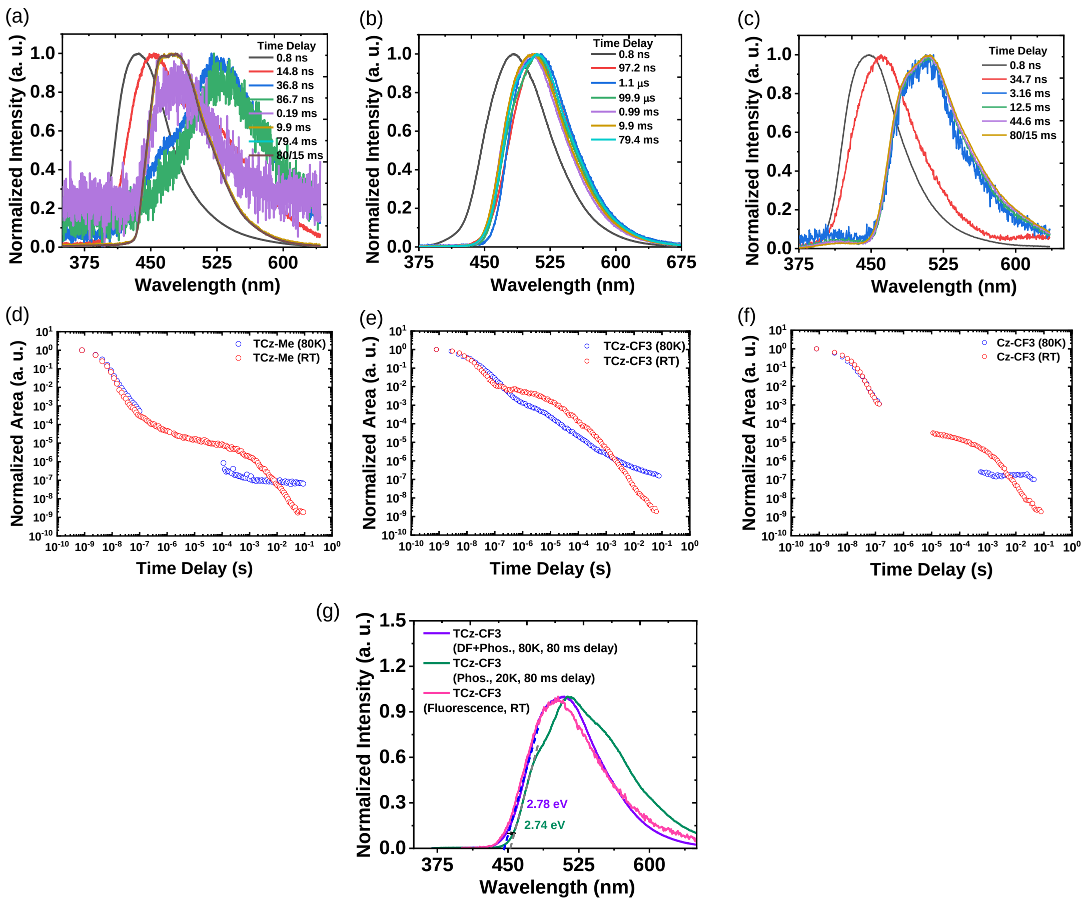

Figure S10. Time resolved emission spectra obtained at different delay times for (a) **TCz-Me**, (b) **TCz-CF3** and (c) **Cz-CF3** doped in zeonex (1 wt. %) at 80 K. Time-resolved emission decay of (d) **TCz-Me**, (e) **TCz-CF3** and (f) **Cz-CF3** films doped in zeonex (1 wt. %) at room temperature and 80 K. λ_{exc} = 355 nm. (g) Phosphorescence spectra of **TCz-CF3** at 20 K showing clear red-shift as compared to the time-resolved emission at 80K.

Note: We did not perform the phosphorescence of **Cz-CF3** and **TCz-Me** films at 20 K as the phosphorescence spectrum of each of these molecules are highly red-shifted as compared to their fluorescence spectrum and therefore, do not have any delayed fluorescence contribution at 80 K.

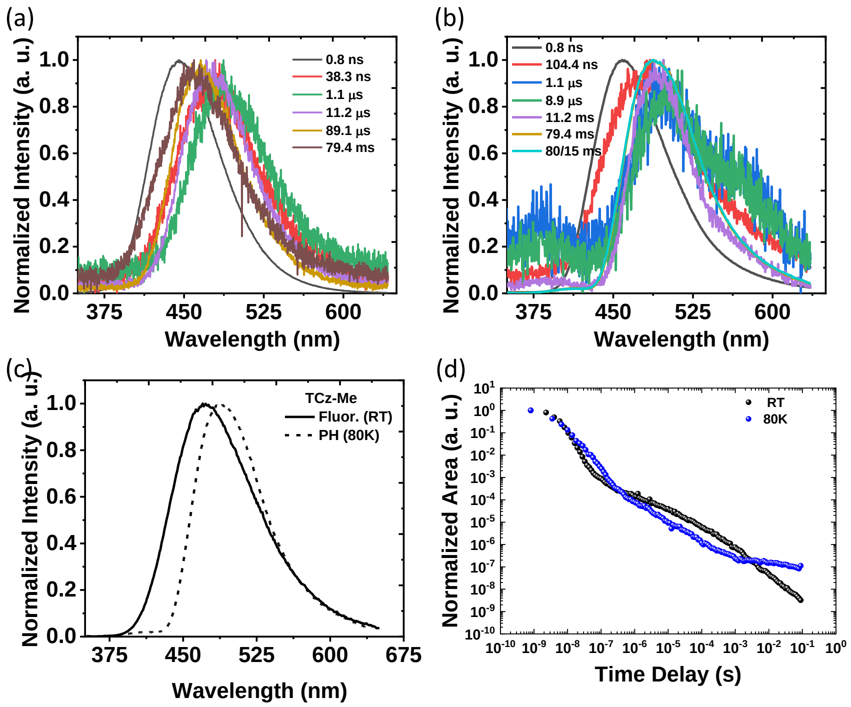

Figure S11. Time resolved spectra obtained at different delay times for **TCz-Me** doped in DPEPO at (a) RT and (b) 80 K. (c) Steady-state fluorescence and phosphorescence (delayed, delay time 80 ms and gate width 15 ms at 80 K) spectra of **TCz-Me** doped in DPEPO (10 wt. %). (d) Time-resolved emission decay of **TCz-Me** doped in DPEPO (10 wt%) at room temperature and 80 K. λ_{exc} = 355 nm.

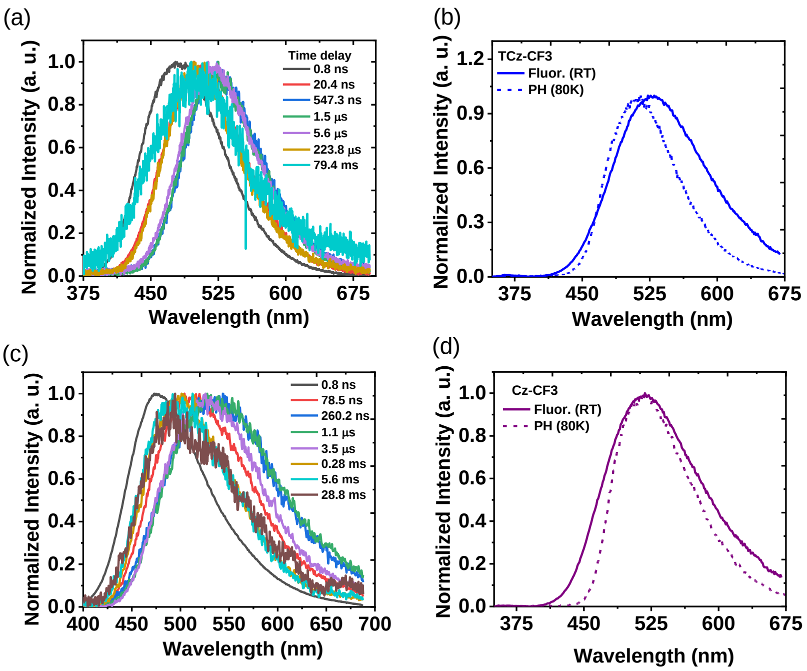

Figure S12. Time resolved emission spectra of (a) **TCz-CF3** (c) **Cz-CF3** films doped in 10 wt. % DPEPO, obtained at different delay times at room temperature. Steady-state and phosphorescence (PH, delayed, delay time 80 ms and gate width 15 ms at 80 K) spectra of (b) **TCz-CF3** and (d) **Cz-CF3** doped in 10 wt. % DPEPO. λ_{exc} = 355 nm.

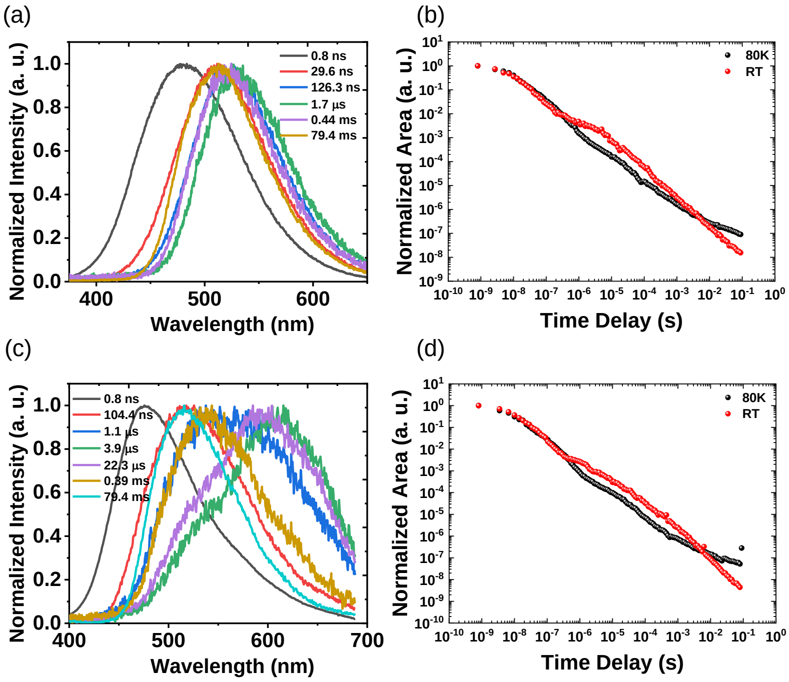

Figure S13. (a) Time resolved spectra at 80 K and (b) emission decay times for **TCz-CF3** doped in 10 wt. % DPEPO obtained at different delay times at both RT and 80 K. (c) Time resolved spectra at 80 K and (d) emission decay times for **Cz-CF3** films doped in 10 wt. % DPEPO. λ_{exc} = 355 nm.

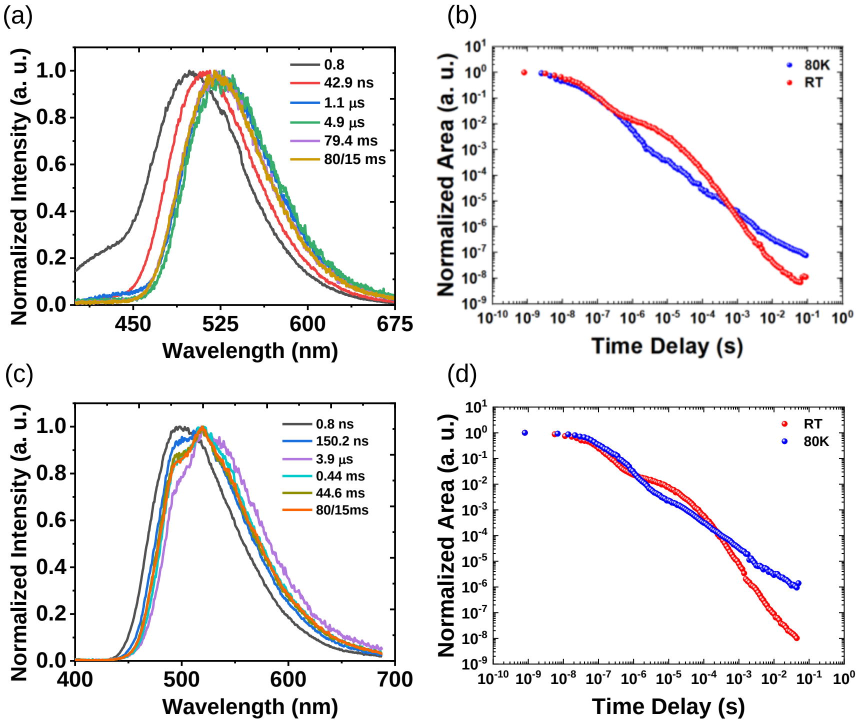

Figure S14. (a) Time resolved spectra at 80 K and (b) emission decay times for **TCz-CF3** doped in 10 wt. % mCP obtained at different delay times at both RT and 80 K. (c) Time resolved spectra at 80 K and (d) emission decay times for **Cz-CF3** films doped in 10 wt. % mCP. λ_{exc} = 355 nm.

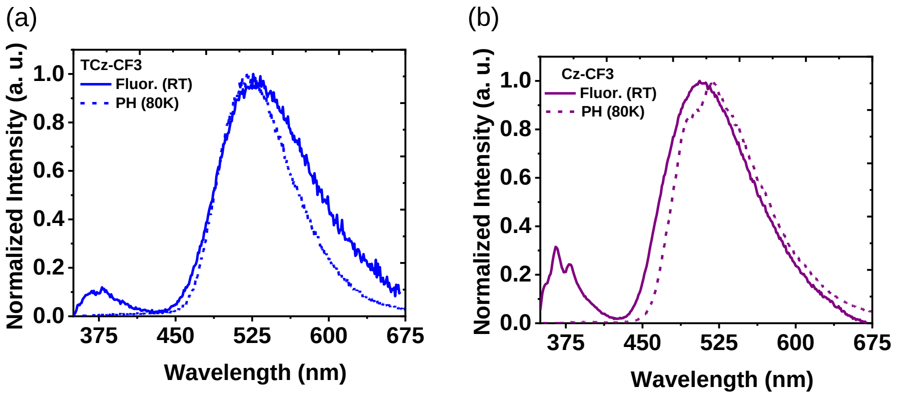

Figure S15. Steady-state and phosphorescence (delayed, delay time 80 ms and gate width 15 ms at 80 K) spectra of (a) **TCz-CF3** and (b) **Cz-CF3** doped in 10 wt. % mCP. λ_{exc} = 355 nm.

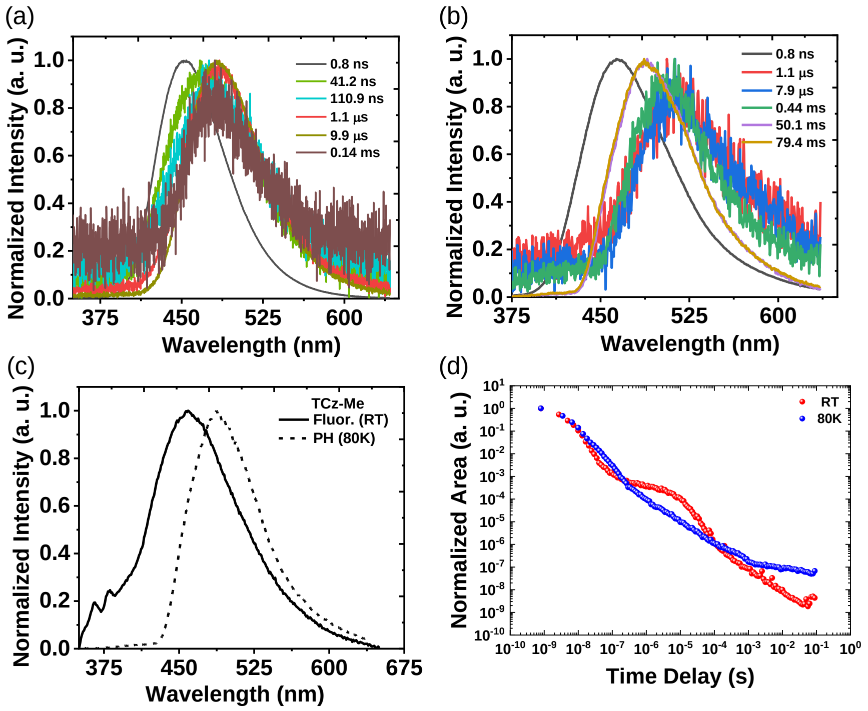

Figure S16. Time resolved spectra obtained at different delay times for **TCz-Me** doped in mCP at (a) RT and (b) 80 K. (c) Steady-state fluorescence and phosphorescence (delayed, delay time 80 ms and gate width 15 ms at 80 K) spectra of **TCz-Me** doped in mCP (10 wt. %). (d) Time-resolved emission decay of **TCz-Me** doped in mCP (10 wt%) at room temperature and 80 K.

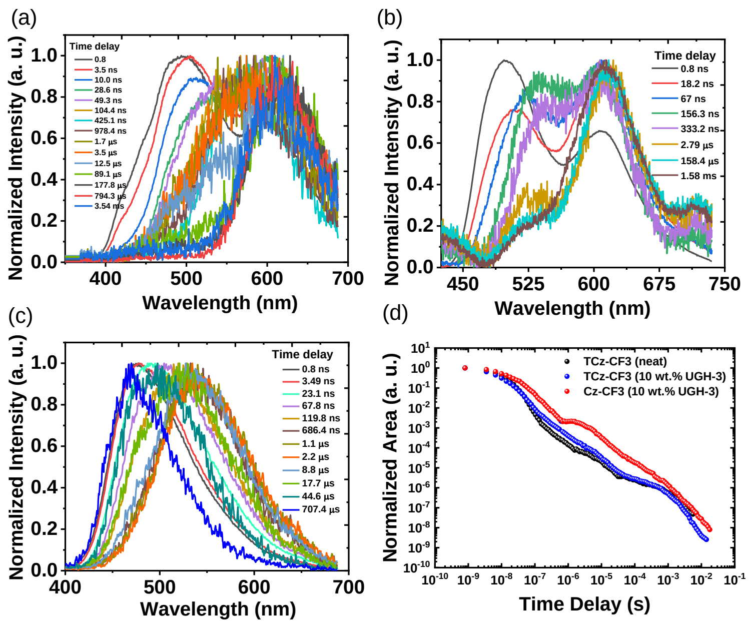

Figure S17. Time-resolved spectra obtained at different delay times for **TCz-CF3** doped in (a) 10 wt.% UGH-3 and (b) neat films. (c) Time-resolved spectra obtained at different delay times for **Cz-CF3** doped in 10 wt.% UGH-3. (d) Time-resolved emission decay of the same films. All measurements were done at RT. λ_{exc} = 355 nm.

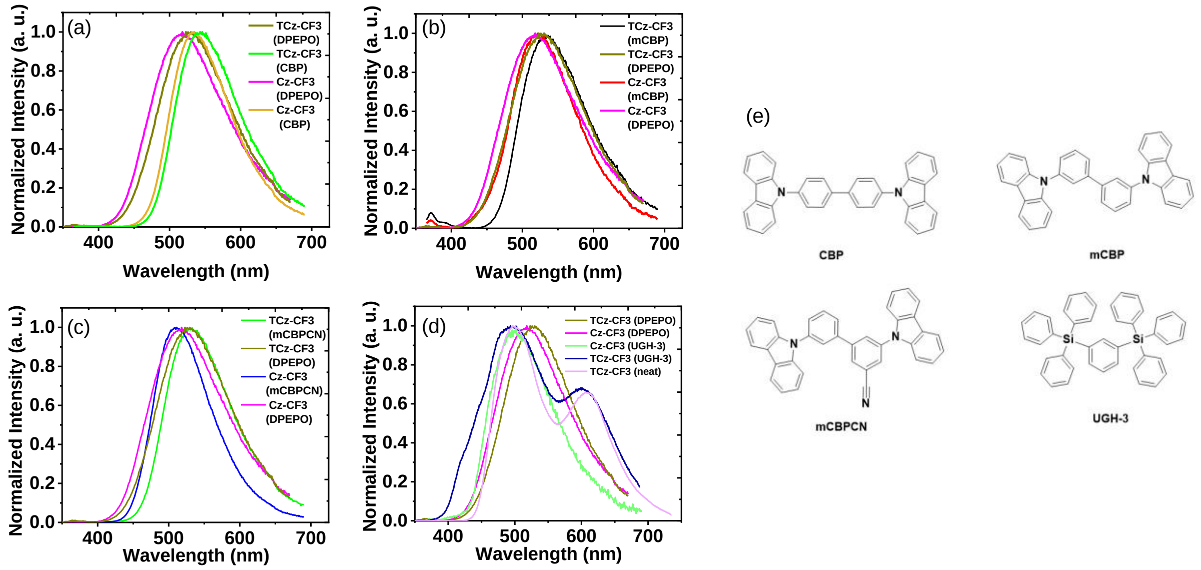

Figure S18. Comparison of normalized steady-state emission spectra for **TCz-CF3** and **Cz-CF3** films doped (10 wt.%) in DPEPO and (a) CBP, (b) mCBP, (c) mCBPCN and (d) UGH-3. (e) Molecular structures of the different host molecules studied. λ_{exc} = 355 nm.

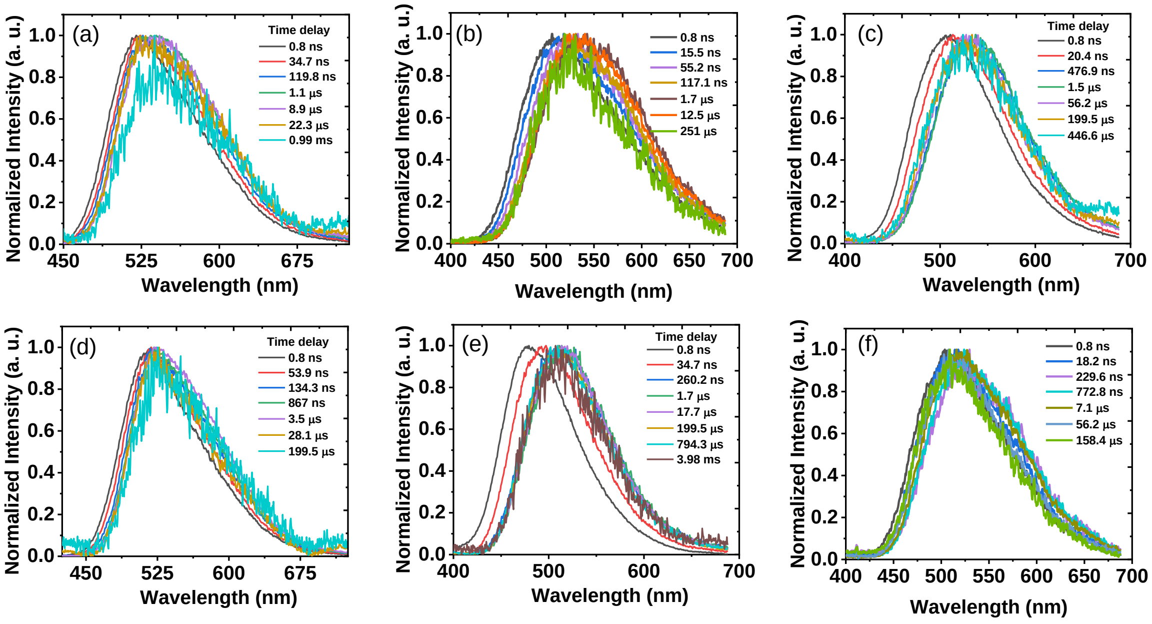

Figure S19. Time-resolved spectra obtained at different delay times for **TCz-CF3** doped in 10 wt.% (a) CBP, (b) mCBP and (c) mCBPCN. Time-resolved spectra obtained at different delay times for **Cz-CF3** doped in 10 wt.% (d) CBP, (e) mCBP and (f) mCBPCN. λ_{exc} = 355 nm.

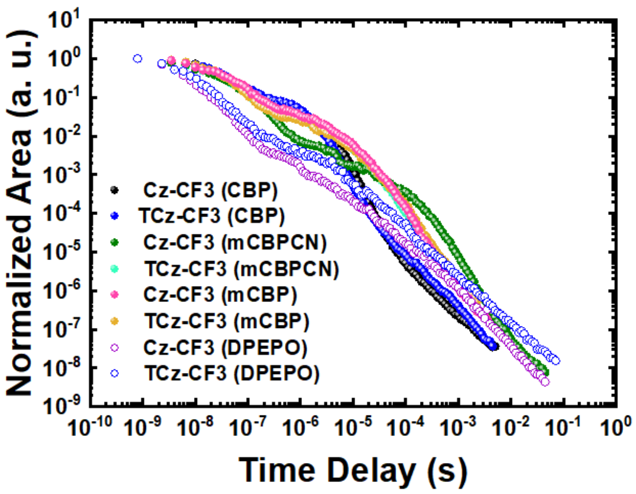

Figure S20. Time-resolved emission decay of the 10 wt. % doped films in various hosts at RT. λ_{exc} = 355 nm.

Note: CBP doped films show relatively fast decay in the delayed fluorescence region presumably due to a lower triplet energy and T_1 (Host) ← T_1 (Guest) Dexter energy transfer losses.

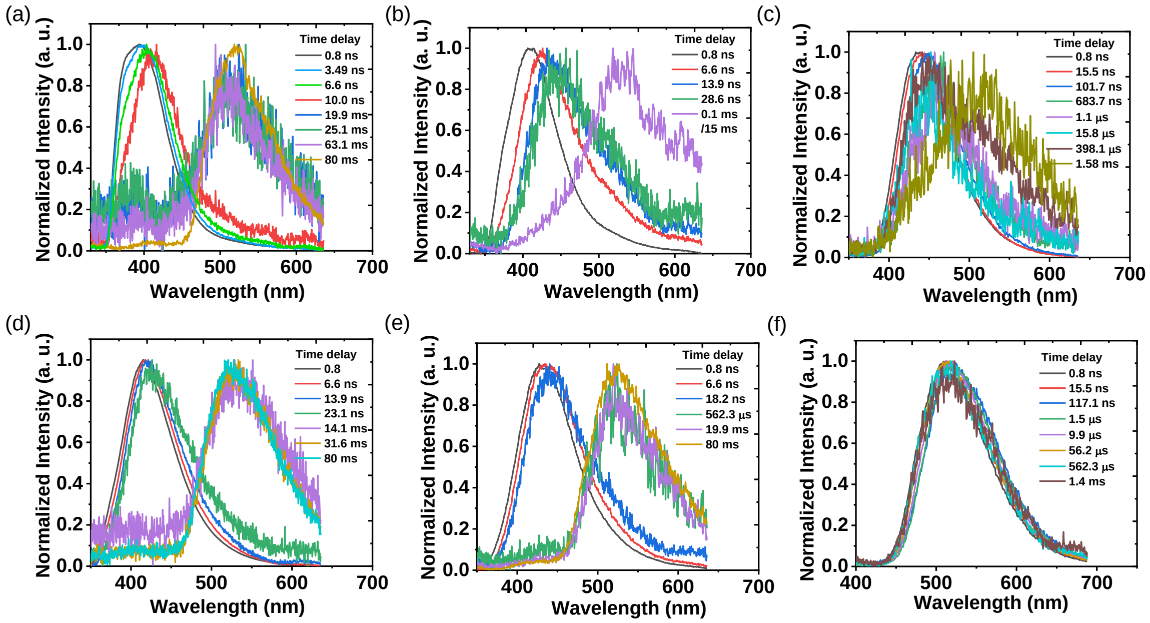

Figure S21. Time-resolved spectra obtained at different delay times for **PhPyMe** doped in (a) 1 wt.% zeonex, (b) 10 wt.% DPEPO and (c) 10 wt. % mCP. Time-resolved spectra obtained at different delay times for **PhPyCF3** doped in (d) 1 wt.% zeonex, (e) 10 wt.% DPEPO and (f) 10 wt. % mCP. λ_{exc} = 355 nm.

Note: Exciplex is formed in both **PhPyMe** and **PhPyCF3** doped films in mCP. In **PhPyCF3** the locally excited triplet state is closer in energy to the formed exciplex ^{1}CT, and considering the T_1 energy in zeonex, the ΔE_{ST} = 0.06 eV for **PhPyCF3** is very similar to the range of **CzCF3** (0.08 eV) and **TCz-CF3** (0.04 eV) (see Table S5). On the other hand, **PhPyMe** has a much larger gap (ΔE_{ST} = 0.49 eV). This results in a much more efficient TADF for the **PhPyCF3-mCP** doped films.

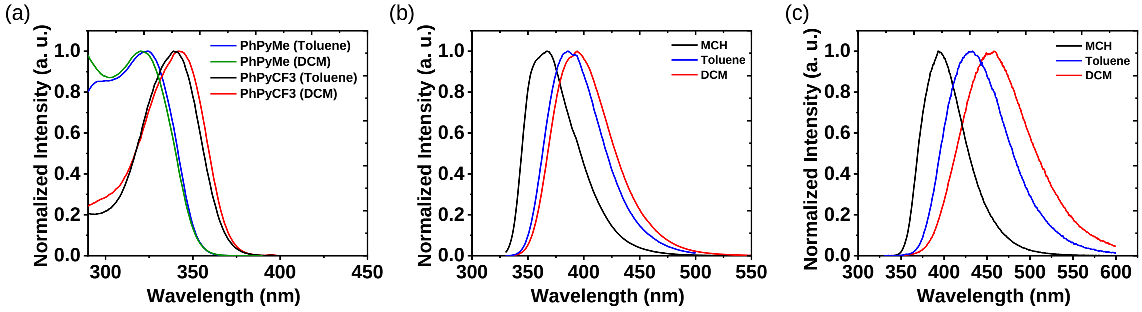

Figure S22. (a) Normalized excitation spectra (monitored at 425 nm) **PhPyMe** and **PhPyCF3** in 50 μM Toluene and DCM solutions. Normalized emission spectra (b) **PhPyMe** and (c) **PhPyCF3** in different solvent [(c) = 50 μM, λ_{exc} = 355 nm].

Note: Although solvatochromism is seen in both **PhPyMe** and **PhPyCF3**, suggesting their unexpected intramolecular charge-transfer characteristics, they do not show any TADF in dilute zeonex or DPEPO films, therefore, exciplex emission is the origin of their TADF when dispersed in mCP.

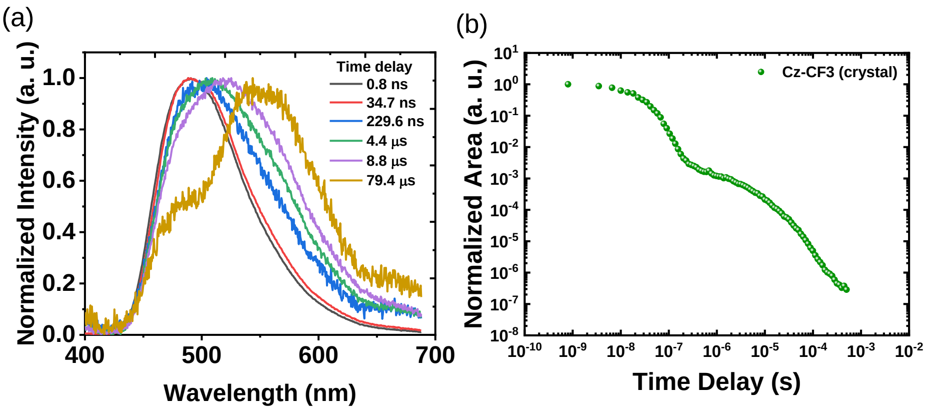

Figure S23. (a) Room temperature time-resolved spectra obtained at different delay times and (b) for **Cz-CF3** in crystalline state. λ_{exc} = 355 nm.

Note: No dispersion (fixed onset in the entire timescale) in the time-resolved spectrum suggests a strong π-π and charge-transfer interaction between two neighboring units. The new redshifted band at longer time delays suggests an aggregated species different from the initial charge-transfer species. It is worth noting that a similar CT state and aggregated emission band are also seen for neat films of **TCz-CF3** (Fig S17(b)).

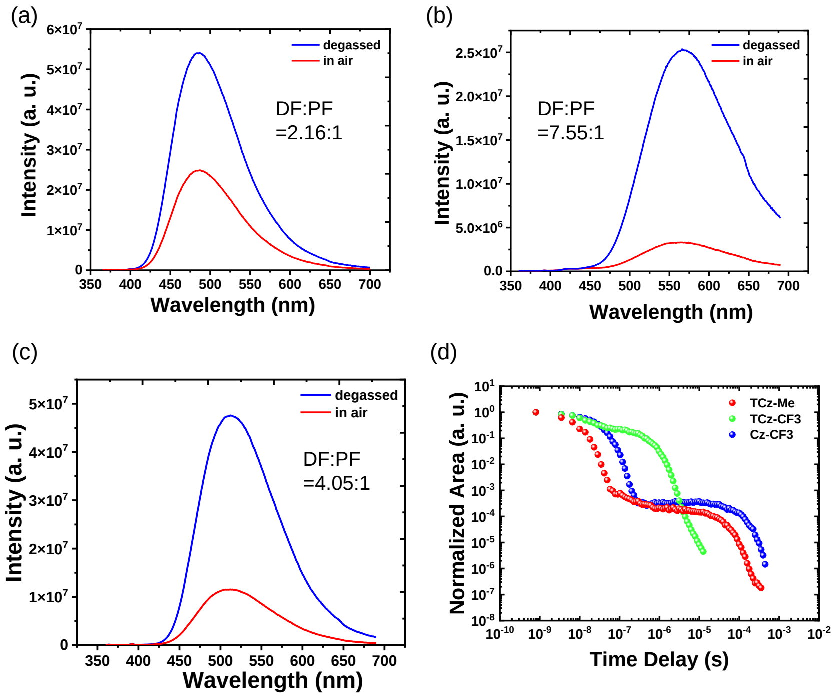

Figure S24. Steady-state emission spectra of (a) **TCz-Me**, (b) **TCz-CF3** and (c) **Cz-CF3** in dilute toluene solution (50 μM) under degassed conditions and in air. λ_{exc} = 350 nm. (d) Time-resolved emission decays of these solutions are also shown. λ_{exc} = 355 nm.

Table S5. Fitted exponential lifetimes and rate constants of emission decays.

Emitters	τ_{PF} [a] (ns)	τ_{DF} [a] (μs)	k_F [b] ($\times10^6$ s^{-1})	k_{ISC} [b] ($\times$ 10^6 s^{-1})	k_{rISC} [b] ($\times$ 10^5 s^{-1})	Φ_{PL} [c] (%)	S_1 (eV)	T_1 (eV)	ΔE_{ST} (eV)
	**10 wt. % in mCP**								
**TCz-Me**	3.36	8.3	60.0 ± 7.0	59.0 ± 4.2	1.6 ± 0.05	n.d.	3.11	2.86	0.25
**TCz-CF3**	37.1	9.03	4.0 ± 0.7	11.0 ± 1.1	4.0 ± 0.8	54±10	2.72	2.68	0.04
**Cz-CF3**	55.7	16.5	4.0 ± 0.8	9.0 ± 2.1	3.0 ± 0.7	64±10	2.8	2.72	0.08
	**10 wt. % in DPEPO**								
**TCz-Me**	3.9	13.7	*-	*-	*-	n.d.	3.05	2.83	0.22
**TCz-CF3**	15.5	8.3	*-	*-	*-	39±10	2.84	2.78	0.06
**Cz-CF3**	3.9	6.8	*-	*-	*-	53±10	2.85	2.74	0.11
	**1 wt. % in zeonex**								
**TCz-Me**	*-	*-	*-	*-	*-	n.d.	3.1	2.87	0.23
**TCz-CF3**	11.7	13.1	10.0 ± 0.01	60.0 ± 0.018	8.0 ± 0.01	n.d.	2.8	2.74	0.06
**Cz-CF3**	*-	*-	*-	*-	*-	n.d.	2.9	2.75	0.15
	**50 μM in degassed Toluene**								
**TCz-Me**	7.06	28	52.9 ± 5.7	63.8 ± 3.3	0.8 ± 0.06	n.d.	2.92	n.d.	n.d.
**TCz-CF3**	92	2.4	1.4 ± 0.03	10.5 ± 0.5	47.0 ± 1.6	n.d.	2.67	n.d.	n.d.
**Cz-CF3**	26.7	93.9	14 ± 0.6	22.5 ± 0.6	0.24 ± 0.01	n.d.	2.84	n.d.	n.d.

[a] amplitude-weighted average of lifetimes from bi-exponential fitting of PF or DF time regime; [b] rates from simultaneous kinetic fitting of PF and DF. Model assumptions in kinetic fitting make it only appropriate for TADF materials with strong DF and approximately single-exponential decay, therefore only applied to toluene solutions, mCP films for all three derivatives, only 1 wt.% zeonex films of **TCz-CF3**; [c] measured under nitrogen atmosphere; *strongly non-exponential. n.d. is not determined.

7. Excited-State Calculations

Table S6. Energies in eV and nature of the excited states from TD-DFT computations on optimized S_1 geometries of **TCz-Me**, **TCz-CF3** and **Cz-CF3** in argon. %CT values are listed in parentheses. ΔE_{ST} = S_1 energy – T_1 energy and ΔE_{TT} = T_2 energy – T_1 energy.

	T_1	T_2	S_1	T_3	ΔE_{ST}	ΔE_{TT}
**TCz-Me**	2.73 (17) ^{3}A	2.91 (0) ^{3}D	3.21 (99) ^{1}CT	3.15 (0) ^{3}D	0.48	0.19
**TCz-CF3**	2.34 (0) ^{3}A	2.55 (99) ^{3}CT	2.55 (99) ^{1}CT	2.98 (9) ^{3}D	0.21	0.21
**Cz-CF3**	2.27 (2) ^{3}A	2.91 (97) ^{3}CT	2.91 (99) ^{1}CT	3.06 (13) ^{3}A	0.64	0.64

Table S7. Energies in eV and nature of the excited states from TD-DFT computations on optimized S_1 geometries in toluene. %CT values are listed in parentheses. ΔE_{ST} = S_1 energy – T_1 energy and ΔE_{TT} = T_2 energy – T_1 energy. CT* = intermolecular CT.

	T_1	T_2	S_1	T_3	ΔE_{ST}	ΔE_{TT}
**TCz-Me**	2.74 (17) ^{3}A	2.91 (0) ^{3}D	3.03 (99) ^{1}CT	3.02 (99) ^{3}CT	0.29	0.17
**TCz-CF3**	2.35 (0) ^{3}A	2.43 (99) ^{3}CT	2.42 (99) ^{1}CT	2.98 (9) ^{3}D	0.07	0.08
**Cz-CF3**	2.27 (2) ^{3}A	2.61 (97) ^{3}CT	2.62 (99) ^{1}CT	3.06 (13) ^{3}A	0.35	0.34

**PhPyMe**	2.62 (9) ^{3}A	3.10 (30) ^{3}D	3.27 (9) ^{1}A	3.22 (9) ^{3}A	0.63	0.46
**PhPyCF3**	2.26 (0) ^{3}A	2.65 (12) ^{3}D	3.12 (98) ^{1}CT	3.07 (3) ^{3}A	0.86	0.39
**Cz-CF3 dimer**	2.23 (35) ^{3}A	2.39 (95) ^{3}CT*	2.40 (98) ^{1}CT*	2.71 (14) ^{3}A	0.17	0.16
**Cz-CF3: mCP**	2.25 (29) ^{3}A	2.46 (95) ^{3}CT*	2.47 (98) ^{1}CT*	3.03 (31) ^{3}A	0.22	0.21
**PhPyCF3: mCP**	2.20 (49) ^{3}A	2.43 (98) ^{3}CT*	2.44 (98) ^{1}CT*	2.79 (63) ^{3}CT	0.24	0.23
**PhPyMe: mCP**	2.34 (38) ^{3}D	2.94 (45) ^{3}CT*	2.91 (56) ^{1}CT*	3.11 (9) ^{3}D	0.57	0.60
**TCz-CF3: mCP**	2.24 (60) ^{3}A	2.46 (99) ^{3}CT*	2.47 (99) ^{1}CT*	2.82 (37) ^{3}D	0.23	0.22

Table S8. Energies in eV and nature of the excited states from TD-DFT computations on optimized S_1 geometries in DCM. %CT values are listed in parentheses. ΔE_{ST} = S_1 energy – T_1 energy and ΔE_{TT} = T_2 energy – T_1 energy. CT* = intermolecular CT.

	T_1	T_2	S_1	T_3	ΔE_{ST}	ΔE_{TT}
**TCz-Me**	2.74 (17) ^{3}A	2.85 (99) ^{3}CT	2.77 (99) ^{1}CT	2.92 (0) ^{3}D	0.03	0.11
**TCz-CF3**	2.29 (99) ^{3}CT	2.35 (0) ^{3}A	2.13 (99) ^{1}CT	2.99 (9) ^{3}D	-0.16	0.06
**Cz-CF3**	2.25 (97) ^{3}CT	2.28 (2) ^{3}A	2.25 (99) ^{1}CT	3.06 (13) ^{3}A	0.00	0.03
**PhPyMe**	2.65 (9) ^{3}A	3.10 (30) ^{3}D	3.23 (9) ^{1}A	3.13 (9) ^{3}A	0.58	0.45
**PhPyCF3**	2.27 (0) ^{3}A	2.66 (12) ^{3}D	2.83 (98) ^{1}CT	2.85 (98) ^{3}CT	0.56	0.39
**Cz-CF3 dimer**	2.20 (35) ^{3}A	2.34 (95) ^{3}CT*	2.28 (98) ^{1}CT*	2.62 (14) ^{3}A	0.08	0.14
**Cz-CF3: mCP**	2.26 (29) ^{3}A	2.33 (95) ^{3}CT*	2.32 (98) ^{1}CT*	3.03 (31) ^{3}A	0.06	0.07
**PhPyCF3: mCP**	2.20 (49) ^{3}A	2.31 (98) ^{3}CT*	2.28 (98) ^{1}CT*	2.79 (63) ^{3}CT	0.08	0.11
**PhPyMe: mCP**	2.34 (38) ^{3}D	2.93 (45) ^{3}CT*	2.71 (56) ^{1}CT*	3.12 (9) ^{3}D	0.37	0.59
**TCz-CF3: mCP**	2.25 (60) ^{3}A	2.34 (99) ^{3}CT*	2.33 (99) ^{1}CT*	2.82 (37) ^{3}D	0.08	0.09

Table S9. Calculated spin orbit coupling matrix elements (SOCME), $\langle S_1|\hat{H}_{so}|T_1\rangle$ and $\langle S_1|\hat{H}_{so}|T_n\rangle$ in cm^{-1} for optimized S_1 geometries of **TCz-Me**, **TCz-CF3** and **Cz-CF3** in toluene.

	$\langle S_1\|\hat{H}_{so}\|T_1\rangle$ cm^{-1}	Nature of T_1	%CT of T_1	$\langle S_1\|\hat{H}_{so}\|T_n\rangle$ cm^{-1}	Nature of T_n	%CT of T_n
**TCz-Me**	0.05	^{3}A	17	0.02	^{3}CT	99
**TCz-CF3**	0.11	^{3}A	0	0.04	^{3}CT	99
**Cz-CF3**	0.07	^{3}A	2	0.02	^{3}CT	97

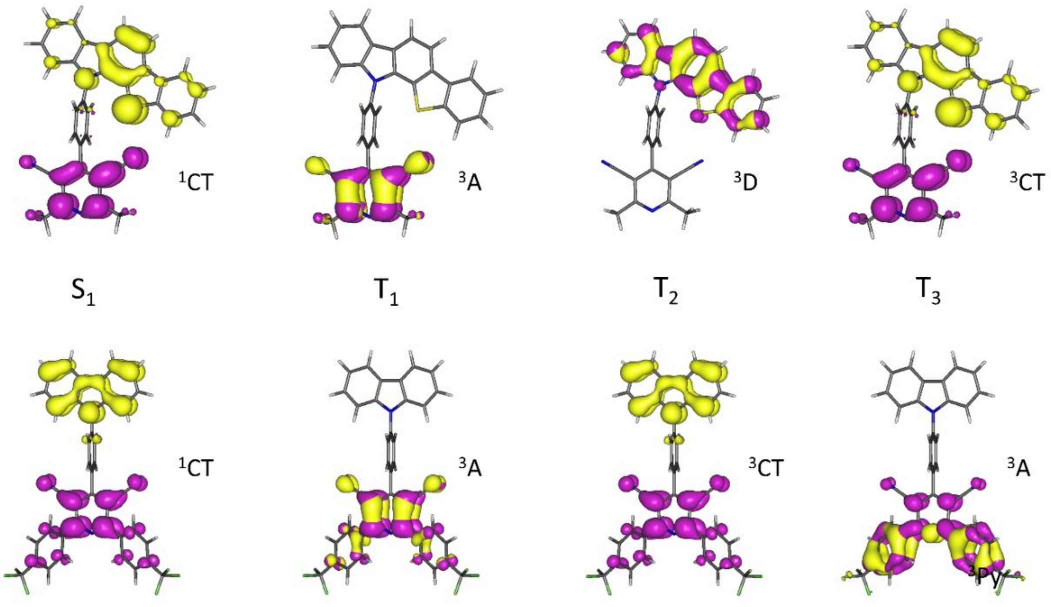

Figure S25. Natural transition orbitals (NTOs, yellow = hole, purple = particle) for selected states on optimized S_1 excited state geometries of **TCz-Me** (top) and **Cz-CF3** (bottom).

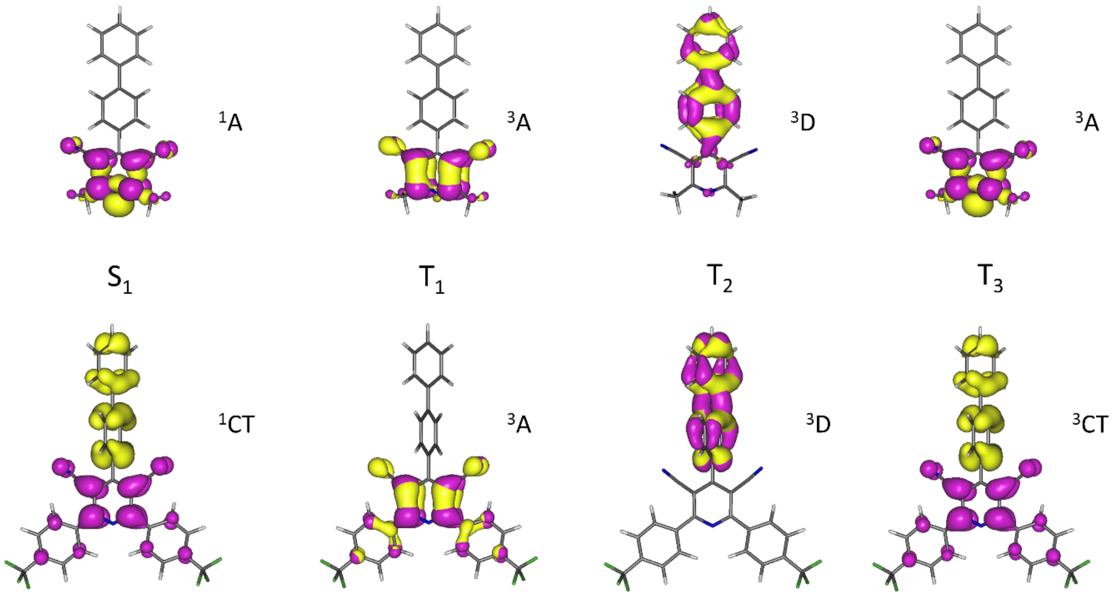

Figure S26. Natural transition orbitals for selected states on optimized S_1 excited state geometries of **PhPyMe** (top) and **PhPyCF3** (bottom).

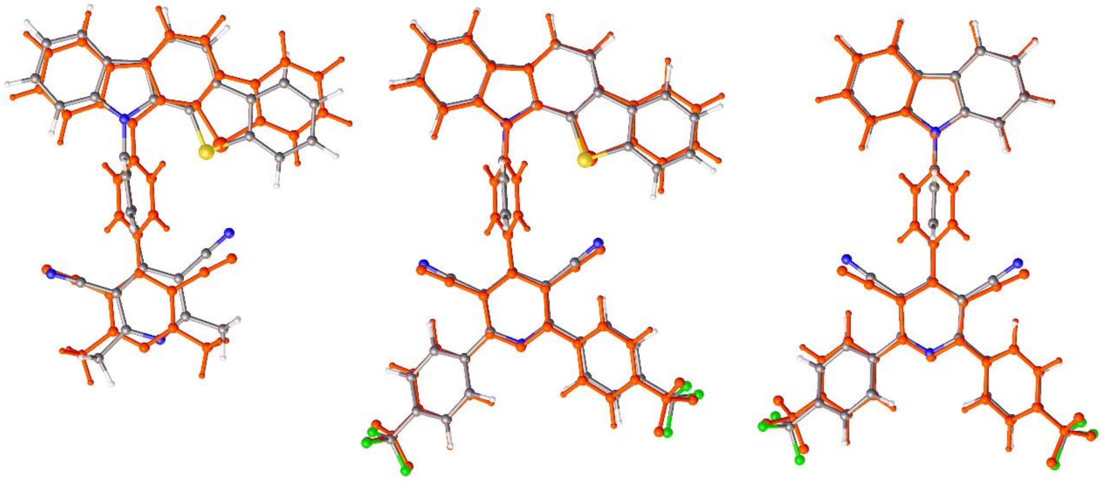

Figure S27. Geometry fittings between optimized S_1 and T_1 geometries for **TCz-Me**, **TCz-CF3** and **Cz-CF3**. The T_1 geometries are shown in orange. Misfit values in Å are 0.753, 0.493 and 0.595 respectively for all atoms.

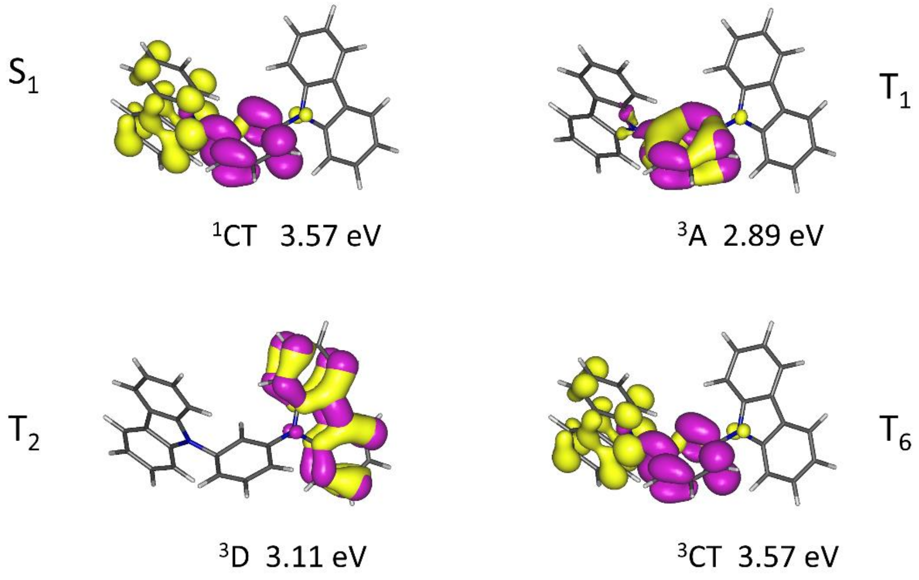

Figure S28. Natural transition orbitals and energies for selected states on the optimized S_1 excited state geometry of mCP with toluene as solvent.

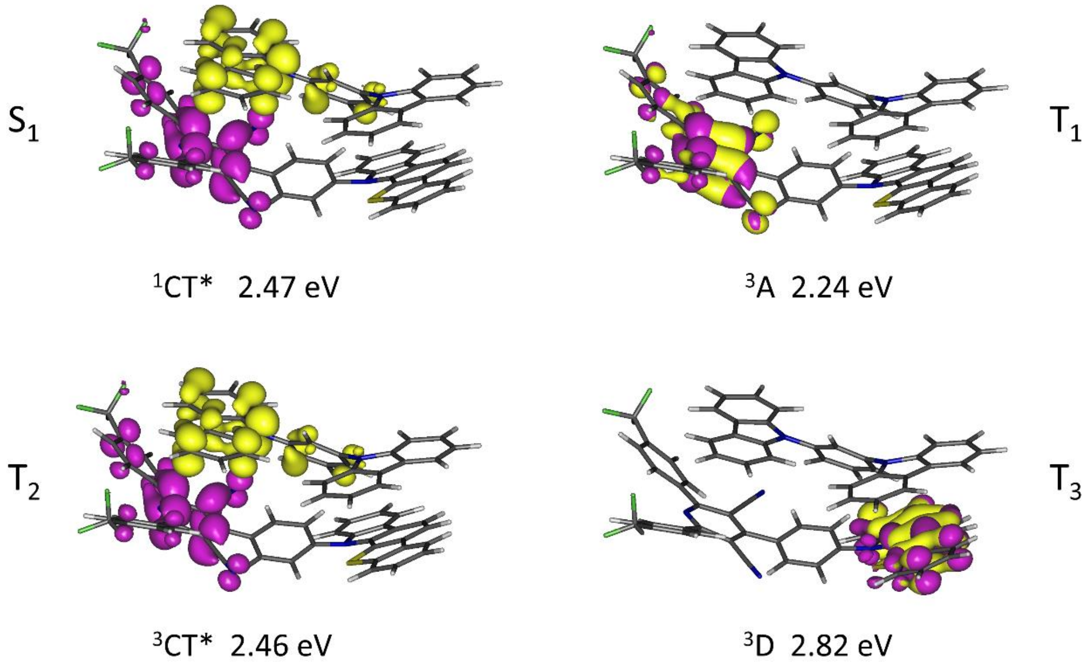

Figure S29. Natural transition orbitals and energies for lowest energy states on the optimized S_1 excited state geometry of **TCz-CF3:mCP** with toluene as solvent. CT* = intermolecular CT.

8. Electroluminescence Properties

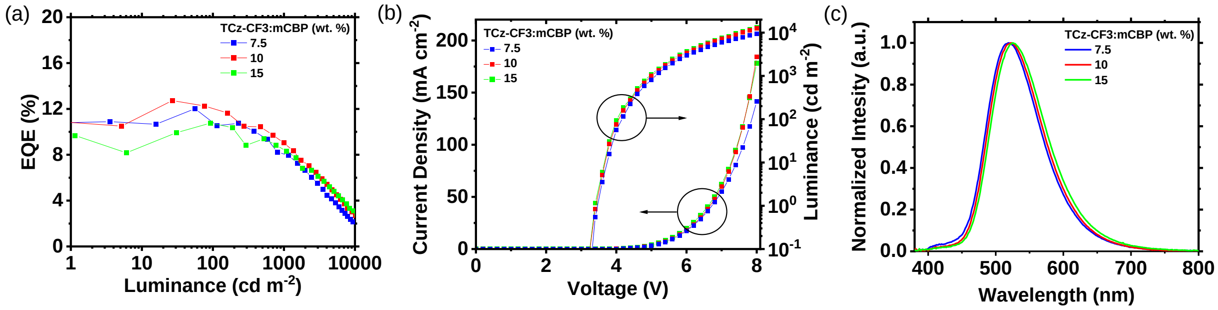

Figure S30. OLED performances of **TCz-CF3** in different doping ratios in mCBP host: (a) external quantum efficiency at different luminances; (b) current and luminances at different voltages and (c) EL spectra.

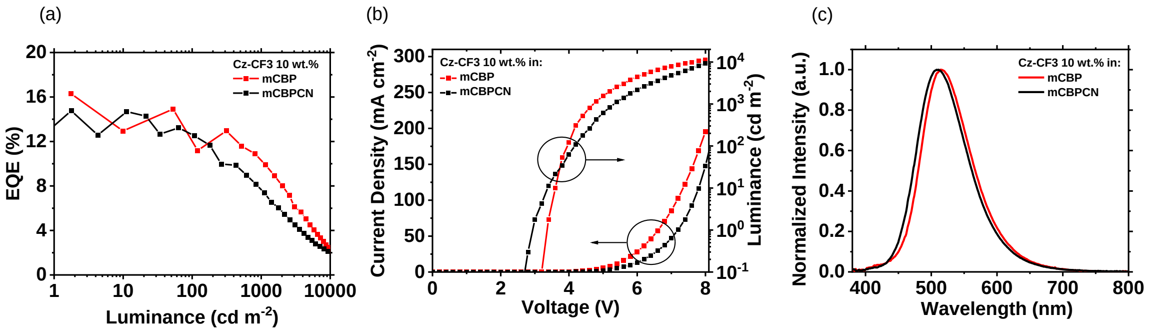

Figure S31. OLED performances of **Cz-CF3** at 10 wt. % in mCBP and mCBPCN hosts: (a) external quantum efficiency at different luminances; (b) current and luminances at different voltages and (c) EL spectra.

Table S11. Electroluminescence performance of the OLEDs.

Emitter / host[a]	λ_{EL} / nm [b]	V_{on} / V [c]	L_{max} / cd/m^2 [d]	CE_{max} / cd/A[e]	PE_{max} / lm/W[f]	EQE$_{max}$/100/500/ 1000 /% [g]	FWHM/ nm[h]	CIE$_{(x,y)}$ [i]
**TCzCF3** /mCBP	523	3.25	12484	34.5	27.1	12.7/12.1/10.3/9	92	(0.3, 0.54)
**TCzCF3** /DPEPO	532	4	778	20.6	12.4	5.2/3.6/1/-	113	(0.31, 0.5)
**CzCF3** /mCBP	515	3.2	11413	37.1	30.7	16.3/13/11.8/10.3	87	(0.27, 0.53)
**CzCF3** /DPEPO	513	4	510	11	7.6	3.3/2.7/0.6/-	136	(0.28, 0.42)
**CzCF3** /mCBPCN	510	2.8	9300	37.6	29.5	14.8/12.6/9.6/7.7	88	(0.25, 0.5)

[a] 10 wt. % emitter; [b] EL peak wavelength; [c] Turn-on voltage at 0.1 cd/m^2; [d] Maximum luminance; [e] Maximum current efficiency; [f] Maximum power efficiency; [g] External quantum efficiency maximum and values at 100, 500, 1000 cd/m^2; [h] Full-width at half-maximum; [i] Commission Internationale de l'Eclairage coordinates, values taken at 8 V.

9. Characterizations

9.1. NMR Spectra

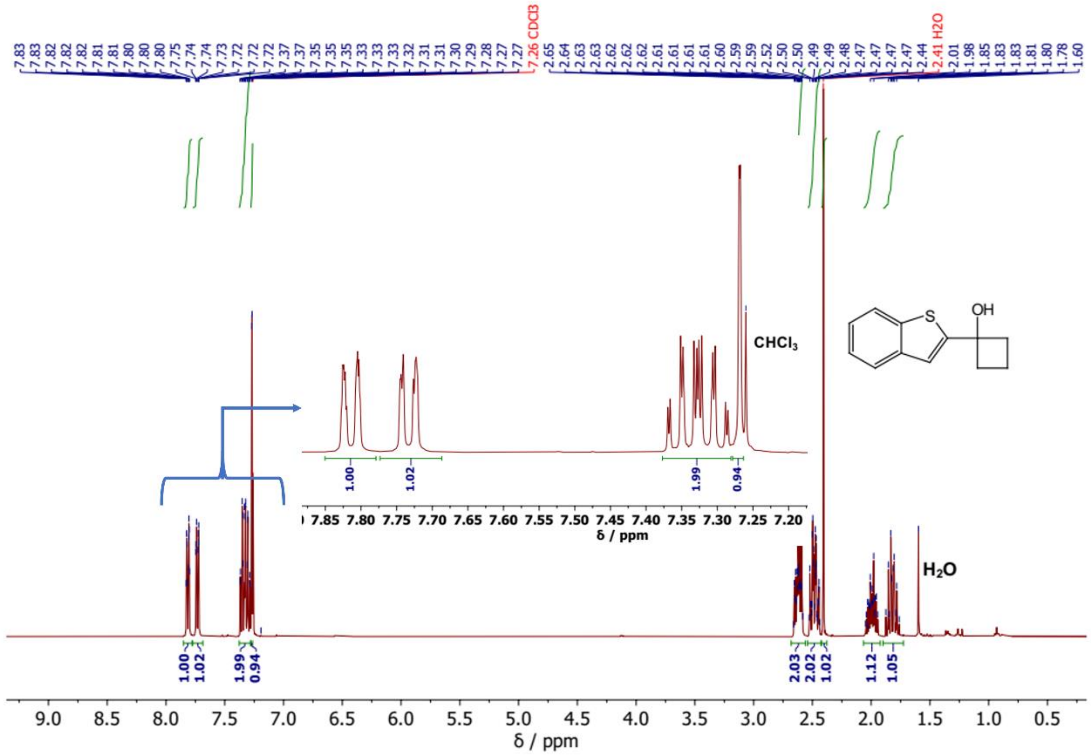

Figure S32. ^{1}H-NMR spectrum of **1** in $CDCl_3$.

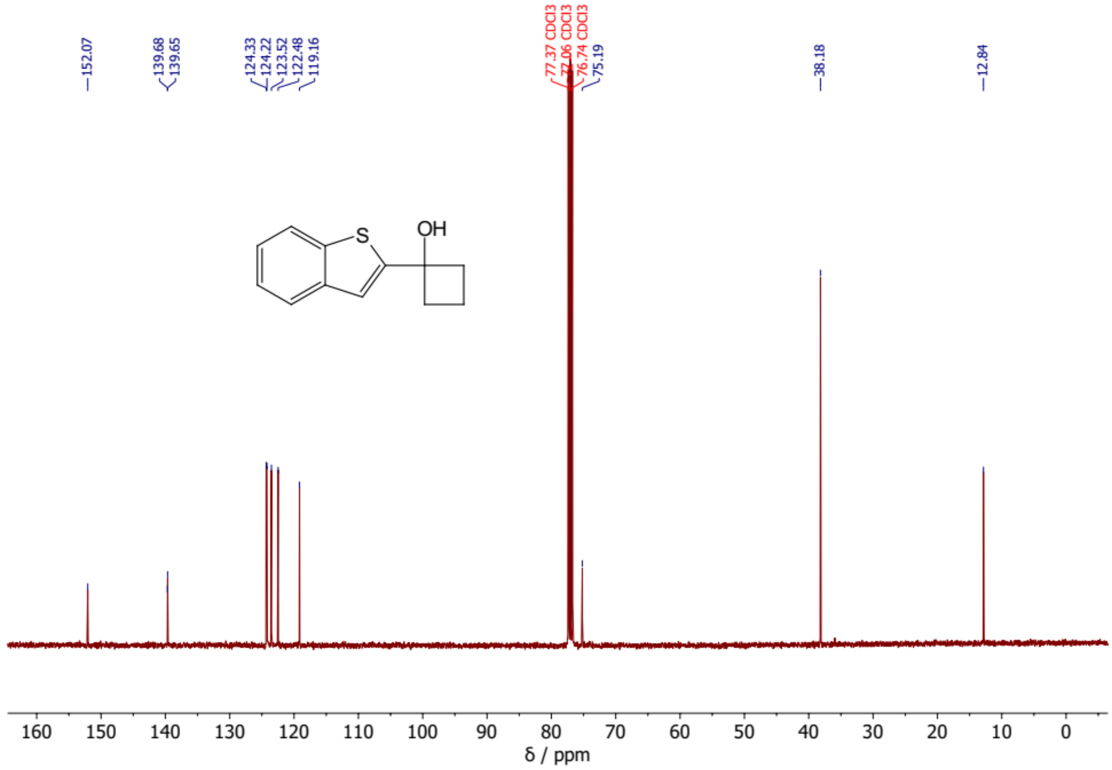

Figure S33. $^{13}C\{^{1}H\}$-NMR spectrum of **1** in $CDCl_3$.

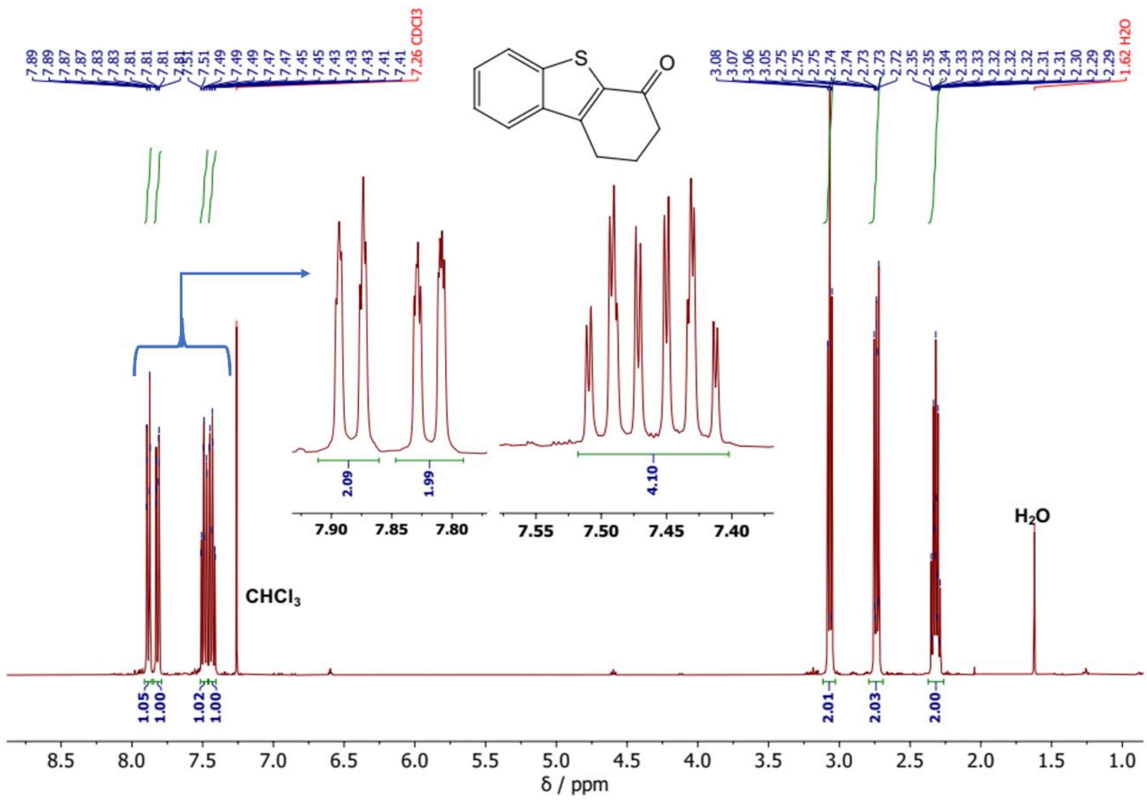

Figure S34. ^{1}H-NMR spectrum of **2** in $CDCl_3$.

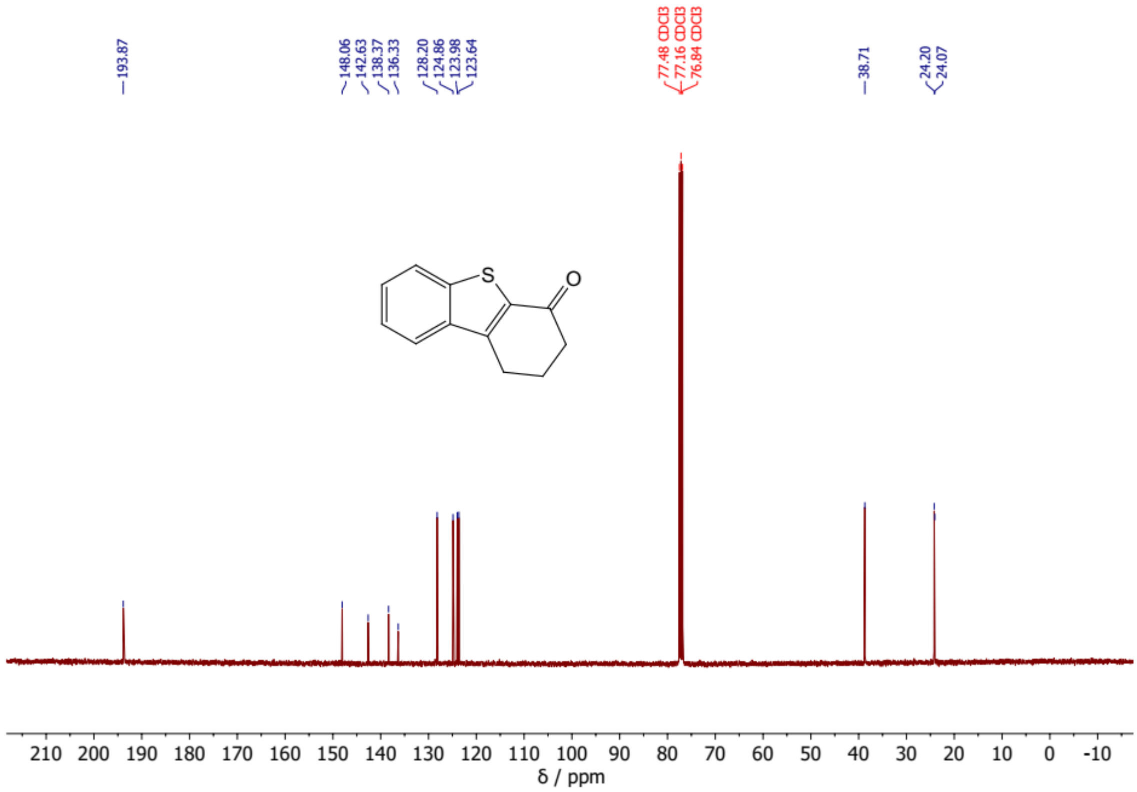

Figure S35. ^{13}C{^{1}H}-NMR spectrum of **2** in $CDCl_3$.

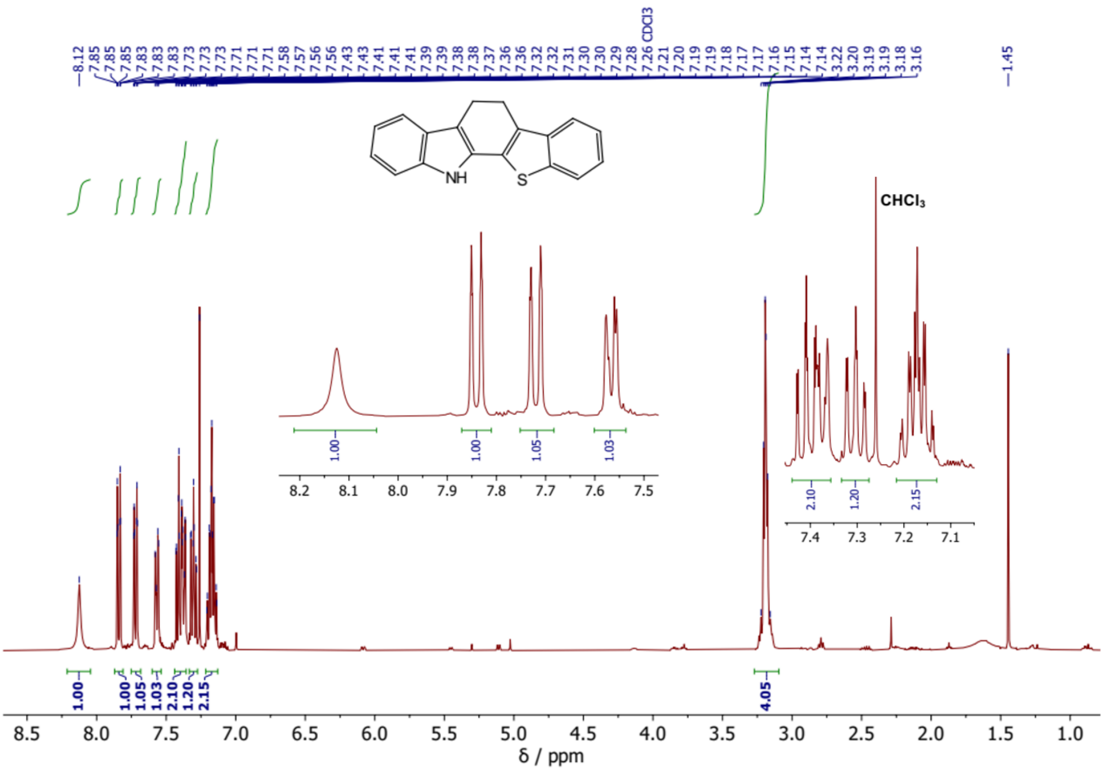

Figure S36. ^{1}H-NMR spectrum **of 3a** in $CDCl_3$.

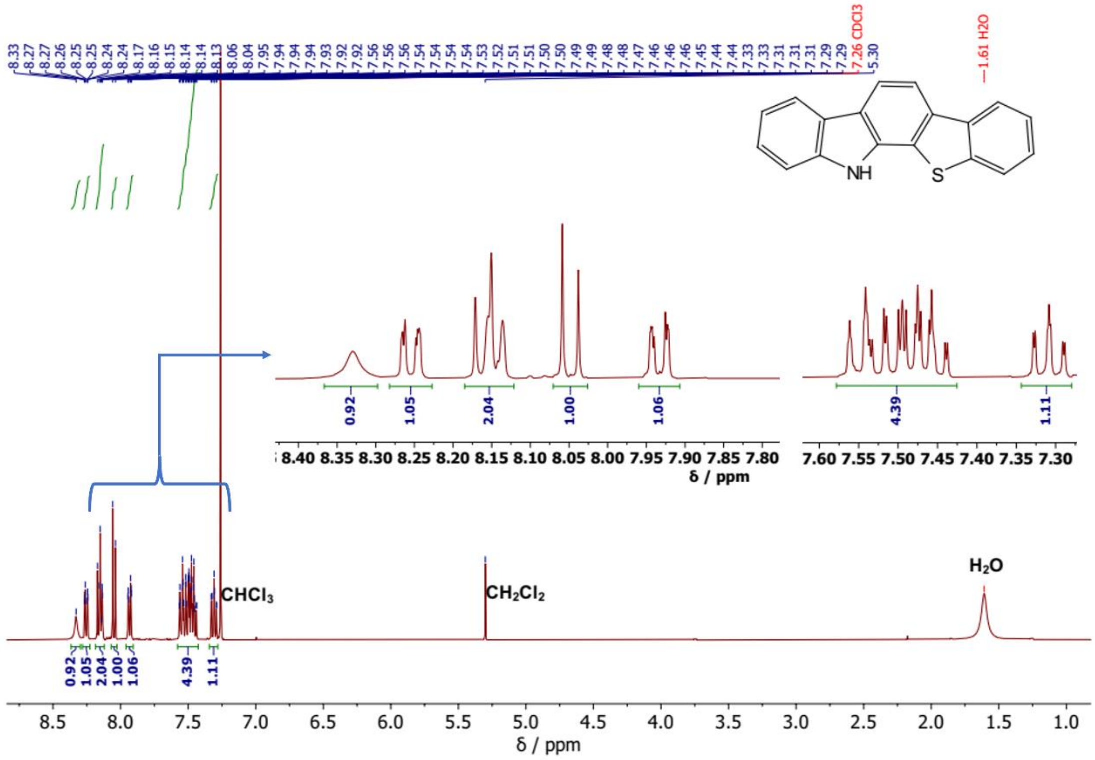

Figure S37. ^{1}H-NMR spectrum of **3** in $CDCl_3$.

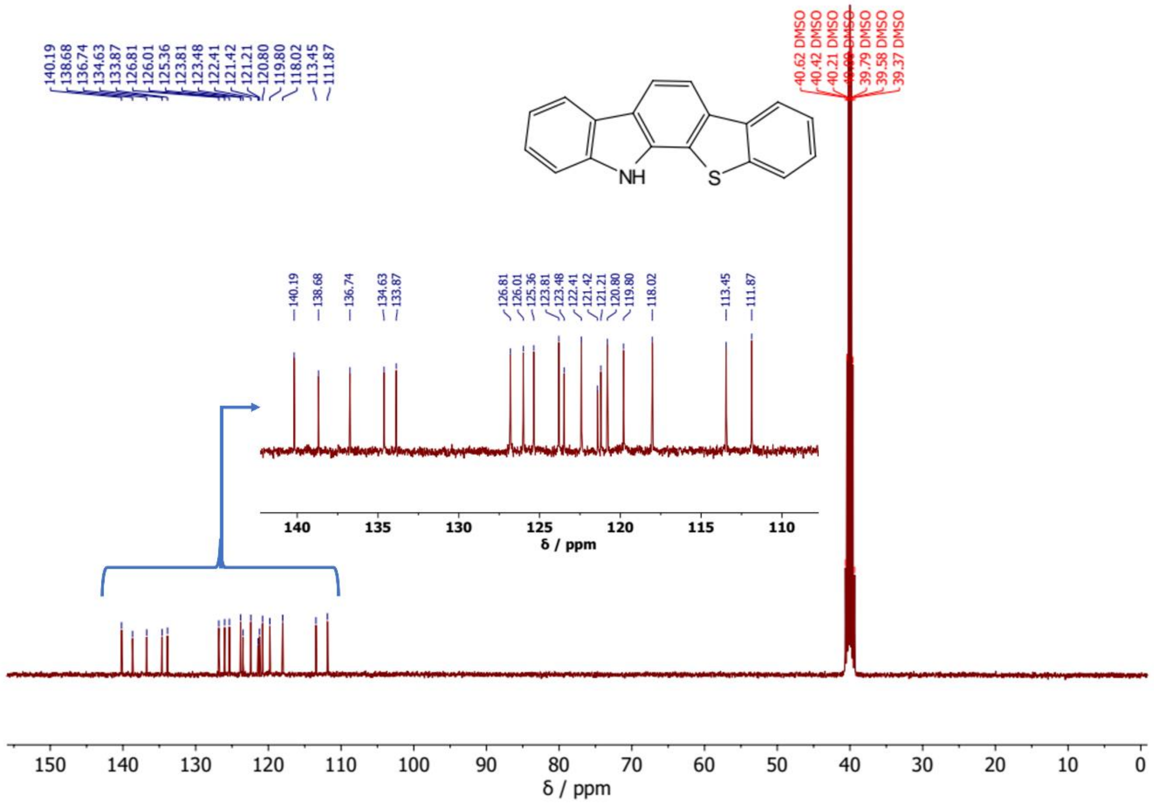

Figure S38. $^{13}C\{^{1}H\}$-NMR spectrum of **3** in d_6-DMSO.

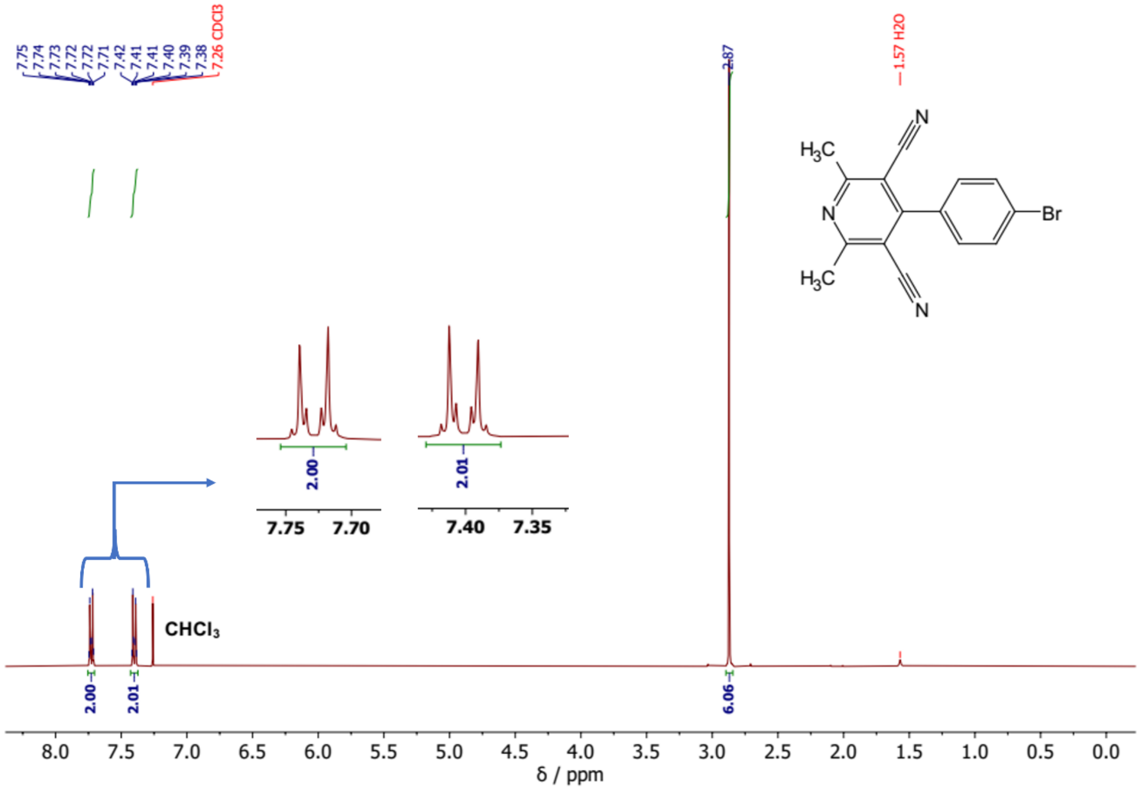

Figure S39. ^{1}H-NMR spectrum of **4** in $CDCl_3$.

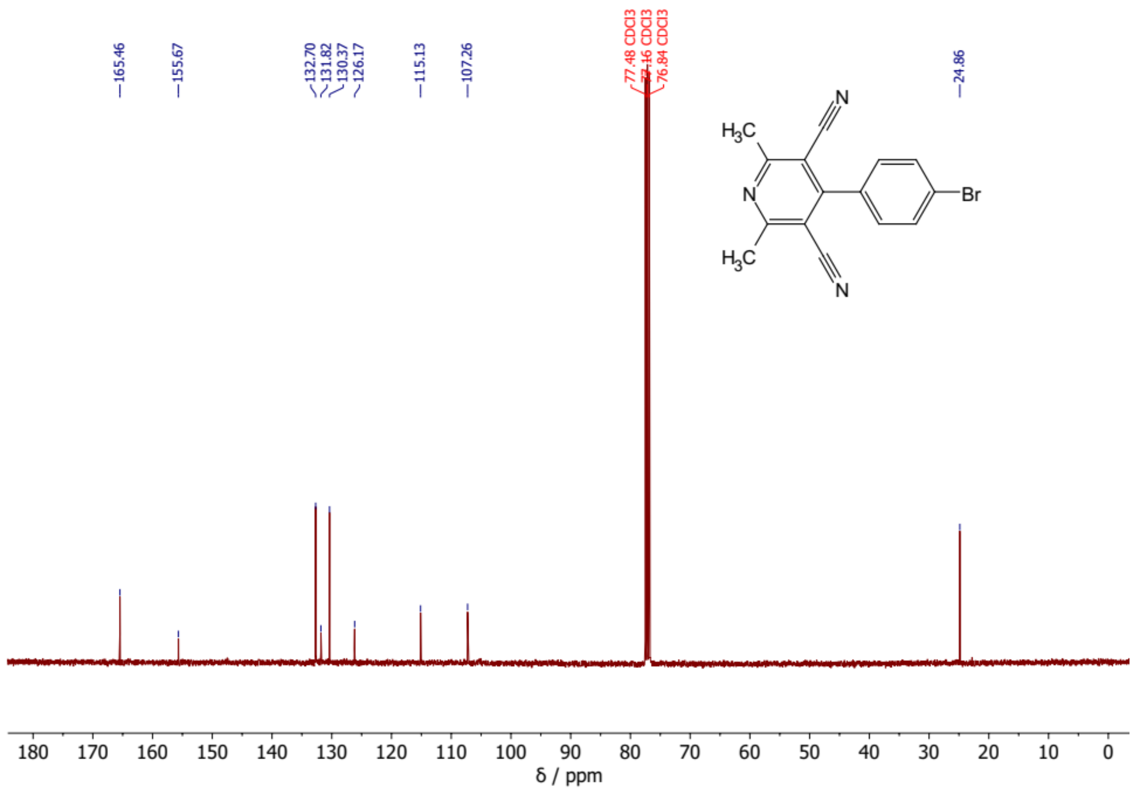

Figure S40. $^{13}C\{^{1}H\}$-NMR spectrum of **4** in $CDCl_3$.

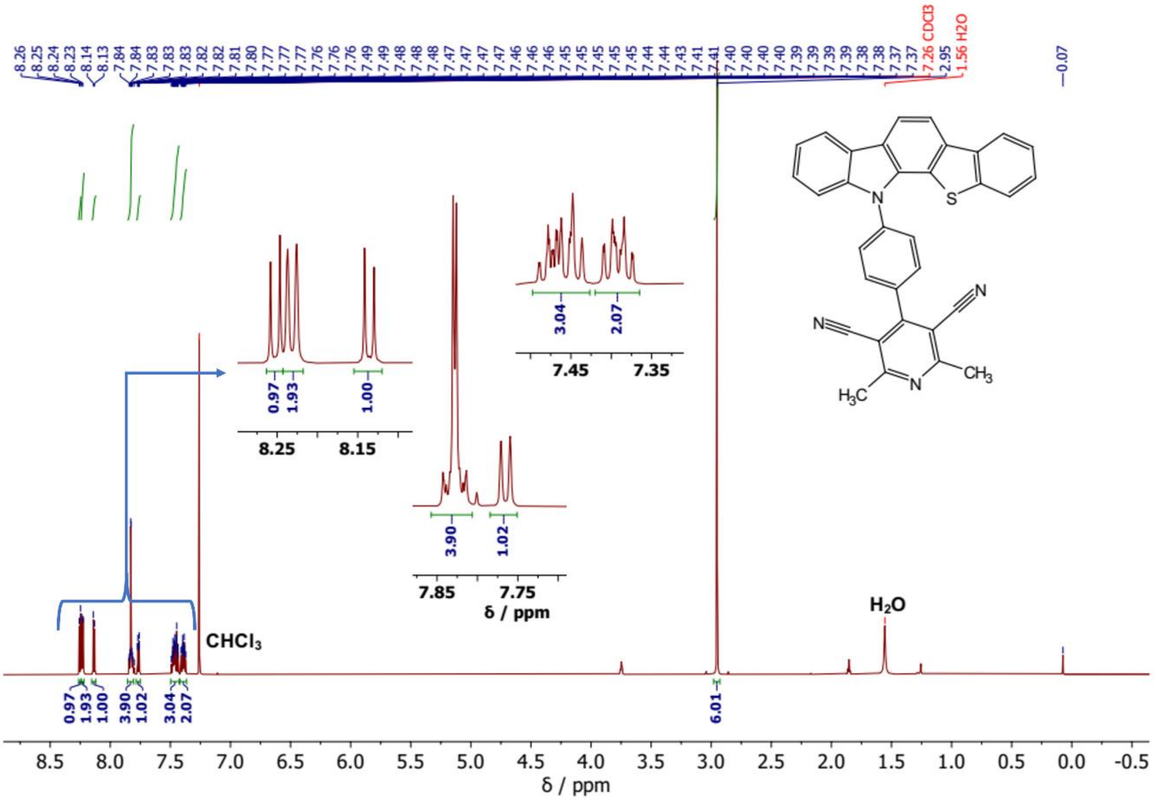

Figure S41. ^{1}H-NMR spectrum of **TCz-Me** in $CDCl_3$.

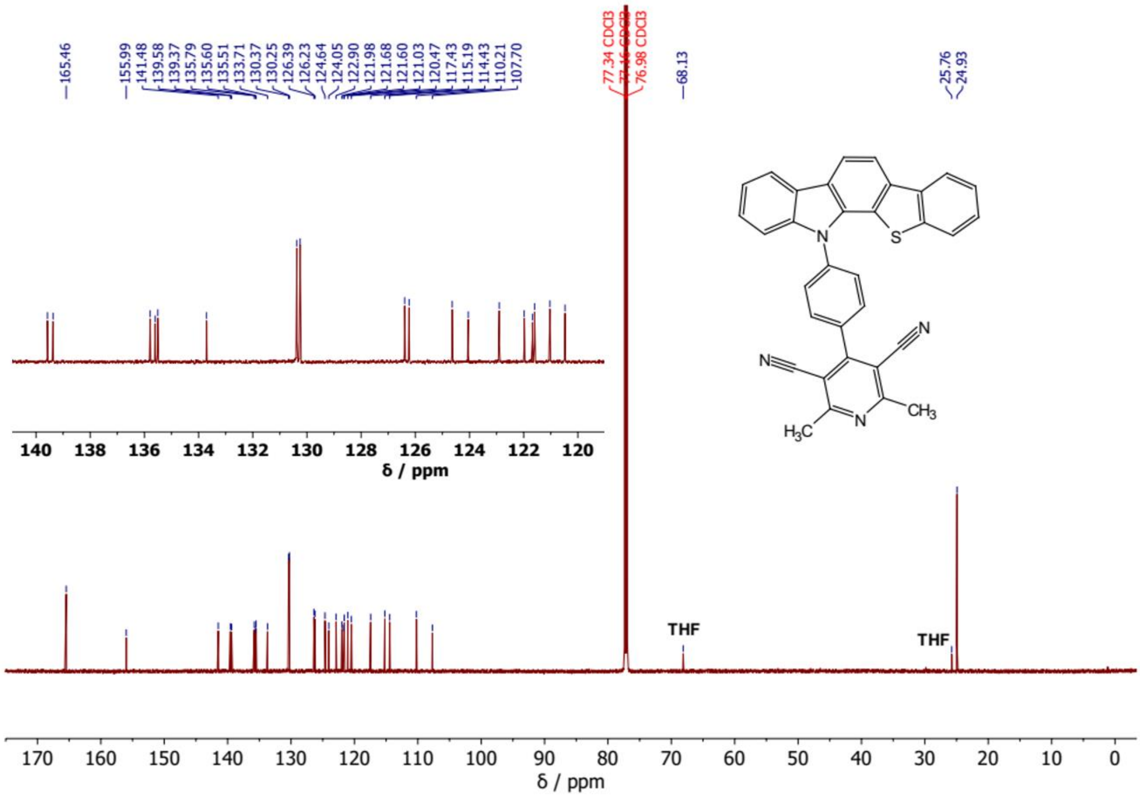

Figure S42. $^{13}C\{^{1}H\}$-NMR spectrum of **TCz-Me** in $CDCl_3$.

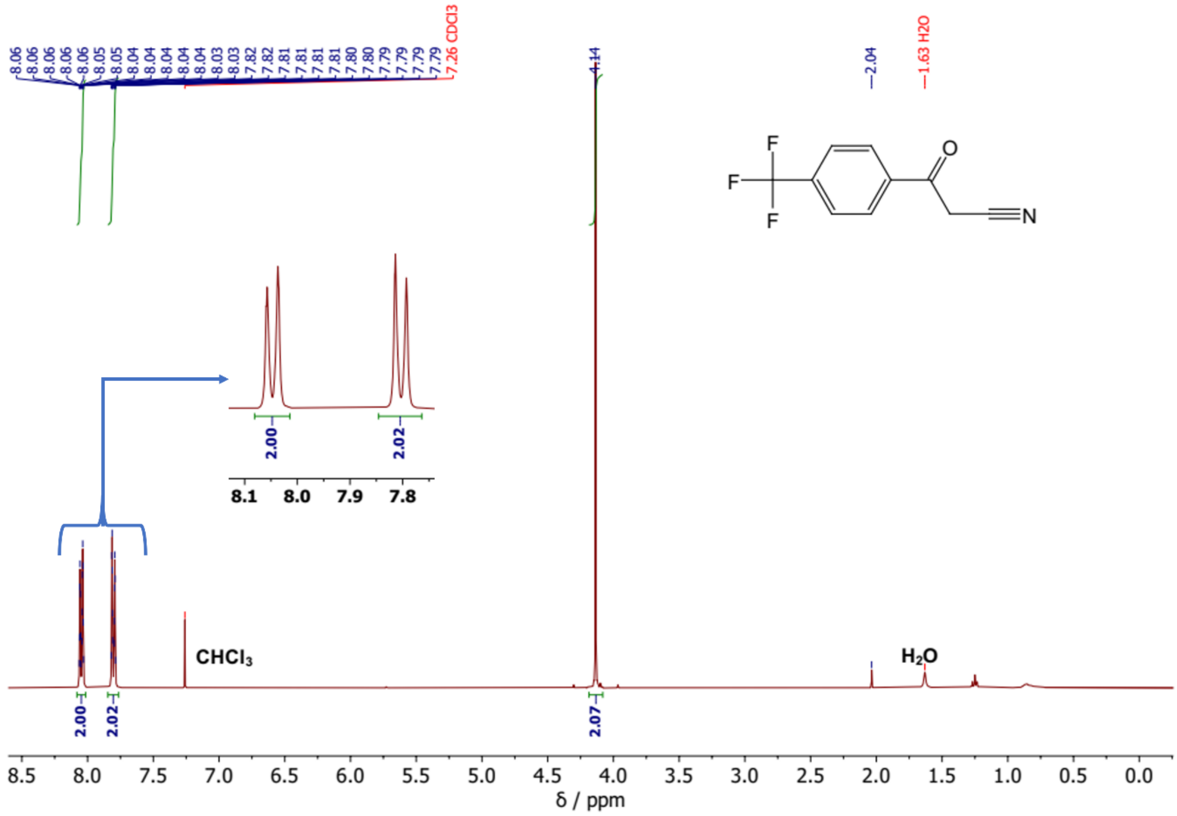

Figure S43. ^{1}H-NMR spectrum of **5** in $CDCl_3$.

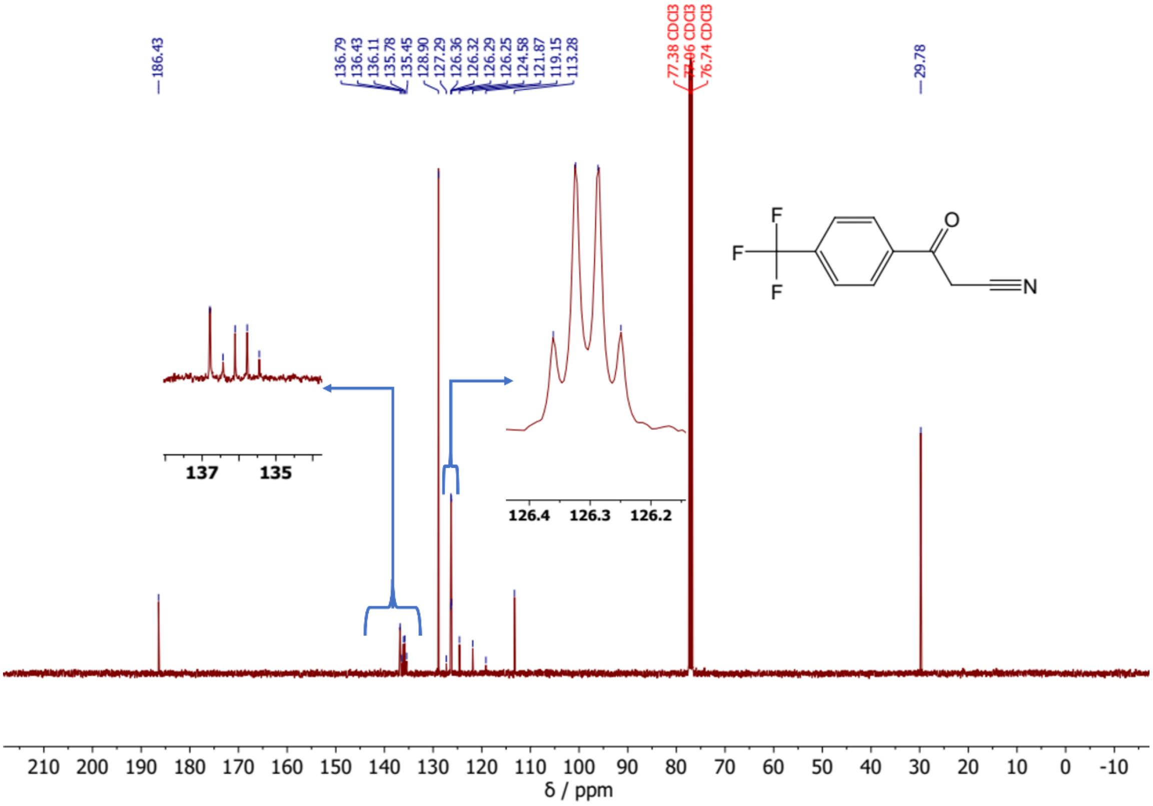

Figure S44. $^{13}C\{^{1}H\}$-NMR spectrum of **5** in $CDCl_3$.

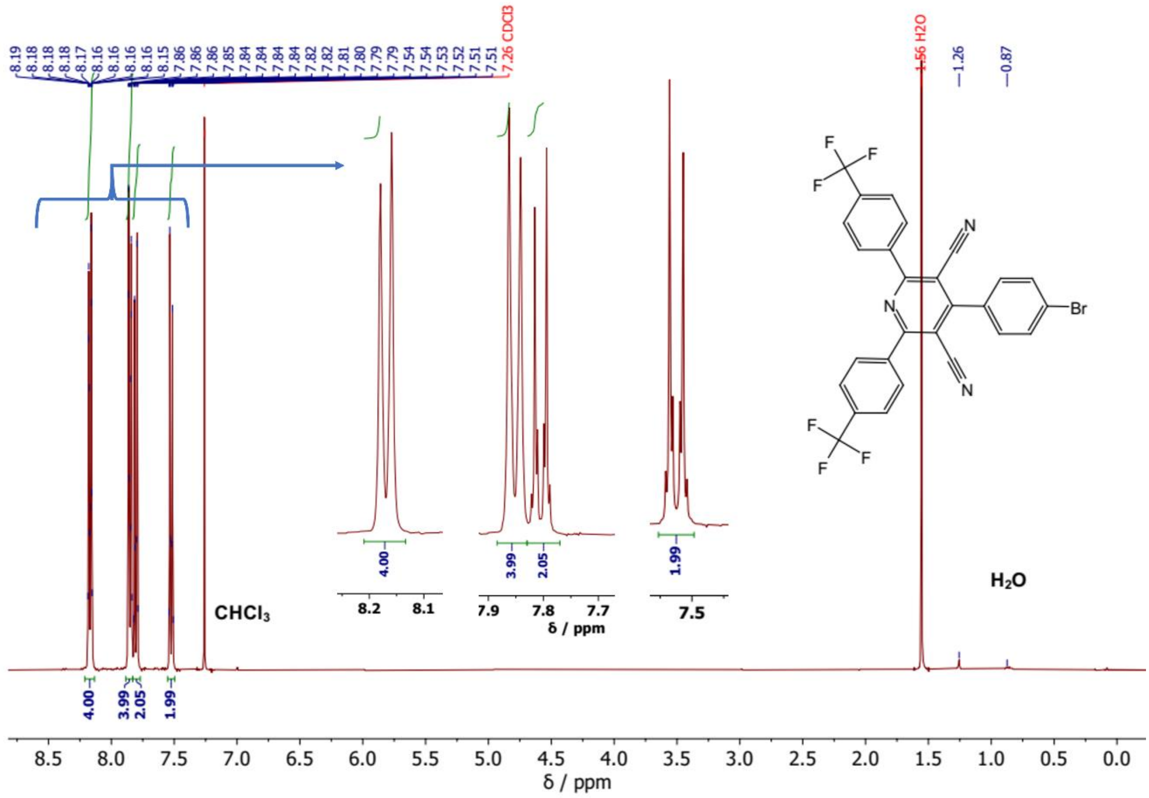

Figure S45. ^{1}H-NMR spectrum of **6** in $CDCl_3$.

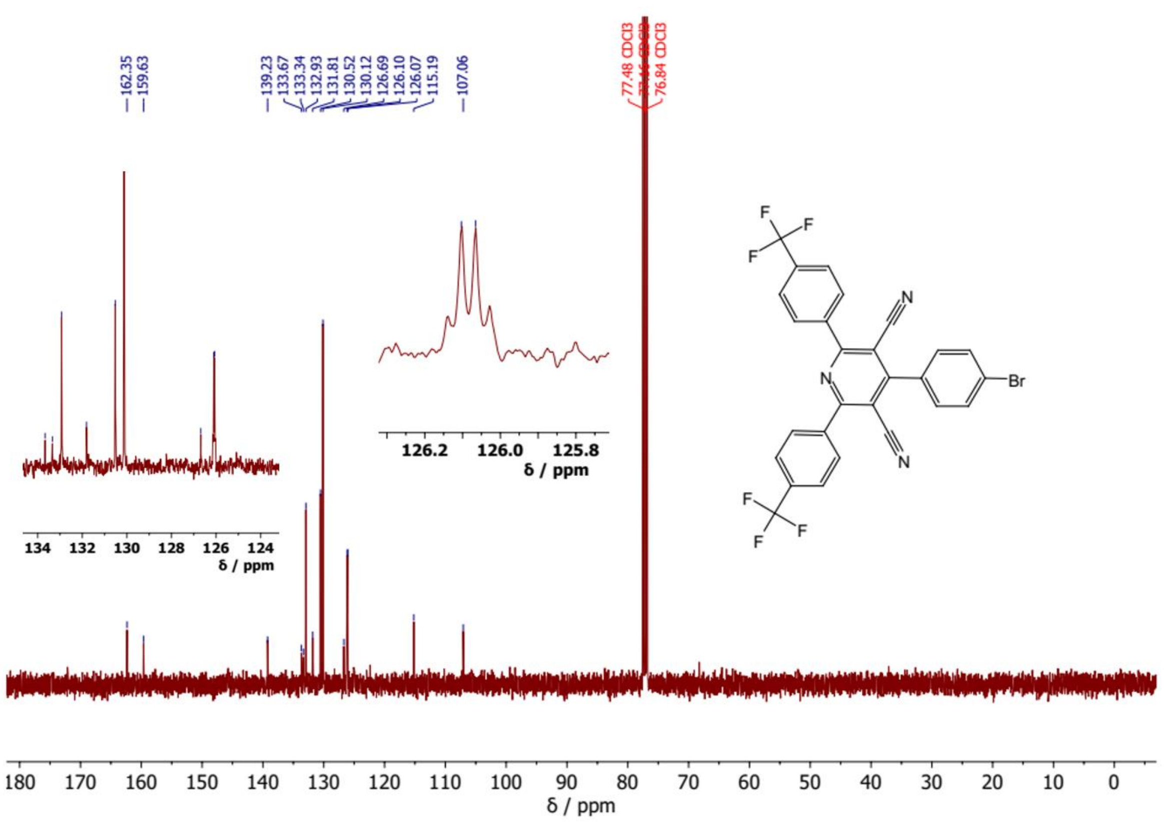

Figure S46. $^{13}C\{^1H\}$-NMR spectrum of **6** in $CDCl_3$.

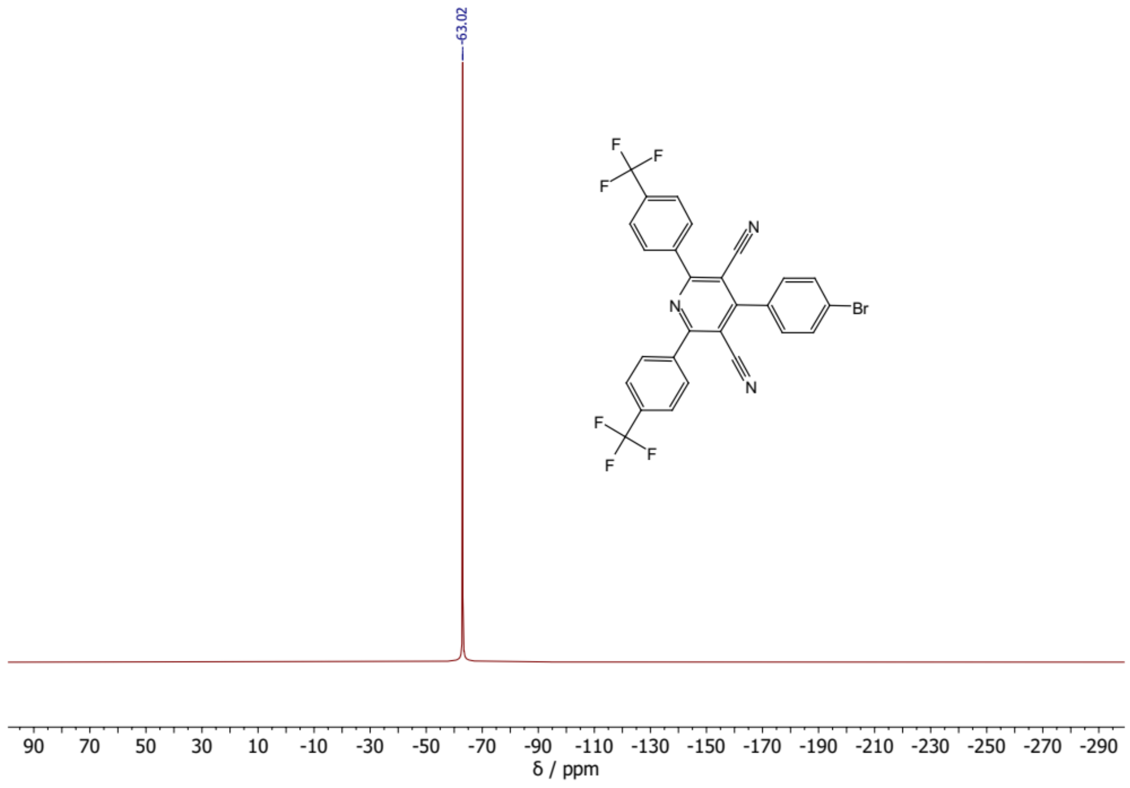

Figure S47. $^{19}F\{^1H\}$-NMR spectrum of **6** in $CDCl_3$.

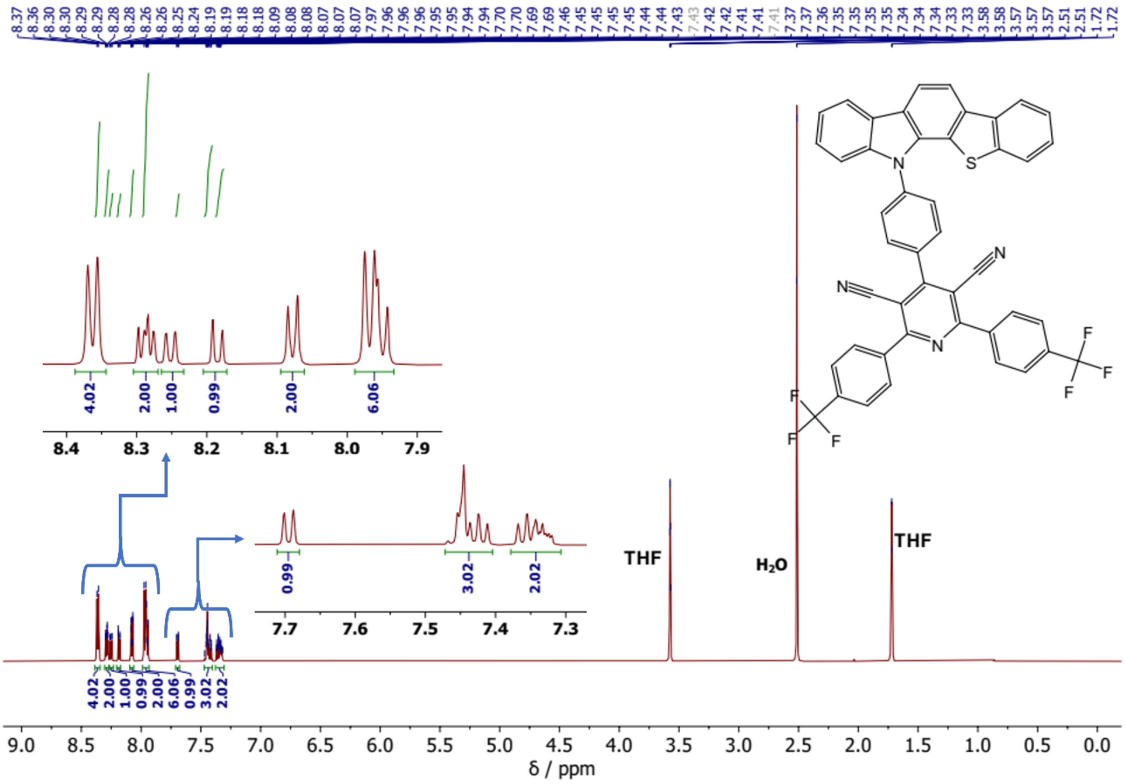

Figure S48. ^{1}H-NMR spectrum of **TCz-CF3** in THF-d_8.

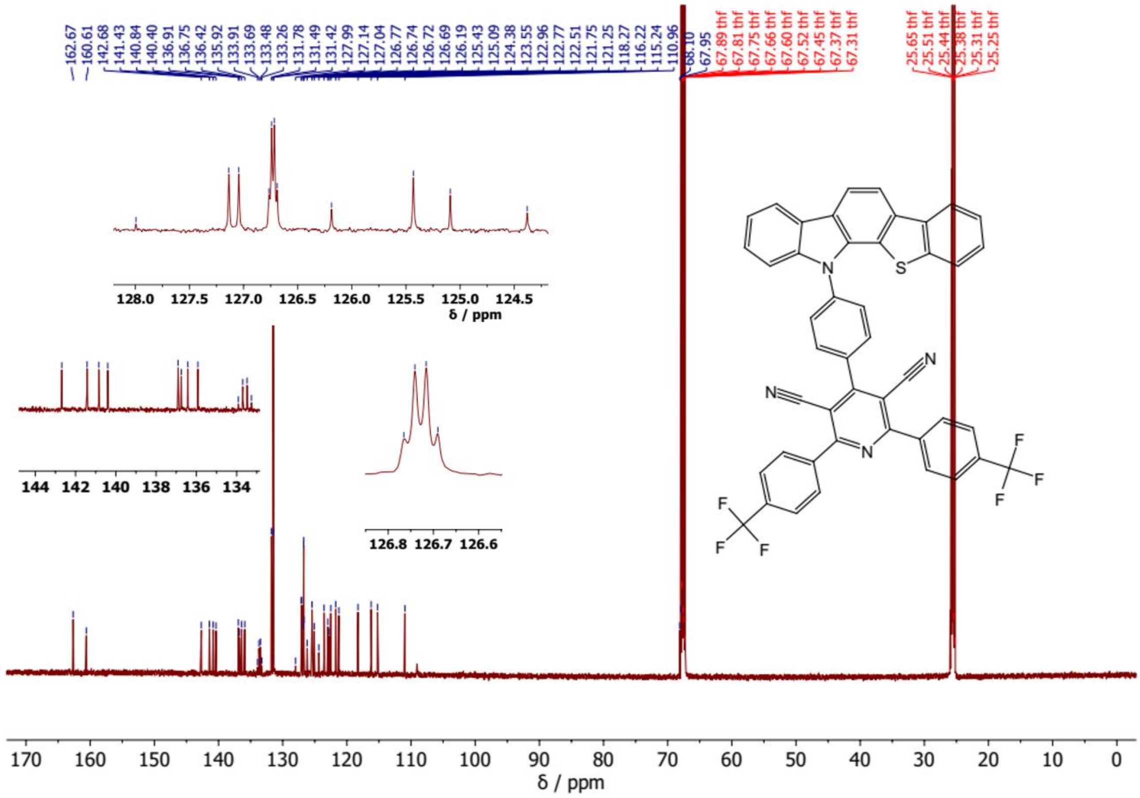

Figure S49. $^{13}C\{^{1}H\}$-NMR spectrum of **TCz-CF3** in THF-d_8.

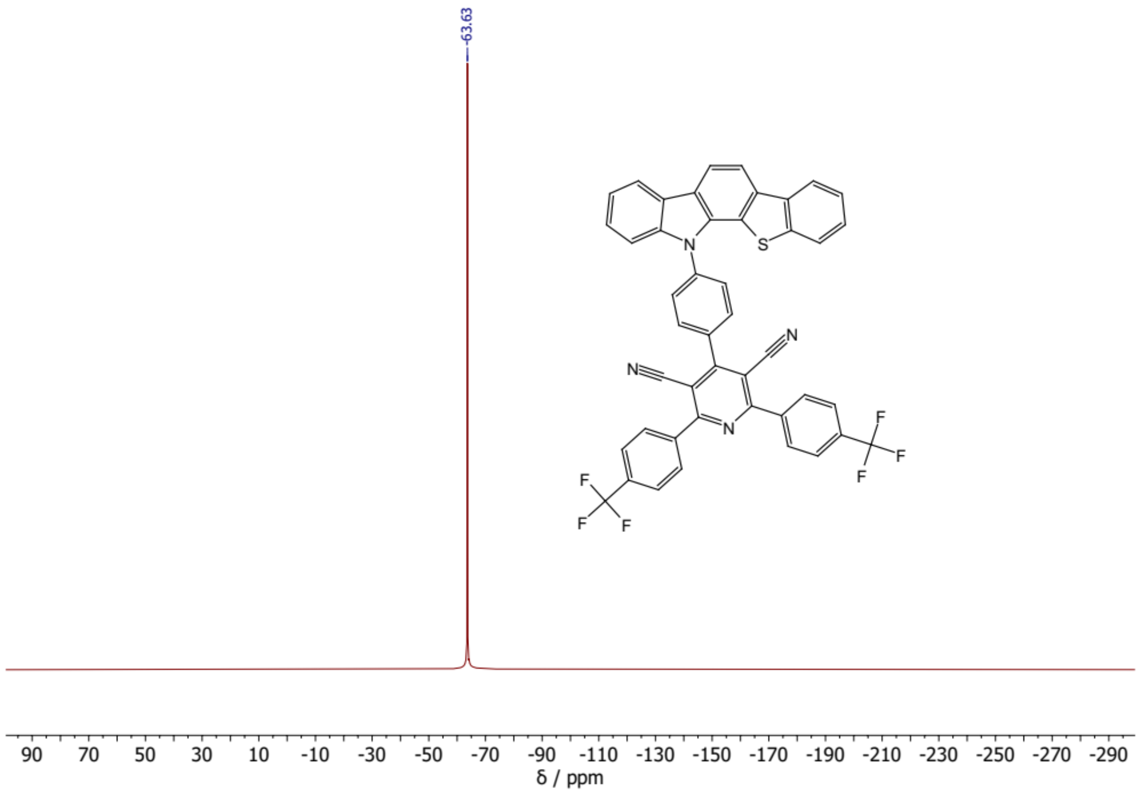

Figure S50. $^{19}F\{^{1}H\}$-NMR spectrum of **TCz-CF3** in THF-d_8.

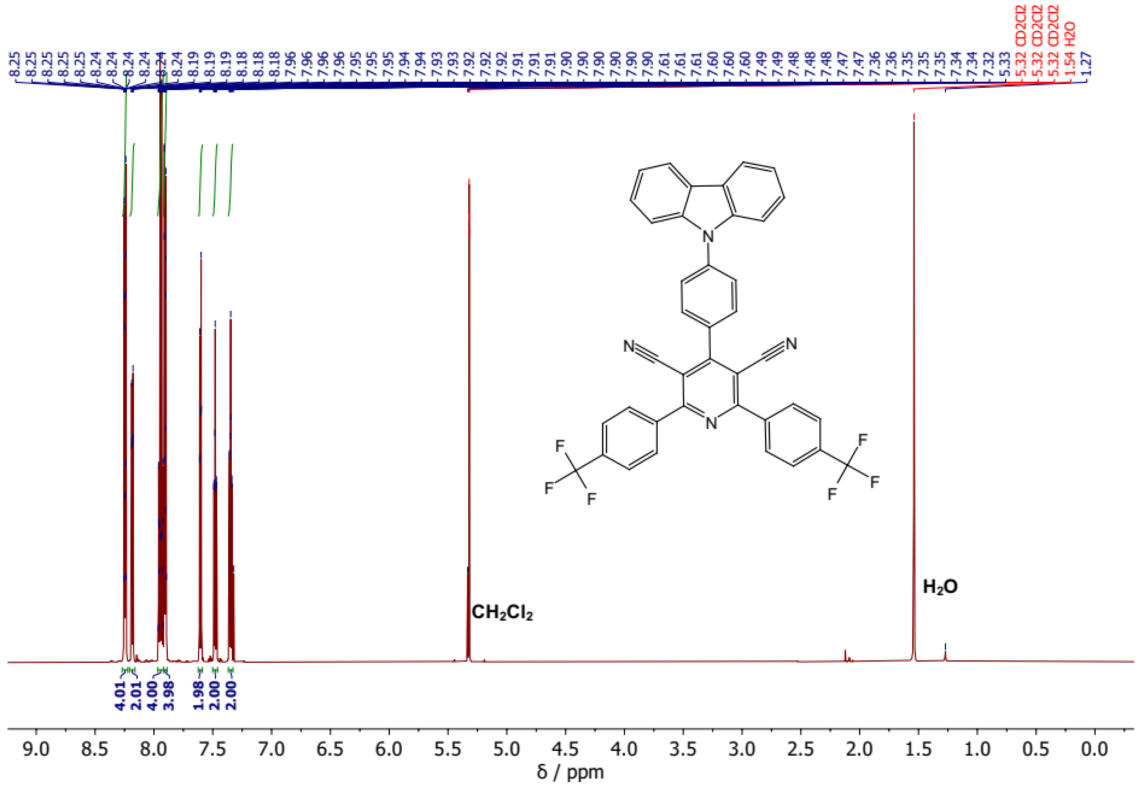

Figure S51. ^{1}H-NMR spectrum of **Cz-CF3** in CD_2Cl_2.

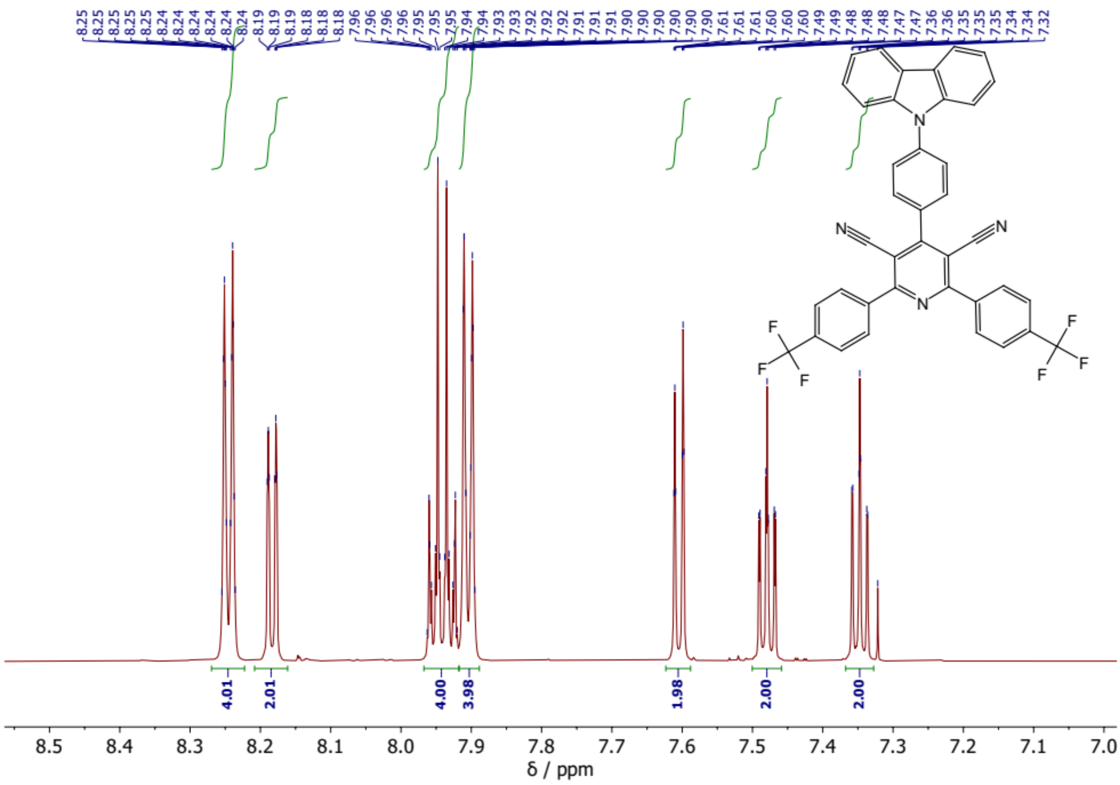

Figure S52. ^{1}H-NMR spectrum (aromatic region zoomed) of **Cz-CF3** in CD_2Cl_2.

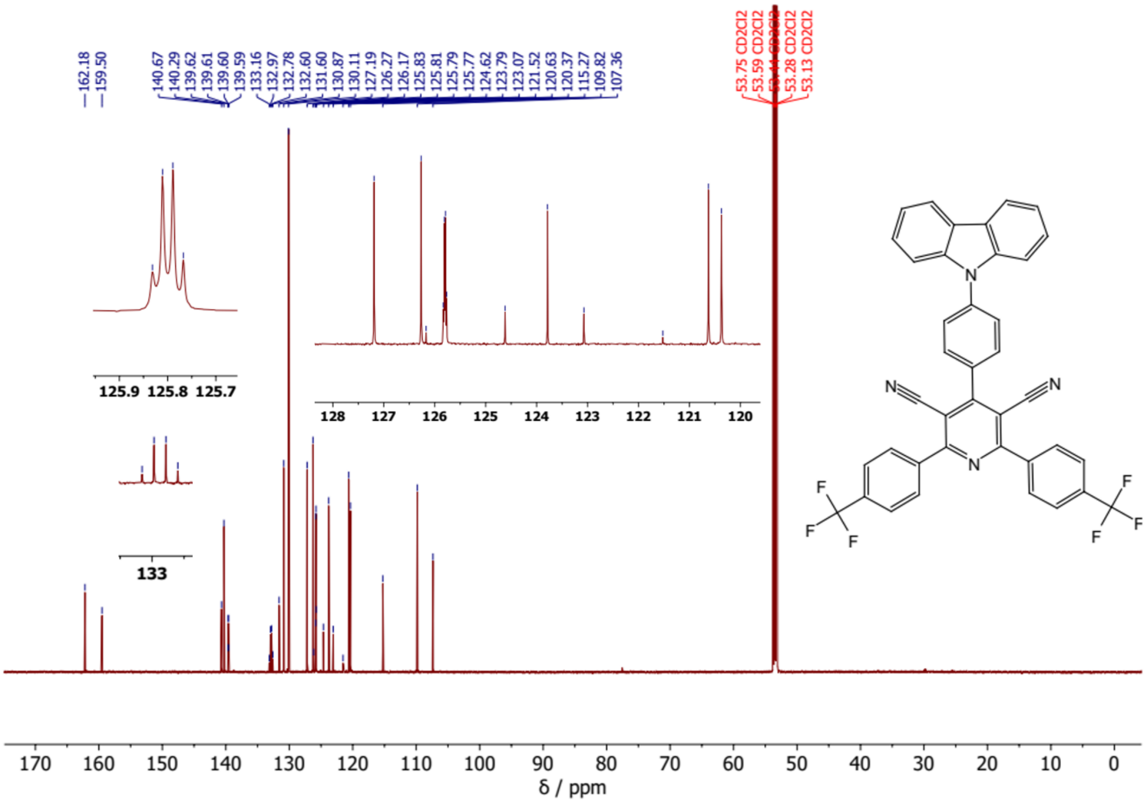

Figure S53. $^{13}C\{^{1}H\}$-NMR spectrum of **Cz-CF3** in CD_2Cl_2.

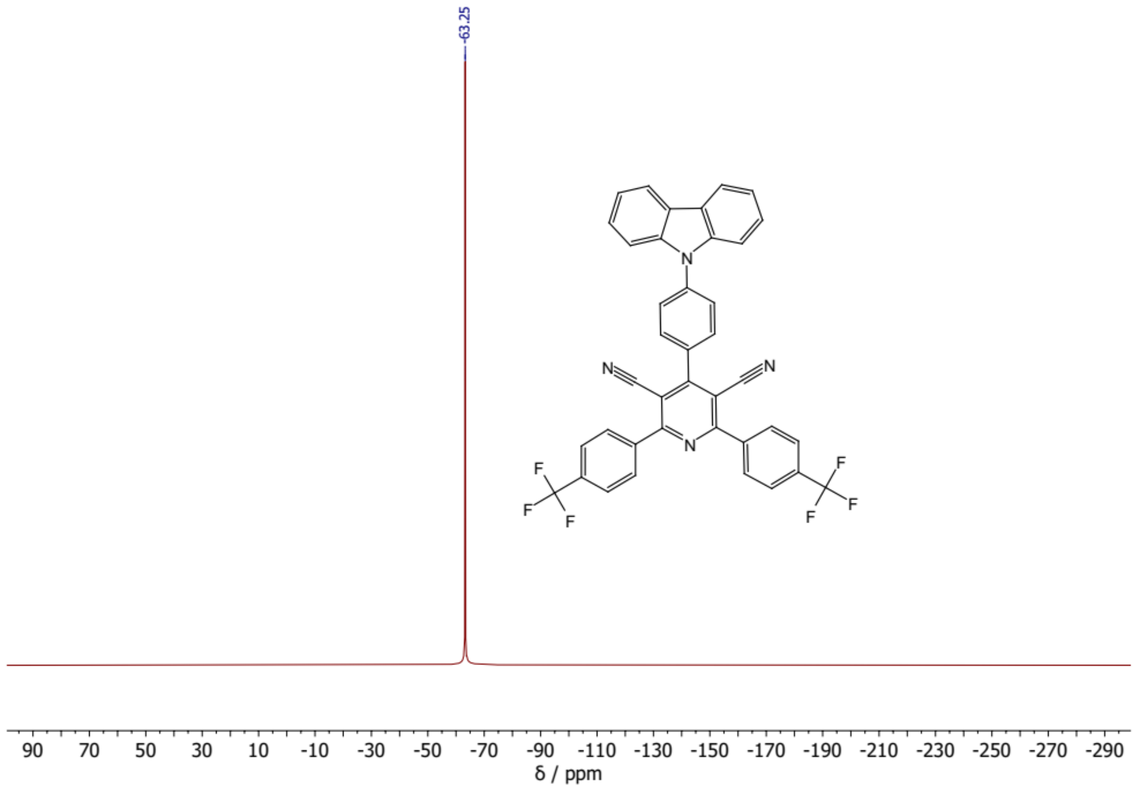

Figure S54. $^{19}F\{^{1}H\}$-NMR spectrum of **Cz-CF3** in CD_2Cl_2.

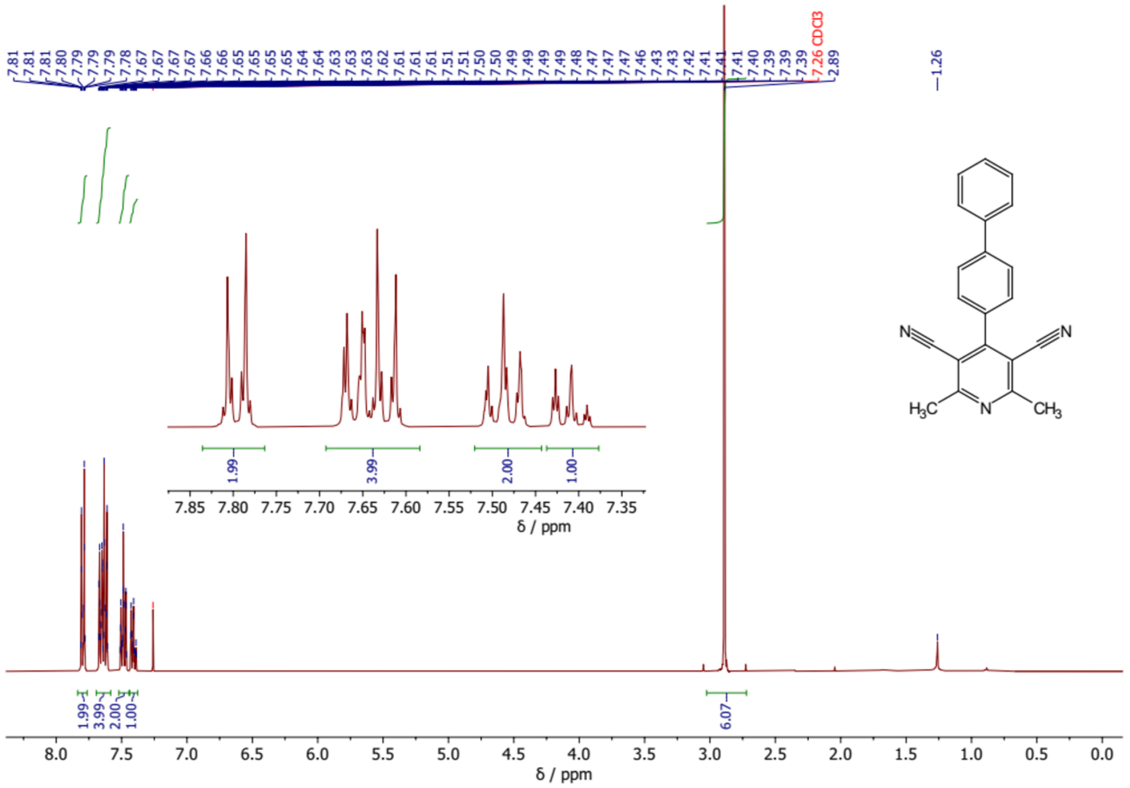

Figure S55. ^{1}H-NMR spectrum of **PhPyMe** in $CDCl_3$.

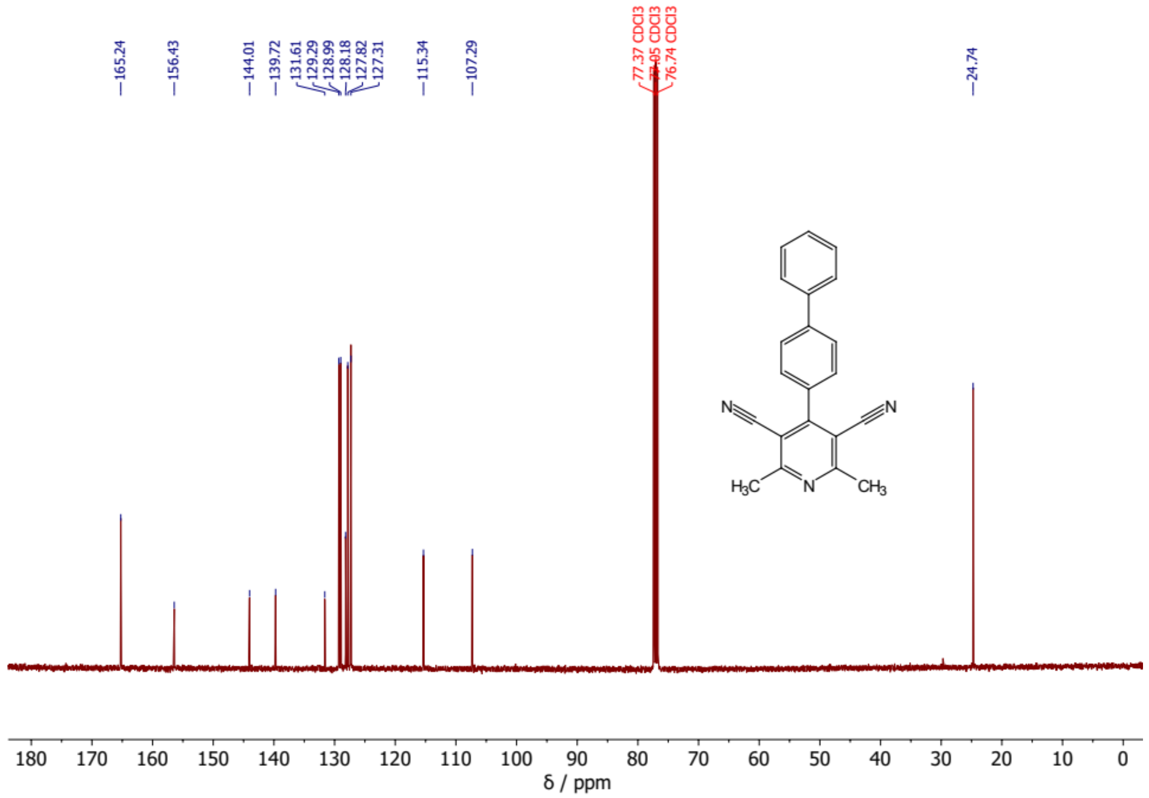

Figure S56. $^{13}C\{^{1}H\}$-NMR spectrum of **PhPyMe** in $CDCl_3$.

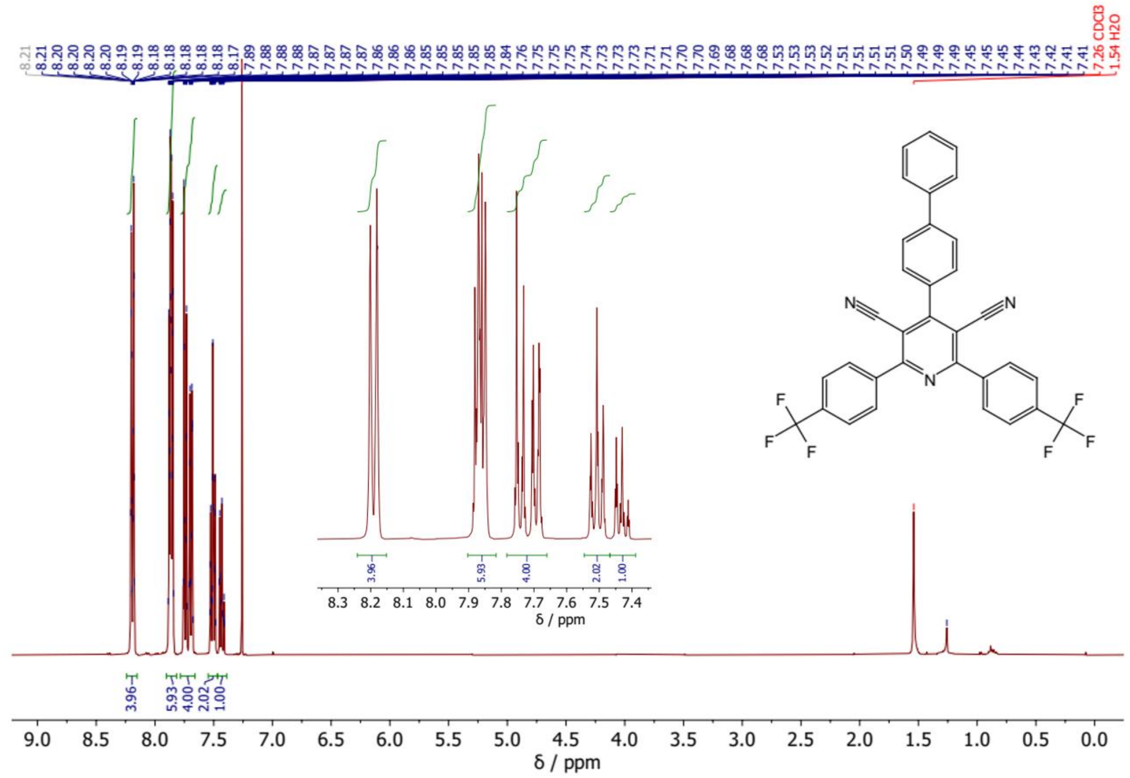

Figure S57. ^{1}H-NMR spectrum of **PhPyCF3** in $CDCl_3$.

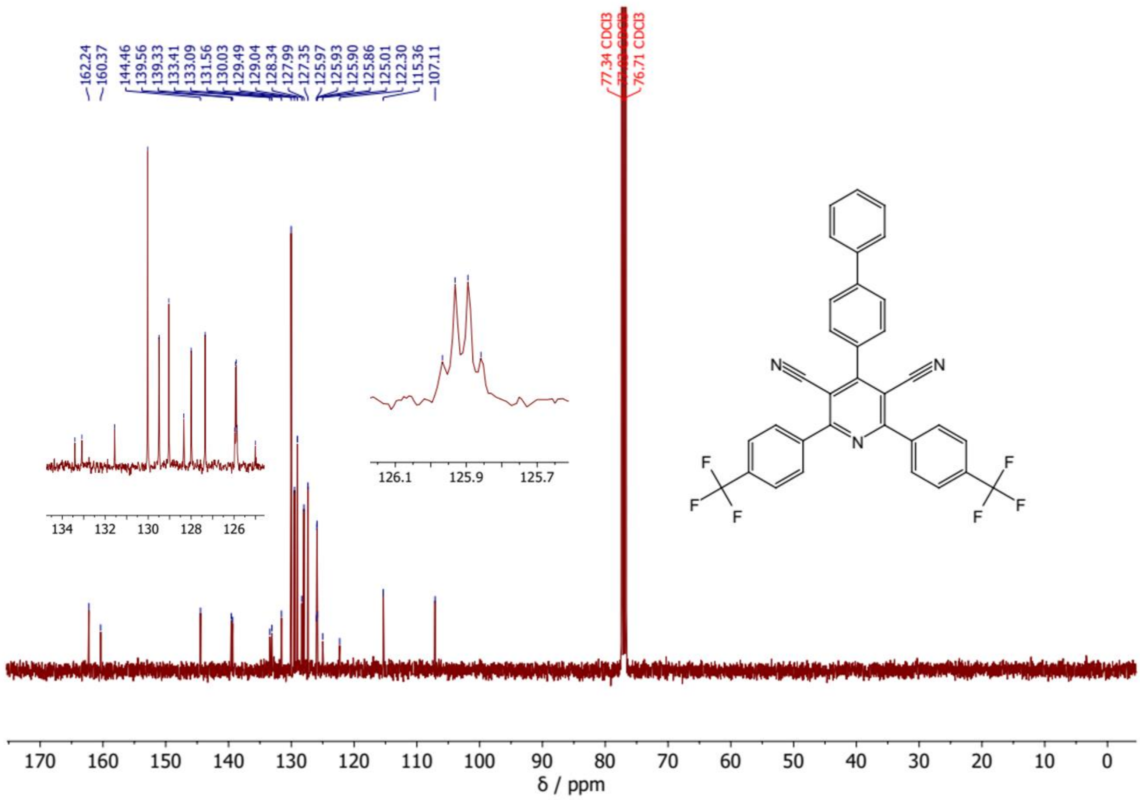

Figure S58. $^{13}C\{^1H\}$-NMR spectrum of **PhPyCF3** in $CDCl_3$.

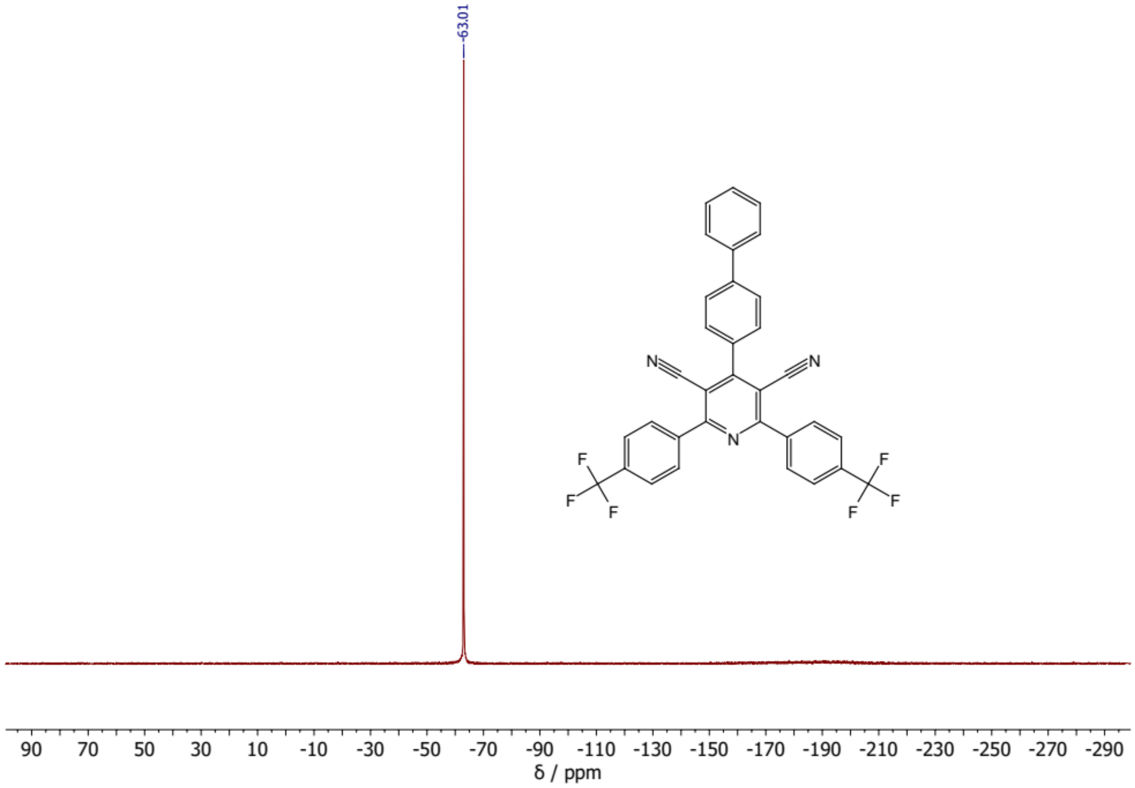

Figure S59. $^{19}F\{^1H\}$-NMR spectrum of **PhPyCF3** in $CDCl_3$.

9.2. Mass Spectra

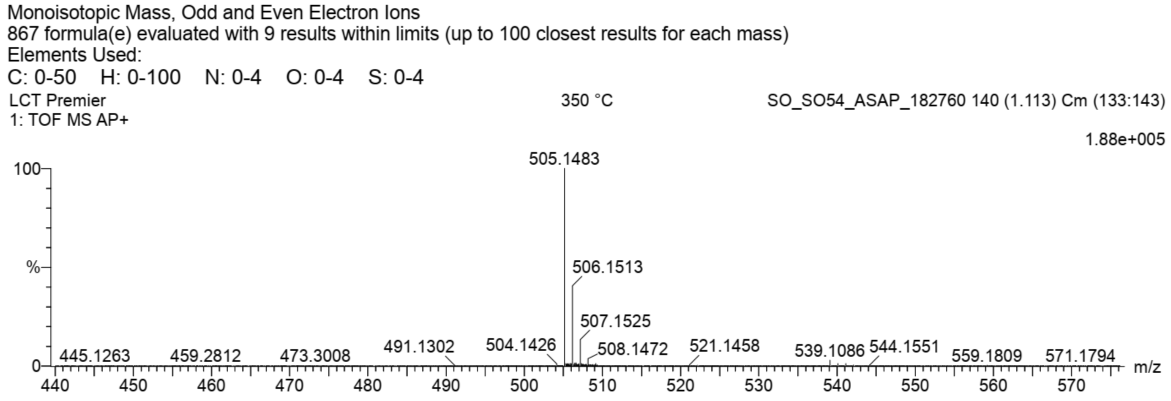

Figure S60. HRMS spectrum of **TCz-Me**.

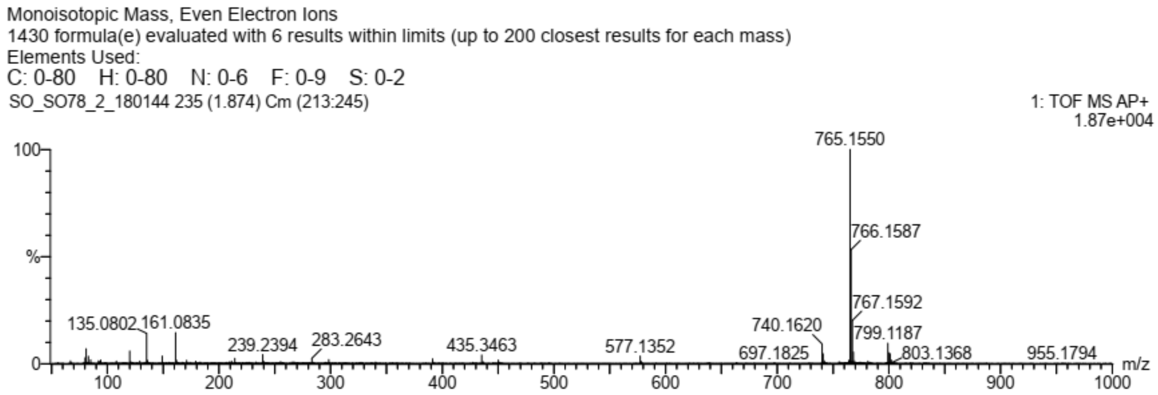

Figure S61. HRMS spectrum of **TCz-CF3**.

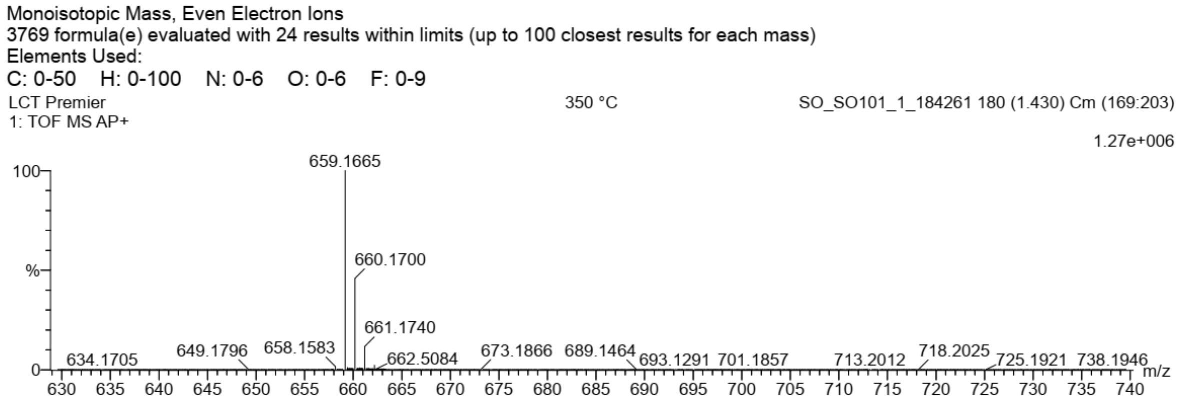

Figure S62. HRMS spectrum of **Cz-CF3**.

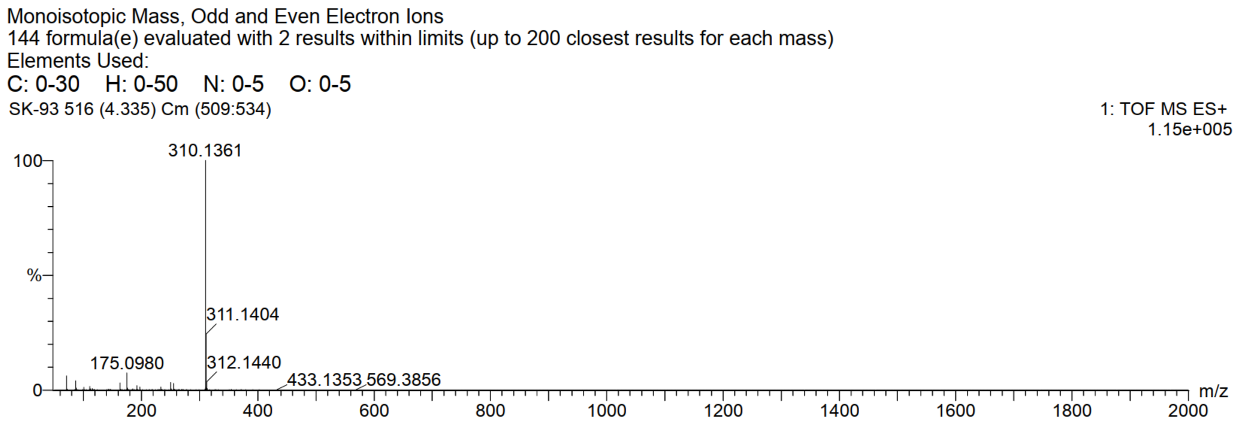

Figure S63. ESI-HRMS spectrum of **PhPyMe**.

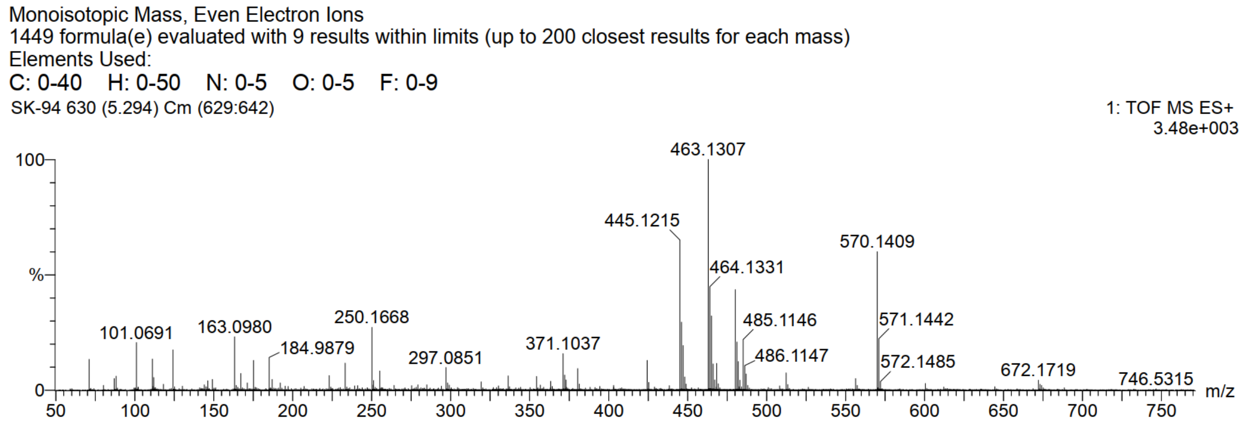

Figure S64. ESI-HRMS spectrum of **PhPyCF3**.

10. Cartesian Coordinates

TCz-Me S_1 optimized geometry

$C_{33}H_{20}N_4S$

B3LYP/6-31G(d) E(TD-HF/TD-DFT) = -1886.70244 a.u.

58 atoms

```
C 1.54457900 -1.03594800 1.20621800
C 2.22625900 -0.83160900 0.00013600
C 1.54468000 -1.03650600 -1.20593700
C 0.19214600 -1.37349700 -1.21551400
C -0.47949700 -1.50888100 0.00018200
C 0.19203900 -1.37299500 1.21585300
H 2.07370400 -0.90375300 2.14413200
H 2.07393700 -0.90482100 -2.14384500
H -0.34524300 -1.49258400 -2.15166200
H -0.34535000 -1.49175300 2.15204200
C -2.64007000 -2.86258600 0.00018400
C -2.78922700 -0.61514500 -0.00007600
C -2.14927400 -4.16547900 0.00039100
C -4.02376100 -2.56445000 0.00003500
C -2.53739000 0.77849200 -0.00014300
C -4.13420200 -1.11351700 -0.00015700
C -3.09138900 -5.19624400 0.00043800
H -1.08347400 -4.36618600 0.00050100
C -4.94562700 -3.61072600 0.00006300
C -3.64401700 1.67458200 -0.00026400
C -5.19295300 -0.22059900 -0.00022100
C -4.46732500 -4.92496300 0.00027400
H -2.74879200 -6.22605200 0.00059900
H -6.01388200 -3.41451600 -0.00008500
C -4.94524300 1.17269800 -0.00028600
H -6.21781200 -0.57827300 -0.00022200
H -5.17365600 -5.74917500 0.00029900
H -5.78810700 1.85644700 -0.00032900
```

```
N -1.91635600 -1.65962200 0.00008700
C 3.64222600 -0.34994800 0.00006200
C 4.70439200 -1.24649100 -0.00024400
C 3.89520100 1.04750700 0.00027900
C 6.06697800 -0.73457800 -0.00026300
C 5.30308400 1.46795500 0.00019200
N 6.29009600 0.59226600 -0.00004200
C 4.44926600 -2.64579600 -0.00059100
C 2.84723100 1.98262200 0.00048400
N 1.97374400 2.77286100 0.00070900
N 4.24672800 -3.79735000 -0.00089800
C -3.19316700 3.06300000 -0.00024000
C -3.93623500 4.24733500 -0.00040300
C -1.78214400 3.15364800 -0.00005700
C -3.26688900 5.47227000 -0.00035400
H -5.02224100 4.21977500 -0.00056100
C -1.09769600 4.37199100 0.00001300
C -1.86527700 5.53502900 -0.00014000
H -3.84361100 6.39228600 -0.00048500
H -0.01210600 4.38373200 0.00021000
H -1.37021000 6.50091600 -0.00009100
S -1.00595100 1.57686400 0.00001500
C 5.64117400 2.93112300 0.00052100
H 5.22668800 3.44638500 -0.87844900
H 5.22820400 3.44562700 0.88066700
H 6.72715700 3.05015000 -0.00031500
C 7.24606200 -1.66172700 -0.00045600
H 7.26131700 -2.32416100 0.87842600
H 7.26218000 -2.32283100 -0.88033700
H 8.16342800 -1.06751300 0.00040400
```

TCz-CF3 S_1 optimized geometry

$C_{45}H_{22}N_4F_6S$

B3LYP/6-31G(d) E(TD-HF/TD-DFT) = -2944.26727 a.u.

78 atoms

C -1.25015800 -1.10731900 1.41297300
C -0.52876600 -1.17083000 0.21483600
C -1.16569400 -1.64057800 -0.94004500
C -2.51667500 -1.98104300 -0.92029100
C -3.22831600 -1.85891800 0.27384100
C -2.59982700 -1.45199600 1.45140500
H -0.75564000 -0.76678500 2.31667900
H -0.60383700 -1.71918500 -1.86484900
H -3.02045500 -2.30811900 -1.82483200
H -3.16718500 -1.37413000 2.37395600
C -5.34964300 -3.25411700 0.46951000
C -5.55996800 -1.05257300 0.03381800
C -4.82300500 -4.51501400 0.73535900
C -6.73920800 -3.00918100 0.36444400
C -5.34776300 0.32317800 -0.22504100
C -6.88962400 -1.58937900 0.08224500
C -5.73604000 -5.55918900 0.89986500
H -3.75312700 -4.67628300 0.81021500
C -7.63139000 -4.06723400 0.53264800
C -6.47715100 1.16262600 -0.43800600
C -7.97179900 -0.75175100 -0.12823100
C -7.11734400 -5.34045800 0.80059500
H -5.36555700 -6.55763000 1.10832900
H -8.70365700 -3.91193200 0.45809900
C -7.76289800 0.62357700 -0.38834500
H -8.98529700 -1.13876800 -0.09643700
H -7.80028700 -6.17343700 0.93365400
H -8.62368900 1.26432400 -0.55020900
N -4.65976100 -2.04727400 0.26458100
C 0.89627700 -0.71225600 0.16007300
C 1.93152600 -1.65215900 0.16887500
C 1.16978400 0.67033600 0.09729200
C 3.30634600 -1.16320800 0.03824400
C 2.57632400 1.07518600 0.14313200
N 3.53099000 0.15635800 0.07727300
C 1.58371400 -3.01238900 0.38404500
C 0.09817900 1.57279000 -0.10176700
N -0.80634100 2.29844000 -0.26924100
N 1.24509200 -4.11735900 0.56139600
C -6.06752300 2.54137000 -0.68617700
C -6.84473800 3.67563800 -0.94137800
C -4.66151600 2.68325200 -0.64749900
C -6.21342800 4.90265400 -1.14803900
H -7.92817000 3.60725300 -0.97929300
C -4.01596600 3.90560400 -0.85094000
C -4.81564800 5.01794400 -1.10326800
H -6.81628800 5.78349300 -1.34639900
H -2.93323700 3.96672400 -0.80806700
H -4.34982900 5.98443000 -1.26648600
S -3.84128800 1.16407400 -0.31640000
C 4.48614200 -2.01653400 -0.11492300
C 4.42837600 -3.34810500 -0.58615200
C 5.76781700 -1.48846800 0.17979400
C 5.58204700 -4.10791500 -0.74121400
H 3.47964300 -3.79484500 -0.85391800
C 6.91535900 -2.24772000 0.02630800
H 5.83336300 -0.46748500 0.53635400
C 6.83524200 -3.56967900 -0.43652200
H 5.50852800 -5.12368200 -1.11605300
H 7.88386100 -1.81798400 0.26302100
C 3.02653800 2.47228600 0.25279400
C 2.24465600 3.48845200 0.84039000
C 4.31597200 2.82310600 -0.20737500
C 2.72264200 4.79004100 0.95058000
H 1.26311500 3.26153600 1.23779300
C 4.79098200 4.12092500 -0.10093500
H 4.93174000 2.04963500 -0.65142200
C 3.99563300 5.11930000 0.47879200
H 2.10882200 5.55158800 1.42067000
H 5.78372400 4.36639800 -0.46507900
C 4.48209100 6.53459000 0.53378600
C 8.07050000 -4.40734000 -0.54141700
F 7.94792600 -5.39002700 -1.46663200
F 8.38052900 -5.02347100 0.62985400
F 9.15838600 -3.67231300 -0.87989000
F 5.82682000 6.60756900 0.67645200
F 3.93113700 7.22611400 1.56093700
F 4.18096300 7.22696400 -0.59626400

Cz-CF3 S_1 optimized geometry

$C_{39}H_{20}N_4F_6$

B3LYP/6-31G(d) E(TD-HF/TD-DFT) = -2316.20248 a.u.

69 atoms

C 2.16880100 -0.44574900 1.12139900
C 1.46097600 -0.00004000 -0.00003500
C 2.16881600 0.44565800 -1.12146400
C 3.56174400 0.44914900 -1.13162100
C 4.23890000 -0.00005100 -0.00002000
C 3.56172800 -0.44924700 1.13157300
H 1.62453000 -0.79470200 1.99213100
H 1.62455700 0.79461900 -1.99220000
H 4.11085800 0.78730200 -2.00503800
H 4.11083100 -0.78739900 2.00499800
C 6.48294900 1.11183700 0.18781600
C 6.48299100 -1.11185300 -0.18781200
C 6.07932000 2.43593300 0.42158700
C 7.85118200 0.72092200 0.12145600
C 6.07941500 -2.43597400 -0.42158500
C 7.85121300 -0.72088500 -0.12142900
C 7.08673800 3.38471000 0.58493000
H 5.02968700 2.70098800 0.47241100
C 8.83232400 1.68090300 0.28781200
C 7.08686600 -3.38470700 -0.58490600
C 8.83239100 -1.68083200 -0.28776500
C 8.43757600 3.01760200 0.51889700
H 6.81966700 4.41946300 0.76709900
H 9.88621800 1.42516500 0.24562500
C 8.43769600 -3.01754100 -0.51885100
H 9.88627500 -1.42505300 -0.24556300
H 9.20218900 3.77592900 0.64999000
H 9.20233700 -3.77584300 -0.64992900
N 5.68794600 -0.00002900 -0.00000300
C -0.03681700 -0.00001800 -0.00003400
C -0.72327900 1.22026500 0.09763000
C -0.72330500 -1.22029700 -0.09769100
C -2.18554300 1.17965300 0.01916300
C -2.18555900 -1.17962800 -0.01918200
N -2.80587000 -0.00000200 0.00000800
C 0.03843700 2.38975800 0.34695200
C 0.03834700 -2.38982500 -0.34701800
N 0.72097000 -3.31973300 -0.54553200
N 0.72112900 3.31961200 0.54547600
C -3.04846900 2.36631600 -0.02739100
C -2.59988900 3.63599300 -0.45294400
C -4.41254800 2.24597300 0.33173500
C -3.46394300 4.72436800 -0.50569200
H -1.57322000 3.77863700 -0.76486600
C -5.27171700 3.33110200 0.28049100
H -4.77523600 1.27735600 0.65469900
C -4.80489200 4.58549700 -0.13909600
H -3.09566600 5.68606400 -0.84803200
H -6.31275600 3.21108400 0.56422100
C -3.04851500 -2.36630200 0.02737800
C -2.59998000 -3.63592900 0.45310200
C -4.41253800 -2.24596600 -0.33189900
C -3.46404900 -4.72429300 0.50586700
H -1.57334100 -3.77853700 0.76514100
C -5.27173100 -3.33108600 -0.28064200
H -4.77518100 -1.27737200 -0.65498300
C -4.80495700 -4.58543500 0.13911200
H -3.09582500 -5.68596000 0.84834400
H -6.31273800 -3.21108700 -0.56449300
C -5.71927200 -5.77015600 0.13237900
C -5.71922300 5.77019600 -0.13234600
F -5.34138600 6.71594400 -1.02667800
F -5.76024000 6.38606700 1.07869500
F -6.99806600 5.42993300 -0.42586300
F -6.99821500 -5.42984200 0.42536700
F -5.34171100 -6.71564200 1.02710600
F -5.75984400 -6.38636800 -1.07849900
H 5.02979100 -2.70106100 -0.47242400
H 6.81984600 -4.41947200 -0.76707600

11. References

(1) Fang, J.; Li, L.; Yang, C.; Chen, J.; Deng, G.-J.; Gong, H. Tandem Oxidative Ring-Opening/Cyclization Reaction in Seconds in Open Atmosphere for the Synthesis of 1-Tetralones in Water–Acetonitrile. *Org. Lett.* **2018**, *20*, 7308–7311.

(2) Li, J.; Chen, W.-C.; Liu, H.; Chen, Z.; Chai, D.; Lee, C.-S.; Yang, C. Double-Twist Pyridine–Carbonitrile Derivatives Yielding Excellent Thermally Activated Delayed Fluorescence Emitters for High-Performance OLEDs. *J. Mater. Chem. C* **2020**, *8*, 602–606.

(3) Havel, S.; Khirsariya, P.; Akavaram, N.; Paruch, K.; Carbain, B. Preparation of 3,4-Substituted-5-Aminopyrazoles and 4-Substituted-2-Aminothiazoles. *J. Org. Chem.* **2018**, *83*, 15380–15405.

(4) Pommerehne, J.; Vestweber, H.; Guss, W.; Mahrt, R. F.; Bässler, H.; Porsch, M.; Daub, J. Efficient Two Layer Leds on a Polymer Blend Basis. *Adv. Mater.* **1995**, *7*, 551-554.

(5) Djurovich, P. I.; Mayo, E. I.; Forrest, S. R.; Thompson, M. E. Measurement of the Lowest Unoccupied Molecular Orbital Energies of Molecular Organic Semiconductors. *Org. Electron*. **2009**, *10*, 515-520.

(6) Vigante, B.; Leitonas, K.; Volyniuk, D.; Andruleviciene, V.; Simokaitiene, J.; Ivanova, A.; Bucinskas, A.; Grazulevicius, J. V.; Arsenyan, P. Synthesis of Linear and V-Shaped Carbazolyl-Substituted Pyridine-3, 5-Dicarbonitriles Exhibiting Efficient Bipolar Charge Transport and E-Type Fluorescence. *Chem. Eur. J.* **2019**, *25*, 3325-3336.

(7) Liu, H.; Li, J.; Li, G.; Zhang, B.; Zhan, Q.; Liu, Z.; Zhou, C.; Li, K.; Wang, Z.; Yang, C. A Simple Strategy to Achieve Efficient Thermally Activated Delayed Fluorescent Emitters *via* Enhancing Electron Donating Ability of Donors. *Dyes Pigm.* **2020**, *180*, 108521.
